# Supplementary material for: Comprehensive transcriptomic analysis of prostate cancer lung metastases
Source: PLoS One. 2024 Aug 15;19(8):e0306525. doi: 10.1371/journal.pone.0306525 (PMC11326543; doi:10.1371/journal.pone.0306525)
Supplement: S1 Table — (PDF) [file pone.0306525.s001.pdf]

Probe Nam	Annotation	Accession #	NS Probe II	Class Name	Analyte Ty	Positive Fla	LM	StDev of LM
FOXC2		NM_005251.2		Endogenou	mRNA	FALSCH	5,28	2,06
HLA-DPB1		NM_002121.4		Endogenou	mRNA	FALSCH	1028,91	705,68
PTPRC		NM_080923.2		Endogenou	mRNA	FALSCH	98,99	69,85
ITGB7		NM_000889.1		Endogenou	mRNA	FALSCH	34,02	23,14
CCL5		NM_002985.2		Endogenou	mRNA	FALSCH	180,06	163,53
CCL21		NM_002989.2		Endogenou	mRNA	FALSCH	91,8	179,42
C3		NM_000064.2		Endogenou	mRNA	FALSCH	339,19	746,07
SERPINA1		NM_000295.4		Endogenou	mRNA	FALSCH	214,28	291,78
MFAP4		NM_002404.1		Endogenou	mRNA	FALSCH	551,84	1515,71
ITGAM		NM_000632.3		Endogenou	mRNA	FALSCH	50,44	53,71
ARAP2		NM_015230.2		Endogenou	mRNA	FALSCH	95,75	75,26
PRF1		NM_005041.3		Endogenou	mRNA	FALSCH	102,81	140,07
PDCL3		NM_024065.4		Endogenou	mRNA	FALSCH	146,85	44,27
PRKCB		NM_212535.1		Endogenou	mRNA	FALSCH	47,52	28,94
STAT1		NM_139266.1		Endogenou	mRNA	FALSCH	1059,22	574
CXCR3		NM_001504.1		Endogenou	mRNA	FALSCH	29,28	18,67
PLA2G10		NM_003561.1		Endogenou	mRNA	FALSCH	18,11	33,09
CFP		NM_002621.2		Endogenou	mRNA	FALSCH	15,57	9,39
TWIST1		NM_000474.3		Endogenou	mRNA	FALSCH	22,64	48,22
SPHK2		NM_020126.3		Endogenou	mRNA	FALSCH	132,99	46,28
ADAMTS8		NM_007037.4		Endogenou	mRNA	FALSCH	30,27	81,07
CASP8		NM_001228.4		Endogenou	mRNA	FALSCH	192,75	49,41
HSP90B1		NM_003299.1		Endogenou	mRNA	FALSCH	750,14	220,5
PIK3R2		NM_005027.2		Endogenou	mRNA	FALSCH	200,23	60,59
CHAD		NM_001267.2		Endogenou	mRNA	FALSCH	5,73	4,25
CDK14		NM_012395.2		Endogenou	mRNA	FALSCH	89,95	38,38
S100A14		NM_020672.1		Endogenou	mRNA	FALSCH	24,78	133,07
IBSP		NM_004967.3		Endogenou	mRNA	FALSCH	8,31	35,87
CST7		NM_003650.3		Endogenou	mRNA	FALSCH	37,37	48,89
STAB2		NM_017564.9		Endogenou	mRNA	FALSCH	6,53	4,71
ITGB6		NM_000888.3		Endogenou	mRNA	FALSCH	40,34	84,48
CCL11		NM_002986.2		Endogenou	mRNA	FALSCH	12,74	8,01
TBX4		NM_018488.2		Endogenou	mRNA	FALSCH	31,11	92,79
EPN3		NM_017957.2		Endogenou	mRNA	FALSCH	78,1	60,29
FGL2		NM_006682.2		Endogenou	mRNA	FALSCH	91,24	51,26
EGLN3		NM_022073.3		Endogenou	mRNA	FALSCH	87,12	60,72
TCF3		NM_003200.3		Endogenou	mRNA	FALSCH	59,63	25,25
CXCL10		NM_001565.1		Endogenou	mRNA	FALSCH	114,9	174,35
RPS6KB1		NM_003161.2		Endogenou	mRNA	FALSCH	271,59	56,72
CHRDL1		NM_001143981.1		Endogenou	mRNA	FALSCH	130,99	181,21
CSF2RB		NM_000395.2		Endogenou	mRNA	FALSCH	22,73	15
ALOX5		NM_000698.2		Endogenou	mRNA	FALSCH	361,05	266,25
IL10RA		NM_001558.2		Endogenou	mRNA	FALSCH	138,19	77,03
ZFPM2		NM_012082.3		Endogenou	mRNA	FALSCH	11,63	11,16
FGFR4		NM_002011.3		Endogenou	mRNA	FALSCH	68,03	107,97
GIMAP4		NM_018326.2		Endogenou	mRNA	FALSCH	256,23	241,72
ADD1		NM_001119.4		Endogenou	mRNA	FALSCH	443,73	95,95

ADAM15	NM_207195.1	Endogenou	mRNA	FALSCH	690,91	306,58
CCR2	NM_001123041.2	Endogenou	mRNA	FALSCH	32,47	23,91
TNF	NM_000594.2	Endogenou	mRNA	FALSCH	25,8	19,6
KRT7	NM_005556.3	Endogenou	mRNA	FALSCH	100,54	833,84
MAPK3	NM_001040056.1	Endogenou	mRNA	FALSCH	343,12	109,53
FASLG	NM_000639.1	Endogenou	mRNA	FALSCH	10,91	8,51
AAMP	NM_001087.3	Endogenou	mRNA	FALSCH	425,14	101,62
CXCL17	NM_198477.1	Endogenou	mRNA	FALSCH	76,56	234,87
CHI3L1	NM_001276.2	Endogenou	mRNA	FALSCH	67,52	313,86
MTA1	NM_004689.2	Endogenou	mRNA	FALSCH	181,62	65,37
FIGF	NM_004469.2	Endogenou	mRNA	FALSCH	56,16	219,33
VIT	NM_053276.3	Endogenou	mRNA	FALSCH	12,82	4,66
PIK3CD	NM_005026.3	Endogenou	mRNA	FALSCH	56,8	36,84
WARS	NM_004184.3	Endogenou	mRNA	FALSCH	352,44	232,39
MAPK1	NM_138957.2	Endogenou	mRNA	FALSCH	278,08	81,86
TPSB2	NM_024164.5	Endogenou	mRNA	FALSCH	447,03	1233,24
SAMSN1	NM_022136.3	Endogenou	mRNA	FALSCH	59,27	49,34
CYB561	NM_001915.3	Endogenou	mRNA	FALSCH	551,08	208,89
SMAD2	NM_005901.5	Endogenou	mRNA	FALSCH	305,68	124,34
TMEM100	NM_018286.2	Endogenou	mRNA	FALSCH	66,52	319,34
ADAMTS12	NM_030955.2	Endogenou	mRNA	FALSCH	49,63	41,21
MUC1	NM_001018017.1	Endogenou	mRNA	FALSCH	325,68	840,58
MAP2K2	NM_030662.3	Endogenou	mRNA	FALSCH	233,47	87,23
HAPLN1	NM_001884.3	Endogenou	mRNA	FALSCH	5,43	3,42
RPS6KB2	NM_003952.2	Endogenou	mRNA	FALSCH	86,67	18,3
GIMAP6	NR_024115.1	Endogenou	mRNA	FALSCH	71,6	68,63
FGF2	NM_002006.4	Endogenou	mRNA	FALSCH	47,57	76,28
SLC44A4	NM_032794.1	Endogenou	mRNA	FALSCH	164,09	211,85
GTF2I	NM_033001.2	Endogenou	mRNA	FALSCH	985,74	262,2
AKT1	NM_005163.2	Endogenou	mRNA	FALSCH	1363,64	472,47
HKDC1	NM_025130.3	Endogenou	mRNA	FALSCH	9,53	35,66
SPARCL1	NM_004684.4	Endogenou	mRNA	FALSCH	731,68	850,1
IL18	NM_001562.2	Endogenou	mRNA	FALSCH	68,05	116,8
RBM47	NM_019027.3	Endogenou	mRNA	FALSCH	339,43	125,97
TBX1	NM_080646.1	Endogenou	mRNA	FALSCH	46,01	36,98
CEACAM6	NM_002483.4	Endogenou	mRNA	FALSCH	151,75	573,63
IL1B	NM_000576.2	Endogenou	mRNA	FALSCH	26,35	32,04
COMP	NM_000095.2	Endogenou	mRNA	FALSCH	15,96	54,67
CDC42	NM_001039802.1	Endogenou	mRNA	FALSCH	875,11	208,42
VAMP8	NM_003761.3	Endogenou	mRNA	FALSCH	167,51	57,64
RAC2	NM_002872.3	Endogenou	mRNA	FALSCH	130,74	96,78
PEBP4	NM_144962.2	Endogenou	mRNA	FALSCH	44,57	108,69
COL1A1	NM_000088.3	Endogenou	mRNA	FALSCH	3309,96	9525,44
MRPS5	NM_031902.3	Housekeep	mRNA	FALSCH	255,24	47,36
AGRN	NM_198576.2	Endogenou	mRNA	FALSCH	378,8	146,92
KRAS	NM_004985.3	Endogenou	mRNA	FALSCH	236,92	52,01
ITM2A	NM_004867.4	Endogenou	mRNA	FALSCH	126,19	164,06
MET	NM_001127500.1	Endogenou	mRNA	FALSCH	51,43	1093,17

INHBE	NM_031479.3	Endogenou	mRNA	FALSCH	5,57	4,4
ANG	NM_001145.4	Endogenou	mRNA	FALSCH	90,29	55,62
PPL	NM_002705.4	Endogenou	mRNA	FALSCH	148,91	97,2
GZMK	NM_002104.2	Endogenou	mRNA	FALSCH	46,88	39,83
ITGA3	NM_002204.2	Endogenou	mRNA	FALSCH	268,37	731,57
PLXNC1	NM_005761.2	Endogenou	mRNA	FALSCH	98,56	70,4
TEK	NM_000459.3	Endogenou	mRNA	FALSCH	49,15	95,76
ITGB2	NM_001127491.1	Endogenou	mRNA	FALSCH	243,03	183,98
SF3A3	NM_006802.2	Housekeep	mRNA	FALSCH	257,9	41,54
VEZF1	NM_007146.2	Endogenou	mRNA	FALSCH	194,65	53,21
CCL7	NM_006273.2	Endogenou	mRNA	FALSCH	5,02	2,68
FBLN5	NM_006329.3	Endogenou	mRNA	FALSCH	132,17	192,26
PLA2G2D	NM_001271814.1	Endogenou	mRNA	FALSCH	11,32	14,69
TNFSF10	NM_003810.2	Endogenou	mRNA	FALSCH	817,35	474,48
CYBB	NM_000397.3	Endogenou	mRNA	FALSCH	278,23	375,34
CAV1	NM_001753.3	Endogenou	mRNA	FALSCH	320,48	885,77
GDF5	NM_000557.2	Endogenou	mRNA	FALSCH	5,69	5,48
SSTR2	NM_001050.2	Endogenou	mRNA	FALSCH	7,64	8,44
COL4A1	NM_001845.4	Endogenou	mRNA	FALSCH	842,94	522,2
SLC12A6	NM_001042494.1	Endogenou	mRNA	FALSCH	73,05	39,81
MRC1	NM_002438.2	Endogenou	mRNA	FALSCH	238,78	263,94
SET	NM_001122821.1	Endogenou	mRNA	FALSCH	385,84	80,71
MYC	NM_002467.3	Endogenou	mRNA	FALSCH	457,59	567,11
OLFML2B	NM_015441.1	Endogenou	mRNA	FALSCH	80,98	69,79
MYH11	NM_001040113.1	Endogenou	mRNA	FALSCH	145,45	317,14
PIK3CG	NM_002649.2	Endogenou	mRNA	FALSCH	66,71	44,8
PPP2CB	NM_001009552.1	Endogenou	mRNA	FALSCH	621,73	284,92
AHNAK	NM_001620.2	Endogenou	mRNA	FALSCH	1819,9	1098,18
SRGN	NR_036430.1	Endogenou	mRNA	FALSCH	764,63	1189,85
CCL8	NM_005623.2	Endogenou	mRNA	FALSCH	18,82	28,04
CCBE1	NM_133459.3	Endogenou	mRNA	FALSCH	33,57	74,34
LAMB3	NM_000228.2	Endogenou	mRNA	FALSCH	43,38	199,63
KRT19	NM_002276.4	Endogenou	mRNA	FALSCH	497,17	735,17
TCEB2	NM_007108.2	Endogenou	mRNA	FALSCH	28,08	13,97
GLYR1	NM_032569.3	Endogenou	mRNA	FALSCH	295,43	50,15
PTGDS	NM_000954.5	Endogenou	mRNA	FALSCH	233,41	528,69
IL1RL1	NM_016232.4	Endogenou	mRNA	FALSCH	28,95	82,84
SLPI	NM_003064.2	Endogenou	mRNA	FALSCH	356,2	542,92
IL1A	NM_000575.3	Endogenou	mRNA	FALSCH	7,63	5,61
CSPG4	NM_001897.4	Endogenou	mRNA	FALSCH	77,65	76,91
ACTG2	NM_001615.3	Endogenou	mRNA	FALSCH	72,91	96,07
TNS1	NM_022648.4	Endogenou	mRNA	FALSCH	482,05	696,09
EDC3	NM_001142443.1	Housekeep	mRNA	FALSCH	195,35	38,76
ITGA8	NM_003638.1	Endogenou	mRNA	FALSCH	46,34	109,29
ANGPT1	NM_001146.3	Endogenou	mRNA	FALSCH	95,8	221,98
CD82	NM_002231.3	Endogenou	mRNA	FALSCH	132,23	77,87
C3AR1	NM_004054.2	Endogenou	mRNA	FALSCH	99,3	101,21
CTSH	NM_148979.2	Endogenou	mRNA	FALSCH	504,52	526,77

COL1A2	NM_000089.3	Endogenou	mRNA	FALSCH	1373,26	2433,92
EPHB3	NM_004443.3	Endogenou	mRNA	FALSCH	73,46	70,5
MMP9	NM_004994.2	Endogenou	mRNA	FALSCH	88,51	432,96
PTK2B	NM_004103.3	Endogenou	mRNA	FALSCH	143,36	103,88
TFPI2	NM_006528.3	Endogenou	mRNA	FALSCH	26,24	176,26
EIF2AK3	NM_004836.5	Endogenou	mRNA	FALSCH	155,3	50,93
EDN1	NM_001955.2	Endogenou	mRNA	FALSCH	84,32	115,98
RB1	NM_000321.1	Endogenou	mRNA	FALSCH	163,23	86,61
ACVRL1	NM_000020.1	Endogenou	mRNA	FALSCH	187,05	348,84
IFNG	NM_000619.2	Endogenou	mRNA	FALSCH	10,19	10,25
CUL1	NM_003592.2	Endogenou	mRNA	FALSCH	206	32,74
PIK3R5	NM_001142633.1	Endogenou	mRNA	FALSCH	46,17	28,18
C1S	NM_001734.2	Endogenou	mRNA	FALSCH	331,32	313,51
EPAS1	NM_001430.3	Endogenou	mRNA	FALSCH	508,42	1033,3
ICAM1	NM_000201.2	Endogenou	mRNA	FALSCH	238,66	294,49
SERPING1	NM_000062.2	Endogenou	mRNA	FALSCH	716,11	668,99
ADAM28	NM_014265.4	Endogenou	mRNA	FALSCH	48,4	72,21
CYP1B1	NM_000104.3	Endogenou	mRNA	FALSCH	67,52	142,77
TYMP	NM_001953.3	Endogenou	mRNA	FALSCH	256,54	194,89
RRAS	NM_006270.3	Endogenou	mRNA	FALSCH	101,6	93,88
SPARC	NM_003118.2	Endogenou	mRNA	FALSCH	2333,64	2433,37
PCOLCE	NM_002593.3	Endogenou	mRNA	FALSCH	199,73	221,96
CXCL11	NM_005409.3	Endogenou	mRNA	FALSCH	34,09	123,54
NOL7	NM_016167.3	Housekeep	mRNA	FALSCH	269,57	95,64
SEMA3E	NM_012431.1	Endogenou	mRNA	FALSCH	51,73	87,11
PTPRB	NM_002837.3	Endogenou	mRNA	FALSCH	115,27	163,9
BNC2	NM_017637.5	Endogenou	mRNA	FALSCH	17,06	12,16
KIAA1462	NM_020848.2	Endogenou	mRNA	FALSCH	110,94	142,69
EVI2A	NM_014210.3	Endogenou	mRNA	FALSCH	61,04	31,86
ERCC3	NM_000122.1	Housekeep	mRNA	FALSCH	82,13	17,52
ILK	NM_004517.2	Endogenou	mRNA	FALSCH	214,36	90,42
NCAM1	NM_000615.5	Endogenou	mRNA	FALSCH	16,59	9,87
BAD	NM_004322.3	Endogenou	mRNA	FALSCH	90,74	28,7
BMPR1B	NM_001203.1	Endogenou	mRNA	FALSCH	202,38	413,83
SERPINH1	NM_001235.2	Endogenou	mRNA	FALSCH	707,31	520,15
AKAP12	NM_005100.3	Endogenou	mRNA	FALSCH	256,82	205,83
FSTL1	NM_007085.4	Endogenou	mRNA	FALSCH	1231,16	1004,11
ROCK2	NM_004850.3	Endogenou	mRNA	FALSCH	395,87	120,22
GJA5	NM_005266.5	Endogenou	mRNA	FALSCH	42,61	67,19
USP39	NM_001256725.1	Housekeep	mRNA	FALSCH	102,47	23,64
NME4	NM_005009.2	Endogenou	mRNA	FALSCH	335,01	198,23
CNOT10	NM_001256741.1	Housekeep	mRNA	FALSCH	190,62	38,08
ANXA2P2	NR_003573.1	Endogenou	mRNA	FALSCH	1804,65	1662,9
NAA15	NM_057175.3	Endogenou	mRNA	FALSCH	97	18,42
KRT14	NM_000526.4	Endogenou	mRNA	FALSCH	9,44	67,35
PDPN	NM_006474.4	Endogenou	mRNA	FALSCH	79,44	107,15
PTPRM	NM_002845.3	Endogenou	mRNA	FALSCH	260,83	192,75
SCNN1A	NM_001038.4	Endogenou	mRNA	FALSCH	185,65	197,21

STAB1	NM_015136.2	Endogenou	mRNA	FALSCH	91,85	113,25
RORB	NM_006914.3	Endogenou	mRNA	FALSCH	16,68	81,49
BRMS1	NM_015399.3	Endogenou	mRNA	FALSCH	112,84	21,74
VCAM1	NM_001078.3	Endogenou	mRNA	FALSCH	91,11	431,6
WIPF1	NM_001077269.1	Endogenou	mRNA	FALSCH	129,29	66,39
HEG1	NM_020733.1	Endogenou	mRNA	FALSCH	208	221,86
CTSL	NM_001912.4	Endogenou	mRNA	FALSCH	272,9	957,58
CXCL13	NM_006419.2	Endogenou	mRNA	FALSCH	18,6	24,83
PECAM1	NM_000442.3	Endogenou	mRNA	FALSCH	618,47	831,08
EMCN	NM_016242.3	Endogenou	mRNA	FALSCH	76,82	130,33
NFKB1	NM_003998.2	Endogenou	mRNA	FALSCH	43,74	20,57
SP1	NM_003109.1	Endogenou	mRNA	FALSCH	180,19	35,65
CLEC2B	NM_005127.2	Endogenou	mRNA	FALSCH	262,74	212,78
PLCG2	NM_002661.2	Endogenou	mRNA	FALSCH	56,96	40,98
LAMC1	NM_002293.3	Endogenou	mRNA	FALSCH	402,68	252,36
COL4A2	NM_001846.2	Endogenou	mRNA	FALSCH	754,52	442,67
SV2B	NM_001167580.1	Endogenou	mRNA	FALSCH	19,8	91,37
JUN	NM_002228.3	Endogenou	mRNA	FALSCH	641,56	596,98
KDM1A	NM_015013.3	Endogenou	mRNA	FALSCH	169,43	50,99
PPP1R16B	NM_015568.2	Endogenou	mRNA	FALSCH	33,72	28,47
RBPJ	NM_015874.3	Endogenou	mRNA	FALSCH	338,84	83,27
MMP13	NM_002427.2	Endogenou	mRNA	FALSCH	6,18	74,23
DHX16	NM_001164239.1	Housekeep	mRNA	FALSCH	118,44	17,46
IL6	NM_000600.1	Endogenou	mRNA	FALSCH	13,67	12,89
ITGA1	NM_181501.1	Endogenou	mRNA	FALSCH	258,85	186,88
PDGFC	NM_016205.2	Endogenou	mRNA	FALSCH	44,61	100,47
ZFYVE16	NM_001105251.2	Endogenou	mRNA	FALSCH	198,62	42,94
TJP3	NM_014428.1	Endogenou	mRNA	FALSCH	84,43	138,12
SNAI3	NM_178310.1	Endogenou	mRNA	FALSCH	12,68	6,98
HK3	NM_002115.1	Endogenou	mRNA	FALSCH	20,69	21,42
IL15	NM_172174.1	Endogenou	mRNA	FALSCH	28,52	21,51
COG7	NM_153603.3	Housekeep	mRNA	FALSCH	244,64	35,16
LAMC2	NM_005562.2	Endogenou	mRNA	FALSCH	49,55	160,87
VAV2	NM_003371.3	Endogenou	mRNA	FALSCH	87,64	23,01
TGFBR2	NM_001024847.1	Endogenou	mRNA	FALSCH	425,39	444,23
HOXA5	NM_019102.2	Endogenou	mRNA	FALSCH	53,82	44,7
CLDN3	NM_001306.3	Endogenou	mRNA	FALSCH	396,95	383,52
FLI1	NM_001167681.2	Endogenou	mRNA	FALSCH	89,38	75,98
NUBP1	NM_001278506.1	Housekeep	mRNA	FALSCH	47,12	9,97
TNFSF12	NM_003809.2	Endogenou	mRNA	FALSCH	189,67	96,52
CXADR	NM_001338.3	Endogenou	mRNA	FALSCH	248,99	186,94
COL3A1	NM_000090.3	Endogenou	mRNA	FALSCH	3822,99	8459,5
BGN	NM_001711.3	Endogenou	mRNA	FALSCH	460,43	368,79
LY96	NM_015364.2	Endogenou	mRNA	FALSCH	67,81	58,85
ADM2	NM_001253845.1	Endogenou	mRNA	FALSCH	30,78	43,64
HSPG2	NM_005529.5	Endogenou	mRNA	FALSCH	872,97	491,94
COL5A2	NM_000393.3	Endogenou	mRNA	FALSCH	250,01	446,5
DPYSL3	NM_001387.2	Endogenou	mRNA	FALSCH	338,16	168,51

TMPRSS6	NM_153609.2	Endogenou	mRNA	FALSCH	4,67	0,78
FREM2	NM_207361.4	Endogenou	mRNA	FALSCH	15,62	29,16
MTMR14	NM_022485.3	Housekeep	mRNA	FALSCH	216,13	42,59
PTRF	NM_012232.5	Endogenou	mRNA	FALSCH	42,74	39,52
ITGB4	NM_001005731.1	Endogenou	mRNA	FALSCH	115,48	318,63
MYO1D	NM_015194.1	Endogenou	mRNA	FALSCH	263,7	165,75
ABI3BP	NM_015429.3	Endogenou	mRNA	FALSCH	234,4	351,83
RBL1	NM_183404.1	Endogenou	mRNA	FALSCH	31,37	11,72
NR3C1	NM_001018077.1	Endogenou	mRNA	FALSCH	378,23	204,59
PPP2R1A	NM_014225.3	Endogenou	mRNA	FALSCH	805,76	202,83
TNXB	NM_032470.3	Endogenou	mRNA	FALSCH	52,99	111,44
AP1M2	NM_005498.4	Endogenou	mRNA	FALSCH	159,95	77,46
NRXN1	NM_138735.2	Endogenou	mRNA	FALSCH	8,01	4,16
PYCARD	NM_013258.3	Endogenou	mRNA	FALSCH	33,61	26,66
CALCRL	NM_005795.3	Endogenou	mRNA	FALSCH	267,04	573,57
ITGA6	NM_000210.1	Endogenou	mRNA	FALSCH	253,51	89,97
PTX3	NM_002852.3	Endogenou	mRNA	FALSCH	13,61	14,21
SYK	NM_003177.3	Endogenou	mRNA	FALSCH	134,55	68,86
SPDEF	NM_012391.1	Endogenou	mRNA	FALSCH	658,7	1077,28
PROK2	NM_021935.3	Endogenou	mRNA	FALSCH	9,5	9,31
SYNE1	NM_015293.1	Endogenou	mRNA	FALSCH	83,68	115,32
EGFR	NM_201282.1	Endogenou	mRNA	FALSCH	414,08	372,57
ID2	NM_002166.4	Endogenou	mRNA	FALSCH	135,58	103,62
HOXB13	NM_006361.5	Endogenou	mRNA	FALSCH	336,52	691,37
CD36	NM_000072.3	Endogenou	mRNA	FALSCH	247,88	362,15
OAS1	NM_001032409.1	Endogenou	mRNA	FALSCH	178,98	812,59
PLXDC1	NM_020405.4	Endogenou	mRNA	FALSCH	51,39	42,06
GPATCH3	NM_022078.2	Housekeep	mRNA	FALSCH	36,2	12,81
IL13RA2	NM_000640.2	Endogenou	mRNA	FALSCH	10,04	30,69
NRXN3	NM_001105250.1	Endogenou	mRNA	FALSCH	39,71	96,09
MISP	NM_173481.2	Endogenou	mRNA	FALSCH	30,03	223,57
TLK2	NM_006852.2	Housekeep	mRNA	FALSCH	207,48	36,09
WNT5B	NM_032642.2	Endogenou	mRNA	FALSCH	37,52	27,68
ITGA2	NM_002203.2	Endogenou	mRNA	FALSCH	75,15	54,47
TNFRSF1A	NM_001065.2	Endogenou	mRNA	FALSCH	436,1	230,17
RBX1	NM_014248.2	Endogenou	mRNA	FALSCH	579,75	133,23
LTBP4	NM_003573.2	Endogenou	mRNA	FALSCH	417,98	632,62
RGCC	NM_014059.2	Endogenou	mRNA	FALSCH	1008,2	2223,71
COL18A1	NM_030582.3	Endogenou	mRNA	FALSCH	379,1	283,18
HSPB1	NM_001540.3	Endogenou	mRNA	FALSCH	654,7	468,32
PTK6	NM_005975.2	Endogenou	mRNA	FALSCH	41,56	71,03
ATPIF1	NM_178190.2	Endogenou	mRNA	FALSCH	635,32	232,27
HDAC3	NM_003883.2	Housekeep	mRNA	FALSCH	154,82	34,5
ITGB3	NM_000212.2	Endogenou	mRNA	FALSCH	19,66	215,61
CDH2	NM_001792.3	Endogenou	mRNA	FALSCH	18,25	78,22
ITGB1	NM_033666.2	Endogenou	mRNA	FALSCH	1421,62	1221,35
VAV3	NM_001079874.1	Endogenou	mRNA	FALSCH	30,82	52
ZC3H14	NM_001160103.1	Housekeep	mRNA	FALSCH	282,15	76,45

CTSK		NM_000396.2		Endogenou	mRNA	FALSCH	335,71	314,1
PROM1		NM_006017.1		Endogenou	mRNA	FALSCH	8,88	20,01
RTN4		NM_007008.2		Endogenou	mRNA	FALSCH	1163,11	534,89
GDF15		NM_004864.2		Endogenou	mRNA	FALSCH	247,82	325,61
FBP1		NM_000507.3		Endogenou	mRNA	FALSCH	567,94	352,65
SNAI1		NM_005985.2		Endogenou	mRNA	FALSCH	26,07	21,1
VWA2		NM_001272046.1		Endogenou	mRNA	FALSCH	33,59	27,33
ESRP1		NM_001034915.2		Endogenou	mRNA	FALSCH	467,3	282,23
PMP22		NM_000304.2		Endogenou	mRNA	FALSCH	179,37	190,38
MYLK		NM_053032.2		Endogenou	mRNA	FALSCH	584,77	526,84
ITGA11		NM_012211.3		Endogenou	mRNA	FALSCH	60,31	73,39
FGFR1		NM_015850.2		Endogenou	mRNA	FALSCH	188,85	124,73
CD46		NM_172350.1		Endogenou	mRNA	FALSCH	1374,41	349,08
RUNX1		NM_001754.4		Endogenou	mRNA	FALSCH	107,56	64,16
THY1		NM_006288.2		Endogenou	mRNA	FALSCH	137,1	197,67
RAMP2		NM_005854.2		Endogenou	mRNA	FALSCH	129,22	200,6
VWA1		NM_199121.2		Endogenou	mRNA	FALSCH	21,81	27,54
SLIT2		NM_004787.1		Endogenou	mRNA	FALSCH	115,44	162,62
SRF		NM_003131.2		Endogenou	mRNA	FALSCH	252,27	57,85
TJP2		NM_004817.2		Endogenou	mRNA	FALSCH	109,33	48,72
EPHA1		NM_005232.3		Endogenou	mRNA	FALSCH	48,14	30,58
CREBBP		NM_001079846.1		Endogenou	mRNA	FALSCH	225,91	45,63
XXXXX	XXXXX	XXXXX	XXXXX	XXXXX	XXXXX	XXXXX	XXXXX	XXXXX
XXXXX	XXXXX	XXXXX	XXXXX	XXXXX	XXXXX	XXXXX	XXXXX	XXXXX
XXXXX	XXXXX	XXXXX	XXXXX	XXXXX	XXXXX	XXXXX	XXXXX	XXXXX
XXXXX	XXXXX	XXXXX	XXXXX	XXXXX	XXXXX	XXXXX	XXXXX	XXXXX
XXXXX	XXXXX	XXXXX	XXXXX	XXXXX	XXXXX	XXXXX	XXXXX	XXXXX
XXXXX	XXXXX	XXXXX	XXXXX	XXXXX	XXXXX	XXXXX	XXXXX	XXXXX
XXXXX	XXXXX	XXXXX	XXXXX	XXXXX	XXXXX	XXXXX	XXXXX	XXXXX
XXXXX	XXXXX	XXXXX	XXXXX	XXXXX	XXXXX	XXXXX	XXXXX	XXXXX
XXXXX	XXXXX	XXXXX	XXXXX	XXXXX	XXXXX	XXXXX	XXXXX	XXXXX
XXXXX	XXXXX	XXXXX	XXXXX	XXXXX	XXXXX	XXXXX	XXXXX	XXXXX
XXXXX	XXXXX	XXXXX	XXXXX	XXXXX	XXXXX	XXXXX	XXXXX	XXXXX
XXXXX	XXXXX	XXXXX	XXXXX	XXXXX	XXXXX	XXXXX	XXXXX	XXXXX
XXXXX	XXXXX	XXXXX	XXXXX	XXXXX	XXXXX	XXXXX	XXXXX	XXXXX
XXXXX	XXXXX	XXXXX	XXXXX	XXXXX	XXXXX	XXXXX	XXXXX	XXXXX
XXXXX	XXXXX	XXXXX	XXXXX	XXXXX	XXXXX	XXXXX	XXXXX	XXXXX
XXXXX	XXXXX	XXXXX	XXXXX	XXXXX	XXXXX	XXXXX	XXXXX	XXXXX
XXXXX	XXXXX	XXXXX	XXXXX	XXXXX	XXXXX	XXXXX	XXXXX	XXXXX
XXXXX	XXXXX	XXXXX	XXXXX	XXXXX	XXXXX	XXXXX	XXXXX	XXXXX
XXXXX	XXXXX	XXXXX	XXXXX	XXXXX	XXXXX	XXXXX	XXXXX	XXXXX
XXXXX	XXXXX	XXXXX	XXXXX	XXXXX	XXXXX	XXXXX	XXXXX	XXXXX
XXXXX	XXXXX	XXXXX	XXXXX	XXXXX	XXXXX	XXXXX	XXXXX	XXXXX
XXXXX	XXXXX	XXXXX	XXXXX	XXXXX	XXXXX	XXXXX	XXXXX	XXXXX
XXXXX	XXXXX	XXXXX	XXXXX	XXXXX	XXXXX	XXXXX	XXXXX	XXXXX
XXXXX	XXXXX	XXXXX	XXXXX	XXXXX	XXXXX	XXXXX	XXXXX	XXXXX
XXXXX	XXXXX	XXXXX	XXXXX	XXXXX	XXXXX	XXXXX	XXXXX	XXXXX
XXXXX	XXXXX	XXXXX	XXXXX	XXXXX	XXXXX	XXXXX	XXXXX	XXXXX
XXXXX	XXXXX	XXXXX	XXXXX	XXXXX	XXXXX	XXXXX	XXXXX	XXXXX

XXXXX	XXXXX	XXXXX	XXXXX	XXXXX	XXXXX	XXXXX	XXXXX	XXXXX
XXXXX	XXXXX	XXXXX	XXXXX	XXXXX	XXXXX	XXXXX	XXXXX	XXXXX
XXXXX	XXXXX	XXXXX	XXXXX	XXXXX	XXXXX	XXXXX	XXXXX	XXXXX
XXXXX	XXXXX	XXXXX	XXXXX	XXXXX	XXXXX	XXXXX	XXXXX	XXXXX
XXXXX	XXXXX	XXXXX	XXXXX	XXXXX	XXXXX	XXXXX	XXXXX	XXXXX
XXXXX	XXXXX	XXXXX	XXXXX	XXXXX	XXXXX	XXXXX	XXXXX	XXXXX
XXXXX	XXXXX	XXXXX	XXXXX	XXXXX	XXXXX	XXXXX	XXXXX	XXXXX
XXXXX	XXXXX	XXXXX	XXXXX	XXXXX	XXXXX	XXXXX	XXXXX	XXXXX
XXXXX	XXXXX	XXXXX	XXXXX	XXXXX	XXXXX	XXXXX	XXXXX	XXXXX
XXXXX	XXXXX	XXXXX	XXXXX	XXXXX	XXXXX	XXXXX	XXXXX	XXXXX
XXXXX	XXXXX	XXXXX	XXXXX	XXXXX	XXXXX	XXXXX	XXXXX	XXXXX
XXXXX	XXXXX	XXXXX	XXXXX	XXXXX	XXXXX	XXXXX	XXXXX	XXXXX
XXXXX	XXXXX	XXXXX	XXXXX	XXXXX	XXXXX	XXXXX	XXXXX	XXXXX
XXXXX	XXXXX	XXXXX	XXXXX	XXXXX	XXXXX	XXXXX	XXXXX	XXXXX
XXXXX	XXXXX	XXXXX	XXXXX	XXXXX	XXXXX	XXXXX	XXXXX	XXXXX
XXXXX	XXXXX	XXXXX	XXXXX	XXXXX	XXXXX	XXXXX	XXXXX	XXXXX
XXXXX	XXXXX	XXXXX	XXXXX	XXXXX	XXXXX	XXXXX	XXXXX	XXXXX
XXXXX	XXXXX	XXXXX	XXXXX	XXXXX	XXXXX	XXXXX	XXXXX	XXXXX
XXXXX	XXXXX	XXXXX	XXXXX	XXXXX	XXXXX	XXXXX	XXXXX	XXXXX
XXXXX	XXXXX	XXXXX	XXXXX	XXXXX	XXXXX	XXXXX	XXXXX	XXXXX
XXXXX	XXXXX	XXXXX	XXXXX	XXXXX	XXXXX	XXXXX	XXXXX	XXXXX
XXXXX	XXXXX	XXXXX	XXXXX	XXXXX	XXXXX	XXXXX	XXXXX	XXXXX
XXXXX	XXXXX	XXXXX	XXXXX	XXXXX	XXXXX	XXXXX	XXXXX	XXXXX
XXXXX	XXXXX	XXXXX	XXXXX	XXXXX	XXXXX	XXXXX	XXXXX	XXXXX
XXXXX	XXXXX	XXXXX	XXXXX	XXXXX	XXXXX	XXXXX	XXXXX	XXXXX
XXXXX	XXXXX	XXXXX	XXXXX	XXXXX	XXXXX	XXXXX	XXXXX	XXXXX
XXXXX	XXXXX	XXXXX	XXXXX	XXXXX	XXXXX	XXXXX	XXXXX	XXXXX
XXXXX	XXXXX	XXXXX	XXXXX	XXXXX	XXXXX	XXXXX	XXXXX	XXXXX
XXXXX	XXXXX	XXXXX	XXXXX	XXXXX	XXXXX	XXXXX	XXXXX	XXXXX
XXXXX	XXXXX	XXXXX	XXXXX	XXXXX	XXXXX	XXXXX	XXXXX	XXXXX
XXXXX	XXXXX	XXXXX	XXXXX	XXXXX	XXXXX	XXXXX	XXXXX	XXXXX
XXXXX	XXXXX	XXXXX	XXXXX	XXXXX	XXXXX	XXXXX	XXXXX	XXXXX
XXXXX	XXXXX	XXXXX	XXXXX	XXXXX	XXXXX	XXXXX	XXXXX	XXXXX
XXXXX	XXXXX	XXXXX	XXXXX	XXXXX	XXXXX	XXXXX	XXXXX	XXXXX
XXXXX	XXXXX	XXXXX	XXXXX	XXXXX	XXXXX	XXXXX	XXXXX	XXXXX
XXXXX	XXXXX	XXXXX	XXXXX	XXXXX	XXXXX	XXXXX	XXXXX	XXXXX
XXXXX	XXXXX	XXXXX	XXXXX	XXXXX	XXXXX	XXXXX	XXXXX	XXXXX
XXXXX	XXXXX	XXXXX	XXXXX	XXXXX	XXXXX	XXXXX	XXXXX	XXXXX
XXXXX	XXXXX	XXXXX	XXXXX	XXXXX	XXXXX	XXXXX	XXXXX	XXXXX
XXXXX	XXXXX	XXXXX	XXXXX	XXXXX	XXXXX	XXXXX	XXXXX	XXXXX
XXXXX	XXXXX	XXXXX	XXXXX	XXXXX	XXXXX	XXXXX	XXXXX	XXXXX
XXXXX	XXXXX	XXXXX	XXXXX	XXXXX	XXXXX	XXXXX	XXXXX	XXXXX
XXXXX	XXXXX	XXXXX	XXXXX	XXXXX	XXXXX	XXXXX	XXXXX	XXXXX
XXXXX	XXXXX	XXXXX	XXXXX	XXXXX	XXXXX	XXXXX	XXXXX	XXXXX
XXXXX	XXXXX	XXXXX	XXXXX	XXXXX	XXXXX	XXXXX	XXXXX	XXXXX
XXXXX	XXXXX	XXXXX	XXXXX	XXXXX	XXXXX	XXXXX	XXXXX	XXXXX
XXXXX	XXXXX	XXXXX	XXXXX	XXXXX	XXXXX	XXXXX	XXXXX	XXXXX
XXXXX	XXXXX	XXXXX	XXXXX	XXXXX	XXXXX	XXXXX	XXXXX	XXXXX
XXXXX	XXXXX	XXXXX	XXXXX	XXXXX	XXXXX	XXXXX	XXXXX	XXXXX
XXXXX	XXXXX	XXXXX	XXXXX	XXXXX	XXXXX	XXXXX	XXXXX	XXXXX

XXXXX	XXXXX	XXXXX	XXXXX	XXXXX	XXXXX	XXXXX	XXXXX	XXXXX
XXXXX	XXXXX	XXXXX	XXXXX	XXXXX	XXXXX	XXXXX	XXXXX	XXXXX
XXXXX	XXXXX	XXXXX	XXXXX	XXXXX	XXXXX	XXXXX	XXXXX	XXXXX
XXXXX	XXXXX	XXXXX	XXXXX	XXXXX	XXXXX	XXXXX	XXXXX	XXXXX
XXXXX	XXXXX	XXXXX	XXXXX	XXXXX	XXXXX	XXXXX	XXXXX	XXXXX
XXXXX	XXXXX	XXXXX	XXXXX	XXXXX	XXXXX	XXXXX	XXXXX	XXXXX
XXXXX	XXXXX	XXXXX	XXXXX	XXXXX	XXXXX	XXXXX	XXXXX	XXXXX
XXXXX	XXXXX	XXXXX	XXXXX	XXXXX	XXXXX	XXXXX	XXXXX	XXXXX
XXXXX	XXXXX	XXXXX	XXXXX	XXXXX	XXXXX	XXXXX	XXXXX	XXXXX
XXXXX	XXXXX	XXXXX	XXXXX	XXXXX	XXXXX	XXXXX	XXXXX	XXXXX
XXXXX	XXXXX	XXXXX	XXXXX	XXXXX	XXXXX	XXXXX	XXXXX	XXXXX
XXXXX	XXXXX	XXXXX	XXXXX	XXXXX	XXXXX	XXXXX	XXXXX	XXXXX
XXXXX	XXXXX	XXXXX	XXXXX	XXXXX	XXXXX	XXXXX	XXXXX	XXXXX
XXXXX	XXXXX	XXXXX	XXXXX	XXXXX	XXXXX	XXXXX	XXXXX	XXXXX
XXXXX	XXXXX	XXXXX	XXXXX	XXXXX	XXXXX	XXXXX	XXXXX	XXXXX
XXXXX	XXXXX	XXXXX	XXXXX	XXXXX	XXXXX	XXXXX	XXXXX	XXXXX
XXXXX	XXXXX	XXXXX	XXXXX	XXXXX	XXXXX	XXXXX	XXXXX	XXXXX
XXXXX	XXXXX	XXXXX	XXXXX	XXXXX	XXXXX	XXXXX	XXXXX	XXXXX
XXXXX	XXXXX	XXXXX	XXXXX	XXXXX	XXXXX	XXXXX	XXXXX	XXXXX
XXXXX	XXXXX	XXXXX	XXXXX	XXXXX	XXXXX	XXXXX	XXXXX	XXXXX
XXXXX	XXXXX	XXXXX	XXXXX	XXXXX	XXXXX	XXXXX	XXXXX	XXXXX
XXXXX	XXXXX	XXXXX	XXXXX	XXXXX	XXXXX	XXXXX	XXXXX	XXXXX
XXXXX	XXXXX	XXXXX	XXXXX	XXXXX	XXXXX	XXXXX	XXXXX	XXXXX
XXXXX	XXXXX	XXXXX	XXXXX	XXXXX	XXXXX	XXXXX	XXXXX	XXXXX
XXXXX	XXXXX	XXXXX	XXXXX	XXXXX	XXXXX	XXXXX	XXXXX	XXXXX
XXXXX	XXXXX	XXXXX	XXXXX	XXXXX	XXXXX	XXXXX	XXXXX	XXXXX
XXXXX	XXXXX	XXXXX	XXXXX	XXXXX	XXXXX	XXXXX	XXXXX	XXXXX
XXXXX	XXXXX	XXXXX	XXXXX	XXXXX	XXXXX	XXXXX	XXXXX	XXXXX
XXXXX	XXXXX	XXXXX	XXXXX	XXXXX	XXXXX	XXXXX	XXXXX	XXXXX
XXXXX	XXXXX	XXXXX	XXXXX	XXXXX	XXXXX	XXXXX	XXXXX	XXXXX
XXXXX	XXXXX	XXXXX	XXXXX	XXXXX	XXXXX	XXXXX	XXXXX	XXXXX
XXXXX	XXXXX	XXXXX	XXXXX	XXXXX	XXXXX	XXXXX	XXXXX	XXXXX
XXXXX	XXXXX	XXXXX	XXXXX	XXXXX	XXXXX	XXXXX	XXXXX	XXXXX
XXXXX	XXXXX	XXXXX	XXXXX	XXXXX	XXXXX	XXXXX	XXXXX	XXXXX
XXXXX	XXXXX	XXXXX	XXXXX	XXXXX	XXXXX	XXXXX	XXXXX	XXXXX
XXXXX	XXXXX	XXXXX	XXXXX	XXXXX	XXXXX	XXXXX	XXXXX	XXXXX
XXXXX	XXXXX	XXXXX	XXXXX	XXXXX	XXXXX	XXXXX	XXXXX	XXXXX
XXXXX	XXXXX	XXXXX	XXXXX	XXXXX	XXXXX	XXXXX	XXXXX	XXXXX
XXXXX	XXXXX	XXXXX	XXXXX	XXXXX	XXXXX	XXXXX	XXXXX	XXXXX
XXXXX	XXXXX	XXXXX	XXXXX	XXXXX	XXXXX	XXXXX	XXXXX	XXXXX
XXXXX	XXXXX	XXXXX	XXXXX	XXXXX	XXXXX	XXXXX	XXXXX	XXXXX
XXXXX	XXXXX	XXXXX	XXXXX	XXXXX	XXXXX	XXXXX	XXXXX	XXXXX
XXXXX	XXXXX	XXXXX	XXXXX	XXXXX	XXXXX	XXXXX	XXXXX	XXXXX
XXXXX	XXXXX	XXXXX	XXXXX	XXXXX	XXXXX	XXXXX	XXXXX	XXXXX
XXXXX	XXXXX	XXXXX	XXXXX	XXXXX	XXXXX	XXXXX	XXXXX	XXXXX
XXXXX	XXXXX	XXXXX	XXXXX	XXXXX	XXXXX	XXXXX	XXXXX	XXXXX
XXXXX	XXXXX	XXXXX	XXXXX	XXXXX	XXXXX	XXXXX	XXXXX	XXXXX
XXXXX	XXXXX	XXXXX	XXXXX	XXXXX	XXXXX	XXXXX	XXXXX	XXXXX
XXXXX	XXXXX	XXXXX	XXXXX	XXXXX	XXXXX	XXXXX	XXXXX	XXXXX
XXXXX	XXXXX	XXXXX	XXXXX	XXXXX	XXXXX	XXXXX	XXXXX	XXXXX

XXXXX	XXXXX	XXXXX	XXXXX	XXXXX	XXXXX	XXXXX	XXXXX	XXXXX
XXXXX	XXXXX	XXXXX	XXXXX	XXXXX	XXXXX	XXXXX	XXXXX	XXXXX
XXXXX	XXXXX	XXXXX	XXXXX	XXXXX	XXXXX	XXXXX	XXXXX	XXXXX
XXXXX	XXXXX	XXXXX	XXXXX	XXXXX	XXXXX	XXXXX	XXXXX	XXXXX
XXXXX	XXXXX	XXXXX	XXXXX	XXXXX	XXXXX	XXXXX	XXXXX	XXXXX
XXXXX	XXXXX	XXXXX	XXXXX	XXXXX	XXXXX	XXXXX	XXXXX	XXXXX
XXXXX	XXXXX	XXXXX	XXXXX	XXXXX	XXXXX	XXXXX	XXXXX	XXXXX
XXXXX	XXXXX	XXXXX	XXXXX	XXXXX	XXXXX	XXXXX	XXXXX	XXXXX
XXXXX	XXXXX	XXXXX	XXXXX	XXXXX	XXXXX	XXXXX	XXXXX	XXXXX
XXXXX	XXXXX	XXXXX	XXXXX	XXXXX	XXXXX	XXXXX	XXXXX	XXXXX
XXXXX	XXXXX	XXXXX	XXXXX	XXXXX	XXXXX	XXXXX	XXXXX	XXXXX
XXXXX	XXXXX	XXXXX	XXXXX	XXXXX	XXXXX	XXXXX	XXXXX	XXXXX
XXXXX	XXXXX	XXXXX	XXXXX	XXXXX	XXXXX	XXXXX	XXXXX	XXXXX
XXXXX	XXXXX	XXXXX	XXXXX	XXXXX	XXXXX	XXXXX	XXXXX	XXXXX
XXXXX	XXXXX	XXXXX	XXXXX	XXXXX	XXXXX	XXXXX	XXXXX	XXXXX
XXXXX	XXXXX	XXXXX	XXXXX	XXXXX	XXXXX	XXXXX	XXXXX	XXXXX
XXXXX	XXXXX	XXXXX	XXXXX	XXXXX	XXXXX	XXXXX	XXXXX	XXXXX
XXXXX	XXXXX	XXXXX	XXXXX	XXXXX	XXXXX	XXXXX	XXXXX	XXXXX
XXXXX	XXXXX	XXXXX	XXXXX	XXXXX	XXXXX	XXXXX	XXXXX	XXXXX
XXXXX	XXXXX	XXXXX	XXXXX	XXXXX	XXXXX	XXXXX	XXXXX	XXXXX
XXXXX	XXXXX	XXXXX	XXXXX	XXXXX	XXXXX	XXXXX	XXXXX	XXXXX
XXXXX	XXXXX	XXXXX	XXXXX	XXXXX	XXXXX	XXXXX	XXXXX	XXXXX
XXXXX	XXXXX	XXXXX	XXXXX	XXXXX	XXXXX	XXXXX	XXXXX	XXXXX
XXXXX	XXXXX	XXXXX	XXXXX	XXXXX	XXXXX	XXXXX	XXXXX	XXXXX
XXXXX	XXXXX	XXXXX	XXXXX	XXXXX	XXXXX	XXXXX	XXXXX	XXXXX
XXXXX	XXXXX	XXXXX	XXXXX	XXXXX	XXXXX	XXXXX	XXXXX	XXXXX
XXXXX	XXXXX	XXXXX	XXXXX	XXXXX	XXXXX	XXXXX	XXXXX	XXXXX
XXXXX	XXXXX	XXXXX	XXXXX	XXXXX	XXXXX	XXXXX	XXXXX	XXXXX
XXXXX	XXXXX	XXXXX	XXXXX	XXXXX	XXXXX	XXXXX	XXXXX	XXXXX
XXXXX	XXXXX	XXXXX	XXXXX	XXXXX	XXXXX	XXXXX	XXXXX	XXXXX
XXXXX	XXXXX	XXXXX	XXXXX	XXXXX	XXXXX	XXXXX	XXXXX	XXXXX
XXXXX	XXXXX	XXXXX	XXXXX	XXXXX	XXXXX	XXXXX	XXXXX	XXXXX
XXXXX	XXXXX	XXXXX	XXXXX	XXXXX	XXXXX	XXXXX	XXXXX	XXXXX
XXXXX	XXXXX	XXXXX	XXXXX	XXXXX	XXXXX	XXXXX	XXXXX	XXXXX
XXXXX	XXXXX	XXXXX	XXXXX	XXXXX	XXXXX	XXXXX	XXXXX	XXXXX
XXXXX	XXXXX	XXXXX	XXXXX	XXXXX	XXXXX	XXXXX	XXXXX	XXXXX
XXXXX	XXXXX	XXXXX	XXXXX	XXXXX	XXXXX	XXXXX	XXXXX	XXXXX
XXXXX	XXXXX	XXXXX	XXXXX	XXXXX	XXXXX	XXXXX	XXXXX	XXXXX
XXXXX	XXXXX	XXXXX	XXXXX	XXXXX	XXXXX	XXXXX	XXXXX	XXXXX
XXXXX	XXXXX	XXXXX	XXXXX	XXXXX	XXXXX	XXXXX	XXXXX	XXXXX
XXXXX	XXXXX	XXXXX	XXXXX	XXXXX	XXXXX	XXXXX	XXXXX	XXXXX
XXXXX	XXXXX	XXXXX	XXXXX	XXXXX	XXXXX	XXXXX	XXXXX	XXXXX
XXXXX	XXXXX	XXXXX	XXXXX	XXXXX	XXXXX	XXXXX	XXXXX	XXXXX
XXXXX	XXXXX	XXXXX	XXXXX	XXXXX	XXXXX	XXXXX	XXXXX	XXXXX
XXXXX	XXXXX	XXXXX	XXXXX	XXXXX	XXXXX	XXXXX	XXXXX	XXXXX
XXXXX	XXXXX	XXXXX	XXXXX	XXXXX	XXXXX	XXXXX	XXXXX	XXXXX
XXXXX	XXXXX	XXXXX	XXXXX	XXXXX	XXXXX	XXXXX	XXXXX	XXXXX
XXXXX	XXXXX	XXXXX	XXXXX	XXXXX	XXXXX	XXXXX	XXXXX	XXXXX
XXXXX	XXXXX	XXXXX	XXXXX	XXXXX	XXXXX	XXXXX	XXXXX	XXXXX
XXXXX	XXXXX	XXXXX	XXXXX	XXXXX	XXXXX	XXXXX	XXXXX	XXXXX

XXXXX	XXXXX	XXXXX	XXXXX	XXXXX	XXXXX	XXXXX	XXXXX	XXXXX
XXXXX	XXXXX	XXXXX	XXXXX	XXXXX	XXXXX	XXXXX	XXXXX	XXXXX
XXXXX	XXXXX	XXXXX	XXXXX	XXXXX	XXXXX	XXXXX	XXXXX	XXXXX
XXXXX	XXXXX	XXXXX	XXXXX	XXXXX	XXXXX	XXXXX	XXXXX	XXXXX
XXXXX	XXXXX	XXXXX	XXXXX	XXXXX	XXXXX	XXXXX	XXXXX	XXXXX
XXXXX	XXXXX	XXXXX	XXXXX	XXXXX	XXXXX	XXXXX	XXXXX	XXXXX
XXXXX	XXXXX	XXXXX	XXXXX	XXXXX	XXXXX	XXXXX	XXXXX	XXXXX
XXXXX	XXXXX	XXXXX	XXXXX	XXXXX	XXXXX	XXXXX	XXXXX	XXXXX
XXXXX	XXXXX	XXXXX	XXXXX	XXXXX	XXXXX	XXXXX	XXXXX	XXXXX
XXXXX	XXXXX	XXXXX	XXXXX	XXXXX	XXXXX	XXXXX	XXXXX	XXXXX
XXXXX	XXXXX	XXXXX	XXXXX	XXXXX	XXXXX	XXXXX	XXXXX	XXXXX
XXXXX	XXXXX	XXXXX	XXXXX	XXXXX	XXXXX	XXXXX	XXXXX	XXXXX
XXXXX	XXXXX	XXXXX	XXXXX	XXXXX	XXXXX	XXXXX	XXXXX	XXXXX
XXXXX	XXXXX	XXXXX	XXXXX	XXXXX	XXXXX	XXXXX	XXXXX	XXXXX
XXXXX	XXXXX	XXXXX	XXXXX	XXXXX	XXXXX	XXXXX	XXXXX	XXXXX
XXXXX	XXXXX	XXXXX	XXXXX	XXXXX	XXXXX	XXXXX	XXXXX	XXXXX
XXXXX	XXXXX	XXXXX	XXXXX	XXXXX	XXXXX	XXXXX	XXXXX	XXXXX
XXXXX	XXXXX	XXXXX	XXXXX	XXXXX	XXXXX	XXXXX	XXXXX	XXXXX
XXXXX	XXXXX	XXXXX	XXXXX	XXXXX	XXXXX	XXXXX	XXXXX	XXXXX
XXXXX	XXXXX	XXXXX	XXXXX	XXXXX	XXXXX	XXXXX	XXXXX	XXXXX
XXXXX	XXXXX	XXXXX	XXXXX	XXXXX	XXXXX	XXXXX	XXXXX	XXXXX
XXXXX	XXXXX	XXXXX	XXXXX	XXXXX	XXXXX	XXXXX	XXXXX	XXXXX
XXXXX	XXXXX	XXXXX	XXXXX	XXXXX	XXXXX	XXXXX	XXXXX	XXXXX
XXXXX	XXXXX	XXXXX	XXXXX	XXXXX	XXXXX	XXXXX	XXXXX	XXXXX
XXXXX	XXXXX	XXXXX	XXXXX	XXXXX	XXXXX	XXXXX	XXXXX	XXXXX
XXXXX	XXXXX	XXXXX	XXXXX	XXXXX	XXXXX	XXXXX	XXXXX	XXXXX
XXXXX	XXXXX	XXXXX	XXXXX	XXXXX	XXXXX	XXXXX	XXXXX	XXXXX
XXXXX	XXXXX	XXXXX	XXXXX	XXXXX	XXXXX	XXXXX	XXXXX	XXXXX
XXXXX	XXXXX	XXXXX	XXXXX	XXXXX	XXXXX	XXXXX	XXXXX	XXXXX
XXXXX	XXXXX	XXXXX	XXXXX	XXXXX	XXXXX	XXXXX	XXXXX	XXXXX
XXXXX	XXXXX	XXXXX	XXXXX	XXXXX	XXXXX	XXXXX	XXXXX	XXXXX
XXXXX	XXXXX	XXXXX	XXXXX	XXXXX	XXXXX	XXXXX	XXXXX	XXXXX
XXXXX	XXXXX	XXXXX	XXXXX	XXXXX	XXXXX	XXXXX	XXXXX	XXXXX
XXXXX	XXXXX	XXXXX	XXXXX	XXXXX	XXXXX	XXXXX	XXXXX	XXXXX
XXXXX	XXXXX	XXXXX	XXXXX	XXXXX	XXXXX	XXXXX	XXXXX	XXXXX
XXXXX	XXXXX	XXXXX	XXXXX	XXXXX	XXXXX	XXXXX	XXXXX	XXXXX
XXXXX	XXXXX	XXXXX	XXXXX	XXXXX	XXXXX	XXXXX	XXXXX	XXXXX
XXXXX	XXXXX	XXXXX	XXXXX	XXXXX	XXXXX	XXXXX	XXXXX	XXXXX
XXXXX	XXXXX	XXXXX	XXXXX	XXXXX	XXXXX	XXXXX	XXXXX	XXXXX
XXXXX	XXXXX	XXXXX	XXXXX	XXXXX	XXXXX	XXXXX	XXXXX	XXXXX
XXXXX	XXXXX	XXXXX	XXXXX	XXXXX	XXXXX	XXXXX	XXXXX	XXXXX
XXXXX	XXXXX	XXXXX	XXXXX	XXXXX	XXXXX	XXXXX	XXXXX	XXXXX
XXXXX	XXXXX	XXXXX	XXXXX	XXXXX	XXXXX	XXXXX	XXXXX	XXXXX
XXXXX	XXXXX	XXXXX	XXXXX	XXXXX	XXXXX	XXXXX	XXXXX	XXXXX
XXXXX	XXXXX	XXXXX	XXXXX	XXXXX	XXXXX	XXXXX	XXXXX	XXXXX
XXXXX	XXXXX	XXXXX	XXXXX	XXXXX	XXXXX	XXXXX	XXXXX	XXXXX
XXXXX	XXXXX	XXXXX	XXXXX	XXXXX	XXXXX	XXXXX	XXXXX	XXXXX
XXXXX	XXXXX	XXXXX	XXXXX	XXXXX	XXXXX	XXXXX	XXXXX	XXXXX
XXXXX	XXXXX	XXXXX	XXXXX	XXXXX	XXXXX	XXXXX	XXXXX	XXXXX
XXXXX	XXXXX	XXXXX	XXXXX	XXXXX	XXXXX	XXXXX	XXXXX	XXXXX

XXXXX	XXXXX	XXXXX	XXXXX	XXXXX	XXXXX	XXXXX	XXXXX	XXXXX
XXXXX	XXXXX	XXXXX	XXXXX	XXXXX	XXXXX	XXXXX	XXXXX	XXXXX
XXXXX	XXXXX	XXXXX	XXXXX	XXXXX	XXXXX	XXXXX	XXXXX	XXXXX
XXXXX	XXXXX	XXXXX	XXXXX	XXXXX	XXXXX	XXXXX	XXXXX	XXXXX
XXXXX	XXXXX	XXXXX	XXXXX	XXXXX	XXXXX	XXXXX	XXXXX	XXXXX
XXXXX	XXXXX	XXXXX	XXXXX	XXXXX	XXXXX	XXXXX	XXXXX	XXXXX
XXXXX	XXXXX	XXXXX	XXXXX	XXXXX	XXXXX	XXXXX	XXXXX	XXXXX
XXXXX	XXXXX	XXXXX	XXXXX	XXXXX	XXXXX	XXXXX	XXXXX	XXXXX
XXXXX	XXXXX	XXXXX	XXXXX	XXXXX	XXXXX	XXXXX	XXXXX	XXXXX
XXXXX	XXXXX	XXXXX	XXXXX	XXXXX	XXXXX	XXXXX	XXXXX	XXXXX
XXXXX	XXXXX	XXXXX	XXXXX	XXXXX	XXXXX	XXXXX	XXXXX	XXXXX
XXXXX	XXXXX	XXXXX	XXXXX	XXXXX	XXXXX	XXXXX	XXXXX	XXXXX
XXXXX	XXXXX	XXXXX	XXXXX	XXXXX	XXXXX	XXXXX	XXXXX	XXXXX
XXXXX	XXXXX	XXXXX	XXXXX	XXXXX	XXXXX	XXXXX	XXXXX	XXXXX
XXXXX	XXXXX	XXXXX	XXXXX	XXXXX	XXXXX	XXXXX	XXXXX	XXXXX
XXXXX	XXXXX	XXXXX	XXXXX	XXXXX	XXXXX	XXXXX	XXXXX	XXXXX
XXXXX	XXXXX	XXXXX	XXXXX	XXXXX	XXXXX	XXXXX	XXXXX	XXXXX
XXXXX	XXXXX	XXXXX	XXXXX	XXXXX	XXXXX	XXXXX	XXXXX	XXXXX
XXXXX	XXXXX	XXXXX	XXXXX	XXXXX	XXXXX	XXXXX	XXXXX	XXXXX
XXXXX	XXXXX	XXXXX	XXXXX	XXXXX	XXXXX	XXXXX	XXXXX	XXXXX
XXXXX	XXXXX	XXXXX	XXXXX	XXXXX	XXXXX	XXXXX	XXXXX	XXXXX
XXXXX	XXXXX	XXXXX	XXXXX	XXXXX	XXXXX	XXXXX	XXXXX	XXXXX
XXXXX	XXXXX	XXXXX	XXXXX	XXXXX	XXXXX	XXXXX	XXXXX	XXXXX
XXXXX	XXXXX	XXXXX	XXXXX	XXXXX	XXXXX	XXXXX	XXXXX	XXXXX
XXXXX	XXXXX	XXXXX	XXXXX	XXXXX	XXXXX	XXXXX	XXXXX	XXXXX
XXXXX	XXXXX	XXXXX	XXXXX	XXXXX	XXXXX	XXXXX	XXXXX	XXXXX
XXXXX	XXXXX	XXXXX	XXXXX	XXXXX	XXXXX	XXXXX	XXXXX	XXXXX
XXXXX	XXXXX	XXXXX	XXXXX	XXXXX	XXXXX	XXXXX	XXXXX	XXXXX
XXXXX	XXXXX	XXXXX	XXXXX	XXXXX	XXXXX	XXXXX	XXXXX	XXXXX
XXXXX	XXXXX	XXXXX	XXXXX	XXXXX	XXXXX	XXXXX	XXXXX	XXXXX
XXXXX	XXXXX	XXXXX	XXXXX	XXXXX	XXXXX	XXXXX	XXXXX	XXXXX
XXXXX	XXXXX	XXXXX	XXXXX	XXXXX	XXXXX	XXXXX	XXXXX	XXXXX
XXXXX	XXXXX	XXXXX	XXXXX	XXXXX	XXXXX	XXXXX	XXXXX	XXXXX
XXXXX	XXXXX	XXXXX	XXXXX	XXXXX	XXXXX	XXXXX	XXXXX	XXXXX
XXXXX	XXXXX	XXXXX	XXXXX	XXXXX	XXXXX	XXXXX	XXXXX	XXXXX
XXXXX	XXXXX	XXXXX	XXXXX	XXXXX	XXXXX	XXXXX	XXXXX	XXXXX
XXXXX	XXXXX	XXXXX	XXXXX	XXXXX	XXXXX	XXXXX	XXXXX	XXXXX
XXXXX	XXXXX	XXXXX	XXXXX	XXXXX	XXXXX	XXXXX	XXXXX	XXXXX
XXXXX	XXXXX	XXXXX	XXXXX	XXXXX	XXXXX	XXXXX	XXXXX	XXXXX
XXXXX	XXXXX	XXXXX	XXXXX	XXXXX	XXXXX	XXXXX	XXXXX	XXXXX
XXXXX	XXXXX	XXXXX	XXXXX	XXXXX	XXXXX	XXXXX	XXXXX	XXXXX
XXXXX	XXXXX	XXXXX	XXXXX	XXXXX	XXXXX	XXXXX	XXXXX	XXXXX
XXXXX	XXXXX	XXXXX	XXXXX	XXXXX	XXXXX	XXXXX	XXXXX	XXXXX
XXXXX	XXXXX	XXXXX	XXXXX	XXXXX	XXXXX	XXXXX	XXXXX	XXXXX
XXXXX	XXXXX	XXXXX	XXXXX	XXXXX	XXXXX	XXXXX	XXXXX	XXXXX
XXXXX	XXXXX	XXXXX	XXXXX	XXXXX	XXXXX	XXXXX	XXXXX	XXXXX
XXXXX	XXXXX	XXXXX	XXXXX	XXXXX	XXXXX	XXXXX	XXXXX	XXXXX
XXXXX	XXXXX	XXXXX	XXXXX	XXXXX	XXXXX	XXXXX	XXXXX	XXXXX
XXXXX	XXXXX	XXXXX	XXXXX	XXXXX	XXXXX	XXXXX	XXXXX	XXXXX
XXXXX	XXXXX	XXXXX	XXXXX	XXXXX	XXXXX	XXXXX	XXXXX	XXXXX

XXXXX	XXXXX	XXXXX	XXXXX	XXXXX	XXXXX	XXXXX	XXXXX	XXXXX
XXXXX	XXXXX	XXXXX	XXXXX	XXXXX	XXXXX	XXXXX	XXXXX	XXXXX
XXXXX	XXXXX	XXXXX	XXXXX	XXXXX	XXXXX	XXXXX	XXXXX	XXXXX
XXXXX	XXXXX	XXXXX	XXXXX	XXXXX	XXXXX	XXXXX	XXXXX	XXXXX
XXXXX	XXXXX	XXXXX	XXXXX	XXXXX	XXXXX	XXXXX	XXXXX	XXXXX
XXXXX	XXXXX	XXXXX	XXXXX	XXXXX	XXXXX	XXXXX	XXXXX	XXXXX
XXXXX	XXXXX	XXXXX	XXXXX	XXXXX	XXXXX	XXXXX	XXXXX	XXXXX
XXXXX	XXXXX	XXXXX	XXXXX	XXXXX	XXXXX	XXXXX	XXXXX	XXXXX
XXXXX	XXXXX	XXXXX	XXXXX	XXXXX	XXXXX	XXXXX	XXXXX	XXXXX
XXXXX	XXXXX	XXXXX	XXXXX	XXXXX	XXXXX	XXXXX	XXXXX	XXXXX
XXXXX	XXXXX	XXXXX	XXXXX	XXXXX	XXXXX	XXXXX	XXXXX	XXXXX
XXXXX	XXXXX	XXXXX	XXXXX	XXXXX	XXXXX	XXXXX	XXXXX	XXXXX
XXXXX	XXXXX	XXXXX	XXXXX	XXXXX	XXXXX	XXXXX	XXXXX	XXXXX
XXXXX	XXXXX	XXXXX	XXXXX	XXXXX	XXXXX	XXXXX	XXXXX	XXXXX
XXXXX	XXXXX	XXXXX	XXXXX	XXXXX	XXXXX	XXXXX	XXXXX	XXXXX
XXXXX	XXXXX	XXXXX	XXXXX	XXXXX	XXXXX	XXXXX	XXXXX	XXXXX
XXXXX	XXXXX	XXXXX	XXXXX	XXXXX	XXXXX	XXXXX	XXXXX	XXXXX
XXXXX	XXXXX	XXXXX	XXXXX	XXXXX	XXXXX	XXXXX	XXXXX	XXXXX
XXXXX	XXXXX	XXXXX	XXXXX	XXXXX	XXXXX	XXXXX	XXXXX	XXXXX
XXXXX	XXXXX	XXXXX	XXXXX	XXXXX	XXXXX	XXXXX	XXXXX	XXXXX
XXXXX	XXXXX	XXXXX	XXXXX	XXXXX	XXXXX	XXXXX	XXXXX	XXXXX
XXXXX	XXXXX	XXXXX	XXXXX	XXXXX	XXXXX	XXXXX	XXXXX	XXXXX
XXXXX	XXXXX	XXXXX	XXXXX	XXXXX	XXXXX	XXXXX	XXXXX	XXXXX
XXXXX	XXXXX	XXXXX	XXXXX	XXXXX	XXXXX	XXXXX	XXXXX	XXXXX
XXXXX	XXXXX	XXXXX	XXXXX	XXXXX	XXXXX	XXXXX	XXXXX	XXXXX
XXXXX	XXXXX	XXXXX	XXXXX	XXXXX	XXXXX	XXXXX	XXXXX	XXXXX
XXXXX	XXXXX	XXXXX	XXXXX	XXXXX	XXXXX	XXXXX	XXXXX	XXXXX
XXXXX	XXXXX	XXXXX	XXXXX	XXXXX	XXXXX	XXXXX	XXXXX	XXXXX
XXXXX	XXXXX	XXXXX	XXXXX	XXXXX	XXXXX	XXXXX	XXXXX	XXXXX
XXXXX	XXXXX	XXXXX	XXXXX	XXXXX	XXXXX	XXXXX	XXXXX	XXXXX
XXXXX	XXXXX	XXXXX	XXXXX	XXXXX	XXXXX	XXXXX	XXXXX	XXXXX
XXXXX	XXXXX	XXXXX	XXXXX	XXXXX	XXXXX	XXXXX	XXXXX	XXXXX
XXXXX	XXXXX	XXXXX	XXXXX	XXXXX	XXXXX	XXXXX	XXXXX	XXXXX
XXXXX	XXXXX	XXXXX	XXXXX	XXXXX	XXXXX	XXXXX	XXXXX	XXXXX
XXXXX	XXXXX	XXXXX	XXXXX	XXXXX	XXXXX	XXXXX	XXXXX	XXXXX
XXXXX	XXXXX	XXXXX	XXXXX	XXXXX	XXXXX	XXXXX	XXXXX	XXXXX
XXXXX	XXXXX	XXXXX	XXXXX	XXXXX	XXXXX	XXXXX	XXXXX	XXXXX
XXXXX	XXXXX	XXXXX	XXXXX	XXXXX	XXXXX	XXXXX	XXXXX	XXXXX
XXXXX	XXXXX	XXXXX	XXXXX	XXXXX	XXXXX	XXXXX	XXXXX	XXXXX
XXXXX	XXXXX	XXXXX	XXXXX	XXXXX	XXXXX	XXXXX	XXXXX	XXXXX
XXXXX	XXXXX	XXXXX	XXXXX	XXXXX	XXXXX	XXXXX	XXXXX	XXXXX
XXXXX	XXXXX	XXXXX	XXXXX	XXXXX	XXXXX	XXXXX	XXXXX	XXXXX
XXXXX	XXXXX	XXXXX	XXXXX	XXXXX	XXXXX	XXXXX	XXXXX	XXXXX
XXXXX	XXXXX	XXXXX	XXXXX	XXXXX	XXXXX	XXXXX	XXXXX	XXXXX
XXXXX	XXXXX	XXXXX	XXXXX	XXXXX	XXXXX	XXXXX	XXXXX	XXXXX
XXXXX	XXXXX	XXXXX	XXXXX	XXXXX	XXXXX	XXXXX	XXXXX	XXXXX
XXXXX	XXXXX	XXXXX	XXXXX	XXXXX	XXXXX	XXXXX	XXXXX	XXXXX
XXXXX	XXXXX	XXXXX	XXXXX	XXXXX	XXXXX	XXXXX	XXXXX	XXXXX
XXXXX	XXXXX	XXXXX	XXXXX	XXXXX	XXXXX	XXXXX	XXXXX	XXXXX
XXXXX	XXXXX	XXXXX	XXXXX	XXXXX	XXXXX	XXXXX	XXXXX	XXXXX

XXXXX	XXXXX	XXXXX	XXXXX	XXXXX	XXXXX	XXXXX	XXXXX	XXXXX
XXXXX	XXXXX	XXXXX	XXXXX	XXXXX	XXXXX	XXXXX	XXXXX	XXXXX
XXXXX	XXXXX	XXXXX	XXXXX	XXXXX	XXXXX	XXXXX	XXXXX	XXXXX
XXXXX	XXXXX	XXXXX	XXXXX	XXXXX	XXXXX	XXXXX	XXXXX	XXXXX
XXXXX	XXXXX	XXXXX	XXXXX	XXXXX	XXXXX	XXXXX	XXXXX	XXXXX
XXXXX	XXXXX	XXXXX	XXXXX	XXXXX	XXXXX	XXXXX	XXXXX	XXXXX
XXXXX	XXXXX	XXXXX	XXXXX	XXXXX	XXXXX	XXXXX	XXXXX	XXXXX
XXXXX	XXXXX	XXXXX	XXXXX	XXXXX	XXXXX	XXXXX	XXXXX	XXXXX
XXXXX	XXXXX	XXXXX	XXXXX	XXXXX	XXXXX	XXXXX	XXXXX	XXXXX
XXXXX	XXXXX	XXXXX	XXXXX	XXXXX	XXXXX	XXXXX	XXXXX	XXXXX
XXXXX	XXXXX	XXXXX	XXXXX	XXXXX	XXXXX	XXXXX	XXXXX	XXXXX
XXXXX	XXXXX	XXXXX	XXXXX	XXXXX	XXXXX	XXXXX	XXXXX	XXXXX
XXXXX	XXXXX	XXXXX	XXXXX	XXXXX	XXXXX	XXXXX	XXXXX	XXXXX
XXXXX	XXXXX	XXXXX	XXXXX	XXXXX	XXXXX	XXXXX	XXXXX	XXXXX
XXXXX	XXXXX	XXXXX	XXXXX	XXXXX	XXXXX	XXXXX	XXXXX	XXXXX
XXXXX	XXXXX	XXXXX	XXXXX	XXXXX	XXXXX	XXXXX	XXXXX	XXXXX
XXXXX	XXXXX	XXXXX	XXXXX	XXXXX	XXXXX	XXXXX	XXXXX	XXXXX
XXXXX	XXXXX	XXXXX	XXXXX	XXXXX	XXXXX	XXXXX	XXXXX	XXXXX
XXXXX	XXXXX	XXXXX	XXXXX	XXXXX	XXXXX	XXXXX	XXXXX	XXXXX
XXXXX	XXXXX	XXXXX	XXXXX	XXXXX	XXXXX	XXXXX	XXXXX	XXXXX
XXXXX	XXXXX	XXXXX	XXXXX	XXXXX	XXXXX	XXXXX	XXXXX	XXXXX
XXXXX	XXXXX	XXXXX	XXXXX	XXXXX	XXXXX	XXXXX	XXXXX	XXXXX
XXXXX	XXXXX	XXXXX	XXXXX	XXXXX	XXXXX	XXXXX	XXXXX	XXXXX
XXXXX	XXXXX	XXXXX	XXXXX	XXXXX	XXXXX	XXXXX	XXXXX	XXXXX
XXXXX	XXXXX	XXXXX	XXXXX	XXXXX	XXXXX	XXXXX	XXXXX	XXXXX
XXXXX	XXXXX	XXXXX	XXXXX	XXXXX	XXXXX	XXXXX	XXXXX	XXXXX
XXXXX	XXXXX	XXXXX	XXXXX	XXXXX	XXXXX	XXXXX	XXXXX	XXXXX
XXXXX	XXXXX	XXXXX	XXXXX	XXXXX	XXXXX	XXXXX	XXXXX	XXXXX
XXXXX	XXXXX	XXXXX	XXXXX	XXXXX	XXXXX	XXXXX	XXXXX	XXXXX
XXXXX	XXXXX	XXXXX	XXXXX	XXXXX	XXXXX	XXXXX	XXXXX	XXXXX
XXXXX	XXXXX	XXXXX	XXXXX	XXXXX	XXXXX	XXXXX	XXXXX	XXXXX
XXXXX	XXXXX	XXXXX	XXXXX	XXXXX	XXXXX	XXXXX	XXXXX	XXXXX
XXXXX	XXXXX	XXXXX	XXXXX	XXXXX	XXXXX	XXXXX	XXXXX	XXXXX
XXXXX	XXXXX	XXXXX	XXXXX	XXXXX	XXXXX	XXXXX	XXXXX	XXXXX
XXXXX	XXXXX	XXXXX	XXXXX	XXXXX	XXXXX	XXXXX	XXXXX	XXXXX
XXXXX	XXXXX	XXXXX	XXXXX	XXXXX	XXXXX	XXXXX	XXXXX	XXXXX
XXXXX	XXXXX	XXXXX	XXXXX	XXXXX	XXXXX	XXXXX	XXXXX	XXXXX
XXXXX	XXXXX	XXXXX	XXXXX	XXXXX	XXXXX	XXXXX	XXXXX	XXXXX
XXXXX	XXXXX	XXXXX	XXXXX	XXXXX	XXXXX	XXXXX	XXXXX	XXXXX
XXXXX	XXXXX	XXXXX	XXXXX	XXXXX	XXXXX	XXXXX	XXXXX	XXXXX
XXXXX	XXXXX	XXXXX	XXXXX	XXXXX	XXXXX	XXXXX	XXXXX	XXXXX
XXXXX	XXXXX	XXXXX	XXXXX	XXXXX	XXXXX	XXXXX	XXXXX	XXXXX
XXXXX	XXXXX	XXXXX	XXXXX	XXXXX	XXXXX	XXXXX	XXXXX	XXXXX
XXXXX	XXXXX	XXXXX	XXXXX	XXXXX	XXXXX	XXXXX	XXXXX	XXXXX
XXXXX	XXXXX	XXXXX	XXXXX	XXXXX	XXXXX	XXXXX	XXXXX	XXXXX
XXXXX	XXXXX	XXXXX	XXXXX	XXXXX	XXXXX	XXXXX	XXXXX	XXXXX
XXXXX	XXXXX	XXXXX	XXXXX	XXXXX	XXXXX	XXXXX	XXXXX	XXXXX
XXXXX	XXXXX	XXXXX	XXXXX	XXXXX	XXXXX	XXXXX	XXXXX	XXXXX
XXXXX	XXXXX	XXXXX	XXXXX	XXXXX	XXXXX	XXXXX	XXXXX	XXXXX
XXXXX	XXXXX	XXXXX	XXXXX	XXXXX	XXXXX	XXXXX	XXXXX	XXXXX

XXXXX	XXXXX	XXXXX	XXXXX	XXXXX	XXXXX	XXXXX	XXXXX	XXXXX
XXXXX	XXXXX	XXXXX	XXXXX	XXXXX	XXXXX	XXXXX	XXXXX	XXXXX
XXXXX	XXXXX	XXXXX	XXXXX	XXXXX	XXXXX	XXXXX	XXXXX	XXXXX
XXXXX	XXXXX	XXXXX	XXXXX	XXXXX	XXXXX	XXXXX	XXXXX	XXXXX
XXXXX	XXXXX	XXXXX	XXXXX	XXXXX	XXXXX	XXXXX	XXXXX	XXXXX
XXXXX	XXXXX	XXXXX	XXXXX	XXXXX	XXXXX	XXXXX	XXXXX	XXXXX
XXXXX	XXXXX	XXXXX	XXXXX	XXXXX	XXXXX	XXXXX	XXXXX	XXXXX
XXXXX	XXXXX	XXXXX	XXXXX	XXXXX	XXXXX	XXXXX	XXXXX	XXXXX
XXXXX	XXXXX	XXXXX	XXXXX	XXXXX	XXXXX	XXXXX	XXXXX	XXXXX
XXXXX	XXXXX	XXXXX	XXXXX	XXXXX	XXXXX	XXXXX	XXXXX	XXXXX
XXXXX	XXXXX	XXXXX	XXXXX	XXXXX	XXXXX	XXXXX	XXXXX	XXXXX
XXXXX	XXXXX	XXXXX	XXXXX	XXXXX	XXXXX	XXXXX	XXXXX	XXXXX
XXXXX	XXXXX	XXXXX	XXXXX	XXXXX	XXXXX	XXXXX	XXXXX	XXXXX
XXXXX	XXXXX	XXXXX	XXXXX	XXXXX	XXXXX	XXXXX	XXXXX	XXXXX
XXXXX	XXXXX	XXXXX	XXXXX	XXXXX	XXXXX	XXXXX	XXXXX	XXXXX
XXXXX	XXXXX	XXXXX	XXXXX	XXXXX	XXXXX	XXXXX	XXXXX	XXXXX
XXXXX	XXXXX	XXXXX	XXXXX	XXXXX	XXXXX	XXXXX	XXXXX	XXXXX
XXXXX	XXXXX	XXXXX	XXXXX	XXXXX	XXXXX	XXXXX	XXXXX	XXXXX
XXXXX	XXXXX	XXXXX	XXXXX	XXXXX	XXXXX	XXXXX	XXXXX	XXXXX
XXXXX	XXXXX	XXXXX	XXXXX	XXXXX	XXXXX	XXXXX	XXXXX	XXXXX
XXXXX	XXXXX	XXXXX	XXXXX	XXXXX	XXXXX	XXXXX	XXXXX	XXXXX
XXXXX	XXXXX	XXXXX	XXXXX	XXXXX	XXXXX	XXXXX	XXXXX	XXXXX
XXXXX	XXXXX	XXXXX	XXXXX	XXXXX	XXXXX	XXXXX	XXXXX	XXXXX
XXXXX	XXXXX	XXXXX	XXXXX	XXXXX	XXXXX	XXXXX	XXXXX	XXXXX
XXXXX	XXXXX	XXXXX	XXXXX	XXXXX	XXXXX	XXXXX	XXXXX	XXXXX
XXXXX	XXXXX	XXXXX	XXXXX	XXXXX	XXXXX	XXXXX	XXXXX	XXXXX
XXXXX	XXXXX	XXXXX	XXXXX	XXXXX	XXXXX	XXXXX	XXXXX	XXXXX
XXXXX	XXXXX	XXXXX	XXXXX	XXXXX	XXXXX	XXXXX	XXXXX	XXXXX
XXXXX	XXXXX	XXXXX	XXXXX	XXXXX	XXXXX	XXXXX	XXXXX	XXXXX
XXXXX	XXXXX	XXXXX	XXXXX	XXXXX	XXXXX	XXXXX	XXXXX	XXXXX
XXXXX	XXXXX	XXXXX	XXXXX	XXXXX	XXXXX	XXXXX	XXXXX	XXXXX
XXXXX	XXXXX	XXXXX	XXXXX	XXXXX	XXXXX	XXXXX	XXXXX	XXXXX
XXXXX	XXXXX	XXXXX	XXXXX	XXXXX	XXXXX	XXXXX	XXXXX	XXXXX
XXXXX	XXXXX	XXXXX	XXXXX	XXXXX	XXXXX	XXXXX	XXXXX	XXXXX
XXXXX	XXXXX	XXXXX	XXXXX	XXXXX	XXXXX	XXXXX	XXXXX	XXXXX
XXXXX	XXXXX	XXXXX	XXXXX	XXXXX	XXXXX	XXXXX	XXXXX	XXXXX
XXXXX	XXXXX	XXXXX	XXXXX	XXXXX	XXXXX	XXXXX	XXXXX	XXXXX
XXXXX	XXXXX	XXXXX	XXXXX	XXXXX	XXXXX	XXXXX	XXXXX	XXXXX
XXXXX	XXXXX	XXXXX	XXXXX	XXXXX	XXXXX	XXXXX	XXXXX	XXXXX
XXXXX	XXXXX	XXXXX	XXXXX	XXXXX	XXXXX	XXXXX	XXXXX	XXXXX
XXXXX	XXXXX	XXXXX	XXXXX	XXXXX	XXXXX	XXXXX	XXXXX	XXXXX
XXXXX	XXXXX	XXXXX	XXXXX	XXXXX	XXXXX	XXXXX	XXXXX	XXXXX
XXXXX	XXXXX	XXXXX	XXXXX	XXXXX	XXXXX	XXXXX	XXXXX	XXXXX
XXXXX	XXXXX	XXXXX	XXXXX	XXXXX	XXXXX	XXXXX	XXXXX	XXXXX
XXXXX	XXXXX	XXXXX	XXXXX	XXXXX	XXXXX	XXXXX	XXXXX	XXXXX
XXXXX	XXXXX	XXXXX	XXXXX	XXXXX	XXXXX	XXXXX	XXXXX	XXXXX
XXXXX	XXXXX	XXXXX	XXXXX	XXXXX	XXXXX	XXXXX	XXXXX	XXXXX
XXXXX	XXXXX	XXXXX	XXXXX	XXXXX	XXXXX	XXXXX	XXXXX	XXXXX
XXXXX	XXXXX	XXXXX	XXXXX	XXXXX	XXXXX	XXXXX	XXXXX	XXXXX
XXXXX	XXXXX	XXXXX	XXXXX	XXXXX	XXXXX	XXXXX	XXXXX	XXXXX

XXXXX	XXXXX	XXXXX	XXXXX	XXXXX	XXXXX	XXXXX	XXXXX	XXXXX
XXXXX	XXXXX	XXXXX	XXXXX	XXXXX	XXXXX	XXXXX	XXXXX	XXXXX
XXXXX	XXXXX	XXXXX	XXXXX	XXXXX	XXXXX	XXXXX	XXXXX	XXXXX
XXXXX	XXXXX	XXXXX	XXXXX	XXXXX	XXXXX	XXXXX	XXXXX	XXXXX
XXXXX	XXXXX	XXXXX	XXXXX	XXXXX	XXXXX	XXXXX	XXXXX	XXXXX
XXXXX	XXXXX	XXXXX	XXXXX	XXXXX	XXXXX	XXXXX	XXXXX	XXXXX
XXXXX	XXXXX	XXXXX	XXXXX	XXXXX	XXXXX	XXXXX	XXXXX	XXXXX
XXXXX	XXXXX	XXXXX	XXXXX	XXXXX	XXXXX	XXXXX	XXXXX	XXXXX
XXXXX	XXXXX	XXXXX	XXXXX	XXXXX	XXXXX	XXXXX	XXXXX	XXXXX
XXXXX	XXXXX	XXXXX	XXXXX	XXXXX	XXXXX	XXXXX	XXXXX	XXXXX
XXXXX	XXXXX	XXXXX	XXXXX	XXXXX	XXXXX	XXXXX	XXXXX	XXXXX
XXXXX	XXXXX	XXXXX	XXXXX	XXXXX	XXXXX	XXXXX	XXXXX	XXXXX
XXXXX	XXXXX	XXXXX	XXXXX	XXXXX	XXXXX	XXXXX	XXXXX	XXXXX
XXXXX	XXXXX	XXXXX	XXXXX	XXXXX	XXXXX	XXXXX	XXXXX	XXXXX
XXXXX	XXXXX	XXXXX	XXXXX	XXXXX	XXXXX	XXXXX	XXXXX	XXXXX
XXXXX	XXXXX	XXXXX	XXXXX	XXXXX	XXXXX	XXXXX	XXXXX	XXXXX
XXXXX	XXXXX	XXXXX	XXXXX	XXXXX	XXXXX	XXXXX	XXXXX	XXXXX
XXXXX	XXXXX	XXXXX	XXXXX	XXXXX	XXXXX	XXXXX	XXXXX	XXXXX
XXXXX	XXXXX	XXXXX	XXXXX	XXXXX	XXXXX	XXXXX	XXXXX	XXXXX
XXXXX	XXXXX	XXXXX	XXXXX	XXXXX	XXXXX	XXXXX	XXXXX	XXXXX
XXXXX	XXXXX	XXXXX	XXXXX	XXXXX	XXXXX	XXXXX	XXXXX	XXXXX
XXXXX	XXXXX	XXXXX	XXXXX	XXXXX	XXXXX	XXXXX	XXXXX	XXXXX
XXXXX	XXXXX	XXXXX	XXXXX	XXXXX	XXXXX	XXXXX	XXXXX	XXXXX
XXXXX	XXXXX	XXXXX	XXXXX	XXXXX	XXXXX	XXXXX	XXXXX	XXXXX
XXXXX	XXXXX	XXXXX	XXXXX	XXXXX	XXXXX	XXXXX	XXXXX	XXXXX
XXXXX	XXXXX	XXXXX	XXXXX	XXXXX	XXXXX	XXXXX	XXXXX	XXXXX
XXXXX	XXXXX	XXXXX	XXXXX	XXXXX	XXXXX	XXXXX	XXXXX	XXXXX
XXXXX	XXXXX	XXXXX	XXXXX	XXXXX	XXXXX	XXXXX	XXXXX	XXXXX
XXXXX	XXXXX	XXXXX	XXXXX	XXXXX	XXXXX	XXXXX	XXXXX	XXXXX
XXXXX	XXXXX	XXXXX	XXXXX	XXXXX	XXXXX	XXXXX	XXXXX	XXXXX
XXXXX	XXXXX	XXXXX	XXXXX	XXXXX	XXXXX	XXXXX	XXXXX	XXXXX
XXXXX	XXXXX	XXXXX	XXXXX	XXXXX	XXXXX	XXXXX	XXXXX	XXXXX
XXXXX	XXXXX	XXXXX	XXXXX	XXXXX	XXXXX	XXXXX	XXXXX	XXXXX
XXXXX	XXXXX	XXXXX	XXXXX	XXXXX	XXXXX	XXXXX	XXXXX	XXXXX

Reference	StDev of Re	LM vs. Reference	P value of: LM vs.	**PvaluLM/PLC**	Lower 95%	Upper 95%
35,57	56,8	-6,74	0,00000001	WAHR	0,1	-4,34
196,08	133,65	5,25	0,00000001	WAHR	3,39	8,13
16,06	22,83	6,16	0,00000001	WAHR	3,72	10,22
6,02	7,82	5,65	0,00000005	WAHR	3,38	9,45
29,71	40,81	6,06	0,00000023	WAHR	3,44	10,68
6,37	28,76	14,42	0,0000002	WAHR	6,42	32,4
21,48	31,69	15,79	0,00000017	WAHR	7,2	34,66
27,78	67,71	7,71	0,00000171	WAHR	3,77	15,78
27,42	80,55	20,12	0,00000143	WAHR	7,34	55,14
12,02	14,81	4,2	0,00000163	WAHR	2,53	6,97
20,85	20,94	4,59	0,00000271	WAHR	2,63	8,02
18,77	32,32	5,48	0,00000353	WAHR	2,95	10,18
266,23	122,99	-1,81	0,00000491	WAHR	0,44	-1,45
12,25	16,03	3,88	0,00000829	WAHR	2,29	6,58
380,94	488,94	2,78	0,00000788	WAHR	1,87	4,14
7,16	11,23	4,09	0,00000893	WAHR	2,35	7,12
3,91	3,82	4,63	0,0000095	WAHR	2,58	8,3
4,37	7,48	3,56	0,00000704	WAHR	2,2	5,78
141,99	262,67	-6,27	0,00000777	WAHR	0,08	-3,12
236,43	89,4	-1,78	0,00001079	WAHR	0,45	-1,42
3,35	4,25	9,03	0,00001146	WAHR	4,01	20,33
101,84	48,83	1,89	0,00001752	WAHR	1,46	2,46
1414,34	1054,95	-1,89	0,00001713	WAHR	0,41	-1,46
333,72	136,57	-1,67	0,0000171	WAHR	0,49	-1,35
112,18	1861,86	-19,57	0,00001659	WAHR	0,02	-6,28
180,08	83,71	-2	0,00001545	WAHR	0,38	-1,52
3,84	4,37	6,46	0,00001625	WAHR	3,17	13,15
177,43	2624,01	-21,35	0,00001969	WAHR	0,01	-6,28
7,4	12,69	5,05	0,00001449	WAHR	2,64	9,65
2,56	2,2	2,55	0,00001529	WAHR	1,75	3,73
4,81	6,94	8,39	0,00002302	WAHR	3,62	19,44
4,39	5	2,9	0,0000265	WAHR	1,85	4,55
3,32	3,04	9,38	0,00003168	WAHR	3,92	22,44
258,94	208,53	-3,32	0,00003311	WAHR	0,19	-2,04
28,89	46,44	3,16	0,00003139	WAHR	1,94	5,15
28,56	38,99	3,05	0,00003559	WAHR	1,89	4,93
110,84	47,46	-1,86	0,00003742	WAHR	0,41	-1,42
18,91	32,18	6,08	0,00004622	WAHR	2,78	13,27
391,79	129,54	-1,44	0,00005068	WAHR	0,59	-1,23
21,06	44,57	6,22	0,00005545	WAHR	2,77	13,98
6,81	10,7	3,34	0,00006071	WAHR	1,95	5,71
115,75	134,69	3,12	0,00005985	WAHR	1,89	5,16
53,23	44,25	2,6	0,00006305	WAHR	1,7	3,97
3,85	2,8	3,02	0,00007607	WAHR	1,85	4,93
15,08	19,72	4,51	0,00007475	WAHR	2,29	8,87
90,77	50,89	2,82	0,00007073	WAHR	1,8	4,42
313,76	87,75	1,41	0,00007436	WAHR	1,21	1,66

1287,45	619,98	-1,86	0,0000735	WAHR	0,41	-1,41
10,6	11,66	3,06	0,00008321	WAHR	1,84	5,11
7,92	11,05	3,26	0,00008929	WAHR	1,89	5,6
9,17	28,51	10,96	0,00008764	WAHR	3,77	31,83
228,04	58,18	1,5	0,00009227	WAHR	1,25	1,81
3,72	4,65	2,93	0,00009761	WAHR	1,78	4,82
638,58	315,25	-1,5	0,00009978	WAHR	0,55	-1,24
8,29	102,12	9,24	0,00010581	WAHR	3,36	25,41
11,75	33	5,75	0,00011156	WAHR	2,55	12,98
304,39	184,92	-1,68	0,00011439	WAHR	0,47	-1,32
5,57	10,85	10,09	0,00011087	WAHR	3,65	27,85
31,87	43,49	-2,49	0,00013854	WAHR	0,26	-1,63
19,73	39,33	2,88	0,00015188	WAHR	1,73	4,78
151,89	63,14	2,32	0,00014977	WAHR	1,58	3,41
150,57	63,85	1,85	0,00015916	WAHR	1,38	2,47
71,55	113,22	6,25	0,0001582	WAHR	2,66	14,67
25,67	19	2,31	0,00016831	WAHR	1,55	3,44
1004,37	845,18	-1,82	0,00018216	WAHR	0,41	-1,36
190,15	71,05	1,61	0,00018505	WAHR	1,28	2,02
6,86	7,34	9,7	0,00018965	WAHR	3,45	27,23
16,98	18,93	2,92	0,00022028	WAHR	1,72	4,96
41,97	534,07	7,76	0,00022563	WAHR	2,82	21,38
370,32	145,04	-1,59	0,00024944	WAHR	0,5	-1,26
17,92	42,62	-3,3	0,00024873	WAHR	0,17	-1,84
127,11	62,31	-1,47	0,00024832	WAHR	0,56	-1,21
23,35	27,37	3,07	0,00026232	WAHR	1,76	5,36
12	14,19	3,96	0,00026614	WAHR	2,01	7,81
738,43	1079,6	-4,5	0,00029465	WAHR	0,11	-2,17
1397,17	428,27	-1,42	0,0002915	WAHR	0,59	-1,19
2192,79	869,39	-1,61	0,00028897	WAHR	0,49	-1,26
2,7	2,26	3,53	0,00031332	WAHR	1,91	6,54
234,71	299,69	3,12	0,00031907	WAHR	1,76	5,52
22,72	20,39	3	0,00032344	WAHR	1,72	5,21
207,95	87,16	1,63	0,00032925	WAHR	1,27	2,1
106,34	49,95	-2,31	0,00033733	WAHR	0,29	-1,53
16,98	38,12	8,94	0,00039775	WAHR	2,98	26,82
7,53	17,28	3,5	0,00039398	WAHR	1,83	6,7
77,42	216,77	-4,85	0,00042834	WAHR	0,09	-2,13
633,19	192,43	1,38	0,00046592	WAHR	1,17	1,64
80,73	77,87	2,07	0,00047903	WAHR	1,42	3,04
49,78	59,26	2,63	0,00051408	WAHR	1,57	4,38
7,55	17,42	5,91	0,0005225	WAHR	2,32	15,03
18420,2	77878,02	-5,57	0,00055053	WAHR	0,07	-2,22
188,24	59,96	1,36	0,00056803	WAHR	1,15	1,6
685,53	507,3	-1,81	0,0006058	WAHR	0,4	-1,32
341,28	145,68	-1,44	0,00062299	WAHR	0,57	-1,18
37,87	38,62	3,33	0,00063028	WAHR	1,74	6,39
8,36	18,27	6,15	0,00063878	WAHR	2,35	16,13

12,52	16,57	-2,25	0,00068601	WAHR	0,29	-1,45
33,4	48,3	2,7	0,00065933	WAHR	1,57	4,64
52,5	78,44	2,84	0,00067862	WAHR	1,62	4,98
15,53	21,88	3,02	0,00068601	WAHR	1,65	5,52
79,26	46,58	3,39	0,00067295	WAHR	1,8	6,39
38,28	45,55	2,57	0,00073629	WAHR	1,53	4,33
12,84	19,41	3,83	0,00072969	WAHR	1,84	7,95
101,54	115,08	2,39	0,00074591	WAHR	1,48	3,87
368,48	167,2	-1,43	0,00079878	WAHR	0,58	-1,17
136,38	46,14	1,43	0,00080865	WAHR	1,17	1,74
2,76	2,52	1,82	0,00086114	WAHR	1,3	2,54
41,08	37,34	3,22	0,00094063	WAHR	1,69	6,12
4,12	7,82	2,75	0,00097912	WAHR	1,56	4,86
326,58	709,04	2,5	0,00099046	WAHR	1,49	4,2
115,85	111	2,4	0,00100899	WAHR	1,47	3,92
76,06	42,35	4,21	0,00105959	WAHR	1,94	9,16
2,93	2,1	1,94	0,00107701	WAHR	1,33	2,83
18,97	24,95	-2,48	0,001136	WAHR	0,24	-1,47
1799,99	1739,37	-2,14	0,00112784	WAHR	0,3	-1,38
41,6	21,71	1,76	0,00120756	WAHR	1,27	2,42
78,85	72,29	3,03	0,00120135	WAHR	1,62	5,65
522,49	194,66	-1,35	0,00123666	WAHR	0,62	-1,14
1089,13	915,49	-2,38	0,00130102	WAHR	0,26	-1,45
200,57	252,93	-2,48	0,00132177	WAHR	0,24	-1,46
30,93	500,84	4,7	0,00135687	WAHR	1,91	11,6
30,7	33,02	2,17	0,0014834	WAHR	1,38	3,43
384,8	153,42	1,62	0,00154397	WAHR	1,22	2,14
1042,53	522,54	1,75	0,00158462	WAHR	1,26	2,43
302,26	397,21	2,53	0,00163612	WAHR	1,46	4,39
6,62	8,52	2,84	0,00168263	WAHR	1,53	5,28
7,75	34,31	4,33	0,00173661	WAHR	1,8	10,41
8,92	14,07	4,86	0,00184889	WAHR	1,91	12,36
127,27	388,52	3,91	0,00189282	WAHR	1,72	8,88
47,15	26,58	-1,68	0,00192914	WAHR	0,43	-1,23
202,27	78,96	1,46	0,00191885	WAHR	1,16	1,83
49,08	109,53	4,76	0,00188991	WAHR	1,88	12,01
8,27	6,85	3,5	0,00197852	WAHR	1,67	7,36
67,67	814,84	5,26	0,00199663	WAHR	1,91	14,48
3,59	10,76	2,12	0,00204631	WAHR	1,34	3,35
197,05	236,48	-2,54	0,00203571	WAHR	0,22	-1,44
22,11	242,08	3,3	0,00216287	WAHR	1,59	6,86
187,79	150,14	2,57	0,0021553	WAHR	1,46	4,51
253,84	72,8	-1,3	0,00230199	WAHR	0,65	-1,11
13,62	19,06	3,4	0,00241043	WAHR	1,61	7,19
28,89	26,64	3,32	0,00239768	WAHR	1,6	6,88
74,34	39,51	1,78	0,00239121	WAHR	1,25	2,53
47,3	46,34	2,1	0,00237911	WAHR	1,33	3,31
223,06	109,39	2,26	0,00259778	WAHR	1,38	3,72

4965,81	18502,88	-3,62	0,00263667	WAHR	0,12	-1,61
134,76	66,55	-1,83	0,0026355	WAHR	0,37	-1,26
462,07	1905,24	-5,22	0,00271044	WAHR	0,07	-1,84
78,93	57,78	1,82	0,00276641	WAHR	1,25	2,65
6,55	17,82	4	0,00284184	WAHR	1,68	9,53
218,02	80	-1,4	0,00286438	WAHR	0,57	-1,13
27,82	60,83	3,03	0,00292496	WAHR	1,5	6,11
94,21	33,79	1,73	0,00297603	WAHR	1,23	2,44
62,27	47,64	3	0,00317744	WAHR	1,49	6,04
4,65	4,84	2,19	0,00327078	WAHR	1,32	3,62
169,35	37,94	1,22	0,00330505	WAHR	1,07	1,38
24,62	22,18	1,88	0,00333202	WAHR	1,25	2,81
123,78	120,72	2,68	0,00336345	WAHR	1,42	5,04
170	95,39	2,99	0,00345452	WAHR	1,5	5,95
81,53	153,16	2,93	0,00353704	WAHR	1,47	5,84
271,41	224,19	2,64	0,00362636	WAHR	1,42	4,91
23,69	13,13	2,04	0,00369207	WAHR	1,28	3,26
158,04	259,57	-2,34	0,00368439	WAHR	0,25	-1,35
110,94	185,48	2,31	0,00378107	WAHR	1,34	4
51,9	34,08	1,96	0,00375888	WAHR	1,27	3,02
5954,15	14151,77	-2,55	0,00390213	WAHR	0,21	-1,38
509,37	1042,93	-2,55	0,00404359	WAHR	0,21	-1,37
8,64	28,21	3,95	0,0042117	WAHR	1,59	9,78
179,45	84,34	1,5	0,00429227	WAHR	1,15	1,97
15,33	44,48	3,38	0,00447578	WAHR	1,5	7,61
45,19	27,21	2,55	0,00465935	WAHR	1,38	4,72
7,29	12,38	2,34	0,00476859	WAHR	1,32	4,16
50,04	24,55	2,22	0,00490562	WAHR	1,31	3,75
31,31	40,49	1,95	0,00512669	WAHR	1,24	3,07
109,62	48,21	-1,33	0,00519174	WAHR	0,62	-1,1
138,15	79,52	1,55	0,00525842	WAHR	1,15	2,09
45,73	154,08	-2,76	0,00613292	WAHR	0,18	-1,37
133,37	94,7	-1,47	0,00626655	WAHR	0,52	-1,12
627,87	843,19	-3,1	0,00648039	WAHR	0,15	-1,41
1285,52	1600,93	-1,82	0,00659916	WAHR	0,36	-1,19
123,63	180,54	2,08	0,00687859	WAHR	1,24	3,48
2221,89	1815,21	-1,8	0,00687294	WAHR	0,37	-1,19
293,95	115,98	1,35	0,00701029	WAHR	1,09	1,66
16,14	12,67	2,64	0,00748111	WAHR	1,32	5,26
57,43	34,94	1,78	0,00747922	WAHR	1,18	2,69
523,5	264,75	-1,56	0,00778883	WAHR	0,46	-1,13
144,09	51,42	1,32	0,00773311	WAHR	1,08	1,62
896,75	989,46	2,01	0,00778072	WAHR	1,22	3,33
127,68	74,96	-1,32	0,00815052	WAHR	0,62	-1,08
3,45	7,65	2,74	0,00833289	WAHR	1,32	5,67
30,33	39,63	2,62	0,00845174	WAHR	1,3	5,26
149,39	74,42	1,75	0,0085268	WAHR	1,17	2,61
56,5	162,74	3,29	0,00878967	WAHR	1,37	7,86

177,03	127,69	-1,93	0,00908918	WAHR	0,32	-1,19
65,07	373,43	-3,9	0,00904712	WAHR	0,09	-1,43
142,06	55,47	-1,26	0,00914987	WAHR	0,67	-1,06
34,94	53	2,61	0,00944882	WAHR	1,28	5,29
73,41	61,35	1,76	0,00990462	WAHR	1,16	2,69
102,1	72,37	2,04	0,00988299	WAHR	1,2	3,45
135,96	57,97	2,01	0,01007577	WAHR	1,2	3,34
7,6	13,78	2,45	0,01035644	WAHR	1,25	4,78
291,85	192,63	2,12	0,01053399	WAHR	1,21	3,7
31,73	28,5	2,42	0,01075668	WAHR	1,25	4,7
29,66	12,49	1,47	0,01086972	WAHR	1,1	1,98
217,61	51,24	-1,21	0,01149878	WAHR	0,72	-1,05
141,31	113,99	1,86	0,01165482	WAHR	1,16	2,98
30,85	25,21	1,85	0,01176079	WAHR	1,16	2,95
616,24	277,47	-1,53	0,01183609	WAHR	0,47	-1,11
1264,45	917,97	-1,68	0,01193108	WAHR	0,4	-1,13
7,58	16,87	2,61	0,0121692	WAHR	1,25	5,46
317,72	317,06	2,02	0,0120827	WAHR	1,18	3,46
213,79	56,33	-1,26	0,0121654	WAHR	0,66	-1,06
15,91	30,19	2,12	0,01243402	WAHR	1,19	3,78
265,5	88,55	1,28	0,0127084	WAHR	1,06	1,54
23,03	230,87	-3,73	0,01268877	WAHR	0,1	-1,35
143,98	49,3	-1,22	0,01288651	WAHR	0,71	-1,05
5,91	20,51	2,31	0,01319002	WAHR	1,2	4,44
147,84	97,79	1,75	0,013268	WAHR	1,13	2,7
20,74	21,01	2,15	0,01359553	WAHR	1,18	3,91
249,68	86,45	-1,26	0,01368265	WAHR	0,67	-1,05
36,94	57,7	2,29	0,01377942	WAHR	1,2	4,37
7,04	9,48	1,8	0,0140468	WAHR	1,13	2,86
10,1	17,87	2,05	0,01465715	WAHR	1,16	3,61
13,96	14,89	2,04	0,01504523	WAHR	1,16	3,6
199,62	68,09	1,23	0,0149844	WAHR	1,04	1,44
14,89	47,61	3,33	0,01515376	WAHR	1,28	8,65
112,26	35,46	-1,28	0,01552628	WAHR	0,64	-1,05
222,76	129,48	1,91	0,01559667	WAHR	1,14	3,19
27,44	32,04	1,96	0,01550341	WAHR	1,15	3,36
862,9	599,92	-2,17	0,01544661	WAHR	0,25	-1,18
48,86	52,72	1,83	0,01532029	WAHR	1,13	2,96
31,79	17,62	1,48	0,01540885	WAHR	1,08	2,03
124,84	79,68	1,52	0,01575926	WAHR	1,09	2,12
428,24	877,91	-1,72	0,01634568	WAHR	0,38	-1,11
9217,43	11047,52	-2,41	0,01649239	WAHR	0,2	-1,19
877,08	796,58	-1,9	0,01689872	WAHR	0,31	-1,13
39,81	26,42	1,7	0,01714719	WAHR	1,11	2,62
64,73	62,7	-2,1	0,01817961	WAHR	0,26	-1,15
1346,95	962,02	-1,54	0,01813547	WAHR	0,45	-1,08
560,95	1137,26	-2,24	0,01841687	WAHR	0,23	-1,15
217,45	117,2	1,56	0,01873433	WAHR	1,08	2,24

3,08	5,3	1,51	0,01897174	WAHR	1,08	2,13
6,3	12,86	2,48	0,01909978	WAHR	1,17	5,25
157,67	70,97	1,37	0,02089378	WAHR	1,05	1,79
22,75	20,18	1,88	0,02106715	WAHR	1,11	3,19
52,64	87,89	2,19	0,02119277	WAHR	1,14	4,24
191,81	71,28	1,37	0,02139768	WAHR	1,05	1,8
100	113,24	2,34	0,02131487	WAHR	1,15	4,8
17,32	21,94	1,81	0,0215733	WAHR	1,1	2,98
234,17	164,26	1,62	0,02179933	WAHR	1,08	2,42
1007,27	493,74	-1,25	0,02268993	WAHR	0,66	-1,03
21,95	28,65	2,41	0,02278117	WAHR	1,14	5,1
243,8	84,37	-1,52	0,02293127	WAHR	0,46	-1,07
18,42	142,04	-2,3	0,02265873	WAHR	0,21	-1,13
19,21	17,11	1,75	0,02320918	WAHR	1,08	2,82
120,15	108,89	2,22	0,02343571	WAHR	1,13	4,39
181,89	110,07	1,39	0,02400099	WAHR	1,05	1,86
37,7	211,64	-2,77	0,02475357	WAHR	0,15	-1,15
93,95	46,57	1,43	0,02471234	WAHR	1,05	1,95
1567,25	826,18	-2,38	0,02493035	WAHR	0,2	-1,13
5,4	7,62	1,76	0,02470525	WAHR	1,08	2,87
43,48	38,64	1,92	0,02553035	WAHR	1,09	3,4
283,59	115,33	1,46	0,02545249	WAHR	1,05	2,03
79,08	86,9	1,71	0,02521717	WAHR	1,07	2,74
1043,04	407,12	-3,1	0,02541159	WAHR	0,12	-1,17
101,18	311,36	2,45	0,02540668	WAHR	1,12	5,34
81,79	367,25	2,19	0,02601505	WAHR	1,1	4,34
89,61	103,63	-1,74	0,02611882	WAHR	0,35	-1,07
47,31	19,23	-1,31	0,02651687	WAHR	0,61	-1,03
4,71	9,37	2,13	0,02672515	WAHR	1,1	4,13
16,43	71,99	2,42	0,02734188	WAHR	1,11	5,27
10,72	29,42	2,8	0,02975565	WAHR	1,11	7,05
242,31	59,83	-1,17	0,02969705	WAHR	0,74	-1,02
69,99	75,11	-1,87	0,03037304	WAHR	0,31	-1,06
40,99	35,23	1,83	0,03030314	WAHR	1,06	3,16
297,57	179,22	1,47	0,03101091	WAHR	1,04	2,07
712,23	311,78	-1,23	0,03090131	WAHR	0,68	-1,02
214,98	147,06	1,94	0,03206585	WAHR	1,06	3,55
421,27	888,83	2,39	0,03230598	WAHR	1,08	5,29
603,4	517,92	-1,59	0,03353905	WAHR	0,41	-1,04
1210,99	2644,18	-1,85	0,0339539	WAHR	0,31	-1,05
86,5	184,9	-2,08	0,033925	WAHR	0,24	-1,06
483,87	226,02	1,31	0,03392278	WAHR	1,02	1,69
179,9	37,21	-1,16	0,03500174	WAHR	0,75	-1,01
44,76	74,03	-2,28	0,03554021	WAHR	0,2	-1,06
46,72	283,37	-2,56	0,03610744	WAHR	0,16	-1,07
1011,52	367,41	1,41	0,03634498	WAHR	1,02	1,93
17,64	18,78	1,75	0,03652391	WAHR	1,04	2,94
374,24	309,14	-1,33	0,03701863	WAHR	0,58	-1,02

643,17	1772,85		-1,92	0,03738761	WAHR	0,28	-1,04
4,92	6,37		1,81	0,03754776	WAHR	1,04	3,15
833,41	691,28		1,4	0,03807002	WAHR	1,02	1,91
525,29	1250,75		-2,12	0,03881227	WAHR	0,23	-1,04
281,96	426,28		2,01	0,03858518	WAHR	1,04	3,9
44,08	167,68		-1,69	0,03873032	WAHR	0,36	-1,03
69,5	115,05		-2,07	0,03953303	WAHR	0,24	-1,04
774,26	395,44		-1,66	0,03989902	WAHR	0,37	-1,03
99,93	76,48		1,79	0,04006634	WAHR	1,03	3,13
329,12	508,33		1,78	0,04034493	WAHR	1,03	3,08
108,81	108,6		-1,8	0,04110704	WAHR	0,32	-1,03
316,83	446,99		-1,68	0,04218927	WAHR	0,36	-1,02
1106,3	464,11		1,24	0,04493528	WAHR	1,01	1,54
62,75	74,51		1,71	0,04546805	WAHR	1,01	2,91
253,75	276,61		-1,85	0,04597051	WAHR	0,3	-1,01
74,06	66,25		1,74	0,04571933	WAHR	1,01	3,01
35,91	46,83		-1,65	0,04588304	WAHR	0,37	-1,01
62,58	56,09		1,84	0,04632892	WAHR	1,01	3,37
327	261		-1,3	0,04654668	WAHR	0,6	-1
78,51	32,6		1,39	0,04707251	WAHR	1	1,93
30,2	21,8		1,59	0,04868198	WAHR	1	2,54
280,19	108,86		-1,24	0,04954078	WAHR	0,65	-1
XXXXX	XXXXX	XXXXX		XXXXX	XXXXX	XXXXX	XXXXX
XXXXX	XXXXX	XXXXX		XXXXX	XXXXX	XXXXX	XXXXX
XXXXX	XXXXX	XXXXX		XXXXX	XXXXX	XXXXX	XXXXX
XXXXX	XXXXX	XXXXX		XXXXX	XXXXX	XXXXX	XXXXX
XXXXX	XXXXX	XXXXX		XXXXX	XXXXX	XXXXX	XXXXX
XXXXX	XXXXX	XXXXX		XXXXX	XXXXX	XXXXX	XXXXX
XXXXX	XXXXX	XXXXX		XXXXX	XXXXX	XXXXX	XXXXX
XXXXX	XXXXX	XXXXX		XXXXX	XXXXX	XXXXX	XXXXX
XXXXX	XXXXX	XXXXX		XXXXX	XXXXX	XXXXX	XXXXX
XXXXX	XXXXX	XXXXX		XXXXX	XXXXX	XXXXX	XXXXX
XXXXX	XXXXX	XXXXX		XXXXX	XXXXX	XXXXX	XXXXX
XXXXX	XXXXX	XXXXX		XXXXX	XXXXX	XXXXX	XXXXX
XXXXX	XXXXX	XXXXX		XXXXX	XXXXX	XXXXX	XXXXX
XXXXX	XXXXX	XXXXX		XXXXX	XXXXX	XXXXX	XXXXX
XXXXX	XXXXX	XXXXX		XXXXX	XXXXX	XXXXX	XXXXX
XXXXX	XXXXX	XXXXX		XXXXX	XXXXX	XXXXX	XXXXX
XXXXX	XXXXX	XXXXX		XXXXX	XXXXX	XXXXX	XXXXX
XXXXX	XXXXX	XXXXX		XXXXX	XXXXX	XXXXX	XXXXX
XXXXX	XXXXX	XXXXX		XXXXX	XXXXX	XXXXX	XXXXX
XXXXX	XXXXX	XXXXX		XXXXX	XXXXX	XXXXX	XXXXX
XXXXX	XXXXX	XXXXX		XXXXX	XXXXX	XXXXX	XXXXX
XXXXX	XXXXX	XXXXX		XXXXX	XXXXX	XXXXX	XXXXX
XXXXX	XXXXX	XXXXX		XXXXX	XXXXX	XXXXX	XXXXX
XXXXX	XXXXX	XXXXX		XXXXX	XXXXX	XXXXX	XXXXX
XXXXX	XXXXX	XXXXX		XXXXX	XXXXX	XXXXX	XXXXX
XXXXX	XXXXX	XXXXX		XXXXX	XXXXX	XXXXX	XXXXX
XXXXX	XXXXX	XXXXX		XXXXX	XXXXX	XXXXX	XXXXX

XXXXX	XXXXX	XXXXX	XXXXX	XXXXX	XXXXX	XXXXX
XXXXX	XXXXX	XXXXX	XXXXX	XXXXX	XXXXX	XXXXX
XXXXX	XXXXX	XXXXX	XXXXX	XXXXX	XXXXX	XXXXX
XXXXX	XXXXX	XXXXX	XXXXX	XXXXX	XXXXX	XXXXX
XXXXX	XXXXX	XXXXX	XXXXX	XXXXX	XXXXX	XXXXX
XXXXX	XXXXX	XXXXX	XXXXX	XXXXX	XXXXX	XXXXX
XXXXX	XXXXX	XXXXX	XXXXX	XXXXX	XXXXX	XXXXX
XXXXX	XXXXX	XXXXX	XXXXX	XXXXX	XXXXX	XXXXX
XXXXX	XXXXX	XXXXX	XXXXX	XXXXX	XXXXX	XXXXX
XXXXX	XXXXX	XXXXX	XXXXX	XXXXX	XXXXX	XXXXX
XXXXX	XXXXX	XXXXX	XXXXX	XXXXX	XXXXX	XXXXX
XXXXX	XXXXX	XXXXX	XXXXX	XXXXX	XXXXX	XXXXX
XXXXX	XXXXX	XXXXX	XXXXX	XXXXX	XXXXX	XXXXX
XXXXX	XXXXX	XXXXX	XXXXX	XXXXX	XXXXX	XXXXX
XXXXX	XXXXX	XXXXX	XXXXX	XXXXX	XXXXX	XXXXX
XXXXX	XXXXX	XXXXX	XXXXX	XXXXX	XXXXX	XXXXX
XXXXX	XXXXX	XXXXX	XXXXX	XXXXX	XXXXX	XXXXX
XXXXX	XXXXX	XXXXX	XXXXX	XXXXX	XXXXX	XXXXX
XXXXX	XXXXX	XXXXX	XXXXX	XXXXX	XXXXX	XXXXX
XXXXX	XXXXX	XXXXX	XXXXX	XXXXX	XXXXX	XXXXX
XXXXX	XXXXX	XXXXX	XXXXX	XXXXX	XXXXX	XXXXX
XXXXX	XXXXX	XXXXX	XXXXX	XXXXX	XXXXX	XXXXX
XXXXX	XXXXX	XXXXX	XXXXX	XXXXX	XXXXX	XXXXX
XXXXX	XXXXX	XXXXX	XXXXX	XXXXX	XXXXX	XXXXX
XXXXX	XXXXX	XXXXX	XXXXX	XXXXX	XXXXX	XXXXX
XXXXX	XXXXX	XXXXX	XXXXX	XXXXX	XXXXX	XXXXX
XXXXX	XXXXX	XXXXX	XXXXX	XXXXX	XXXXX	XXXXX
XXXXX	XXXXX	XXXXX	XXXXX	XXXXX	XXXXX	XXXXX
XXXXX	XXXXX	XXXXX	XXXXX	XXXXX	XXXXX	XXXXX
XXXXX	XXXXX	XXXXX	XXXXX	XXXXX	XXXXX	XXXXX
XXXXX	XXXXX	XXXXX	XXXXX	XXXXX	XXXXX	XXXXX
XXXXX	XXXXX	XXXXX	XXXXX	XXXXX	XXXXX	XXXXX
XXXXX	XXXXX	XXXXX	XXXXX	XXXXX	XXXXX	XXXXX
XXXXX	XXXXX	XXXXX	XXXXX	XXXXX	XXXXX	XXXXX
XXXXX	XXXXX	XXXXX	XXXXX	XXXXX	XXXXX	XXXXX
XXXXX	XXXXX	XXXXX	XXXXX	XXXXX	XXXXX	XXXXX
XXXXX	XXXXX	XXXXX	XXXXX	XXXXX	XXXXX	XXXXX
XXXXX	XXXXX	XXXXX	XXXXX	XXXXX	XXXXX	XXXXX
XXXXX	XXXXX	XXXXX	XXXXX	XXXXX	XXXXX	XXXXX
XXXXX	XXXXX	XXXXX	XXXXX	XXXXX	XXXXX	XXXXX
XXXXX	XXXXX	XXXXX	XXXXX	XXXXX	XXXXX	XXXXX
XXXXX	XXXXX	XXXXX	XXXXX	XXXXX	XXXXX	XXXXX
XXXXX	XXXXX	XXXXX	XXXXX	XXXXX	XXXXX	XXXXX
XXXXX	XXXXX	XXXXX	XXXXX	XXXXX	XXXXX	XXXXX
XXXXX	XXXXX	XXXXX	XXXXX	XXXXX	XXXXX	XXXXX
XXXXX	XXXXX	XXXXX	XXXXX	XXXXX	XXXXX	XXXXX
XXXXX	XXXXX	XXXXX	XXXXX	XXXXX	XXXXX	XXXXX
XXXXX	XXXXX	XXXXX	XXXXX	XXXXX	XXXXX	XXXXX
XXXXX	XXXXX	XXXXX	XXXXX	XXXXX	XXXXX	XXXXX
XXXXX	XXXXX	XXXXX	XXXXX	XXXXX	XXXXX	XXXXX

XXXXX	XXXXX	XXXXX	XXXXX	XXXXX	XXXXX	XXXXX
XXXXX	XXXXX	XXXXX	XXXXX	XXXXX	XXXXX	XXXXX
XXXXX	XXXXX	XXXXX	XXXXX	XXXXX	XXXXX	XXXXX
XXXXX	XXXXX	XXXXX	XXXXX	XXXXX	XXXXX	XXXXX
XXXXX	XXXXX	XXXXX	XXXXX	XXXXX	XXXXX	XXXXX
XXXXX	XXXXX	XXXXX	XXXXX	XXXXX	XXXXX	XXXXX
XXXXX	XXXXX	XXXXX	XXXXX	XXXXX	XXXXX	XXXXX
XXXXX	XXXXX	XXXXX	XXXXX	XXXXX	XXXXX	XXXXX
XXXXX	XXXXX	XXXXX	XXXXX	XXXXX	XXXXX	XXXXX
XXXXX	XXXXX	XXXXX	XXXXX	XXXXX	XXXXX	XXXXX
XXXXX	XXXXX	XXXXX	XXXXX	XXXXX	XXXXX	XXXXX
XXXXX	XXXXX	XXXXX	XXXXX	XXXXX	XXXXX	XXXXX
XXXXX	XXXXX	XXXXX	XXXXX	XXXXX	XXXXX	XXXXX
XXXXX	XXXXX	XXXXX	XXXXX	XXXXX	XXXXX	XXXXX
XXXXX	XXXXX	XXXXX	XXXXX	XXXXX	XXXXX	XXXXX
XXXXX	XXXXX	XXXXX	XXXXX	XXXXX	XXXXX	XXXXX
XXXXX	XXXXX	XXXXX	XXXXX	XXXXX	XXXXX	XXXXX
XXXXX	XXXXX	XXXXX	XXXXX	XXXXX	XXXXX	XXXXX
XXXXX	XXXXX	XXXXX	XXXXX	XXXXX	XXXXX	XXXXX
XXXXX	XXXXX	XXXXX	XXXXX	XXXXX	XXXXX	XXXXX
XXXXX	XXXXX	XXXXX	XXXXX	XXXXX	XXXXX	XXXXX
XXXXX	XXXXX	XXXXX	XXXXX	XXXXX	XXXXX	XXXXX
XXXXX	XXXXX	XXXXX	XXXXX	XXXXX	XXXXX	XXXXX
XXXXX	XXXXX	XXXXX	XXXXX	XXXXX	XXXXX	XXXXX
XXXXX	XXXXX	XXXXX	XXXXX	XXXXX	XXXXX	XXXXX
XXXXX	XXXXX	XXXXX	XXXXX	XXXXX	XXXXX	XXXXX
XXXXX	XXXXX	XXXXX	XXXXX	XXXXX	XXXXX	XXXXX
XXXXX	XXXXX	XXXXX	XXXXX	XXXXX	XXXXX	XXXXX
XXXXX	XXXXX	XXXXX	XXXXX	XXXXX	XXXXX	XXXXX
XXXXX	XXXXX	XXXXX	XXXXX	XXXXX	XXXXX	XXXXX
XXXXX	XXXXX	XXXXX	XXXXX	XXXXX	XXXXX	XXXXX
XXXXX	XXXXX	XXXXX	XXXXX	XXXXX	XXXXX	XXXXX
XXXXX	XXXXX	XXXXX	XXXXX	XXXXX	XXXXX	XXXXX
XXXXX	XXXXX	XXXXX	XXXXX	XXXXX	XXXXX	XXXXX
XXXXX	XXXXX	XXXXX	XXXXX	XXXXX	XXXXX	XXXXX
XXXXX	XXXXX	XXXXX	XXXXX	XXXXX	XXXXX	XXXXX
XXXXX	XXXXX	XXXXX	XXXXX	XXXXX	XXXXX	XXXXX
XXXXX	XXXXX	XXXXX	XXXXX	XXXXX	XXXXX	XXXXX
XXXXX	XXXXX	XXXXX	XXXXX	XXXXX	XXXXX	XXXXX
XXXXX	XXXXX	XXXXX	XXXXX	XXXXX	XXXXX	XXXXX
XXXXX	XXXXX	XXXXX	XXXXX	XXXXX	XXXXX	XXXXX
XXXXX	XXXXX	XXXXX	XXXXX	XXXXX	XXXXX	XXXXX
XXXXX	XXXXX	XXXXX	XXXXX	XXXXX	XXXXX	XXXXX
XXXXX	XXXXX	XXXXX	XXXXX	XXXXX	XXXXX	XXXXX
XXXXX	XXXXX	XXXXX	XXXXX	XXXXX	XXXXX	XXXXX
XXXXX	XXXXX	XXXXX	XXXXX	XXXXX	XXXXX	XXXXX
XXXXX	XXXXX	XXXXX	XXXXX	XXXXX	XXXXX	XXXXX
XXXXX	XXXXX	XXXXX	XXXXX	XXXXX	XXXXX	XXXXX
XXXXX	XXXXX	XXXXX	XXXXX	XXXXX	XXXXX	XXXXX
XXXXX	XXXXX	XXXXX	XXXXX	XXXXX	XXXXX	XXXXX

XXXXX	XXXXX	XXXXX	XXXXX	XXXXX	XXXXX	XXXXX
XXXXX	XXXXX	XXXXX	XXXXX	XXXXX	XXXXX	XXXXX
XXXXX	XXXXX	XXXXX	XXXXX	XXXXX	XXXXX	XXXXX
XXXXX	XXXXX	XXXXX	XXXXX	XXXXX	XXXXX	XXXXX
XXXXX	XXXXX	XXXXX	XXXXX	XXXXX	XXXXX	XXXXX
XXXXX	XXXXX	XXXXX	XXXXX	XXXXX	XXXXX	XXXXX
XXXXX	XXXXX	XXXXX	XXXXX	XXXXX	XXXXX	XXXXX
XXXXX	XXXXX	XXXXX	XXXXX	XXXXX	XXXXX	XXXXX
XXXXX	XXXXX	XXXXX	XXXXX	XXXXX	XXXXX	XXXXX
XXXXX	XXXXX	XXXXX	XXXXX	XXXXX	XXXXX	XXXXX
XXXXX	XXXXX	XXXXX	XXXXX	XXXXX	XXXXX	XXXXX
XXXXX	XXXXX	XXXXX	XXXXX	XXXXX	XXXXX	XXXXX
XXXXX	XXXXX	XXXXX	XXXXX	XXXXX	XXXXX	XXXXX
XXXXX	XXXXX	XXXXX	XXXXX	XXXXX	XXXXX	XXXXX
XXXXX	XXXXX	XXXXX	XXXXX	XXXXX	XXXXX	XXXXX
XXXXX	XXXXX	XXXXX	XXXXX	XXXXX	XXXXX	XXXXX
XXXXX	XXXXX	XXXXX	XXXXX	XXXXX	XXXXX	XXXXX
XXXXX	XXXXX	XXXXX	XXXXX	XXXXX	XXXXX	XXXXX
XXXXX	XXXXX	XXXXX	XXXXX	XXXXX	XXXXX	XXXXX
XXXXX	XXXXX	XXXXX	XXXXX	XXXXX	XXXXX	XXXXX
XXXXX	XXXXX	XXXXX	XXXXX	XXXXX	XXXXX	XXXXX
XXXXX	XXXXX	XXXXX	XXXXX	XXXXX	XXXXX	XXXXX
XXXXX	XXXXX	XXXXX	XXXXX	XXXXX	XXXXX	XXXXX
XXXXX	XXXXX	XXXXX	XXXXX	XXXXX	XXXXX	XXXXX
XXXXX	XXXXX	XXXXX	XXXXX	XXXXX	XXXXX	XXXXX
XXXXX	XXXXX	XXXXX	XXXXX	XXXXX	XXXXX	XXXXX
XXXXX	XXXXX	XXXXX	XXXXX	XXXXX	XXXXX	XXXXX
XXXXX	XXXXX	XXXXX	XXXXX	XXXXX	XXXXX	XXXXX
XXXXX	XXXXX	XXXXX	XXXXX	XXXXX	XXXXX	XXXXX
XXXXX	XXXXX	XXXXX	XXXXX	XXXXX	XXXXX	XXXXX
XXXXX	XXXXX	XXXXX	XXXXX	XXXXX	XXXXX	XXXXX
XXXXX	XXXXX	XXXXX	XXXXX	XXXXX	XXXXX	XXXXX
XXXXX	XXXXX	XXXXX	XXXXX	XXXXX	XXXXX	XXXXX
XXXXX	XXXXX	XXXXX	XXXXX	XXXXX	XXXXX	XXXXX
XXXXX	XXXXX	XXXXX	XXXXX	XXXXX	XXXXX	XXXXX
XXXXX	XXXXX	XXXXX	XXXXX	XXXXX	XXXXX	XXXXX
XXXXX	XXXXX	XXXXX	XXXXX	XXXXX	XXXXX	XXXXX
XXXXX	XXXXX	XXXXX	XXXXX	XXXXX	XXXXX	XXXXX
XXXXX	XXXXX	XXXXX	XXXXX	XXXXX	XXXXX	XXXXX
XXXXX	XXXXX	XXXXX	XXXXX	XXXXX	XXXXX	XXXXX
XXXXX	XXXXX	XXXXX	XXXXX	XXXXX	XXXXX	XXXXX
XXXXX	XXXXX	XXXXX	XXXXX	XXXXX	XXXXX	XXXXX
XXXXX	XXXXX	XXXXX	XXXXX	XXXXX	XXXXX	XXXXX
XXXXX	XXXXX	XXXXX	XXXXX	XXXXX	XXXXX	XXXXX
XXXXX	XXXXX	XXXXX	XXXXX	XXXXX	XXXXX	XXXXX
XXXXX	XXXXX	XXXXX	XXXXX	XXXXX	XXXXX	XXXXX
XXXXX	XXXXX	XXXXX	XXXXX	XXXXX	XXXXX	XXXXX
XXXXX	XXXXX	XXXXX	XXXXX	XXXXX	XXXXX	XXXXX
XXXXX	XXXXX	XXXXX	XXXXX	XXXXX	XXXXX	XXXXX
XXXXX	XXXXX	XXXXX	XXXXX	XXXXX	XXXXX	XXXXX

XXXXX	XXXXX	XXXXX	XXXXX	XXXXX	XXXXX	XXXXX
XXXXX	XXXXX	XXXXX	XXXXX	XXXXX	XXXXX	XXXXX
XXXXX	XXXXX	XXXXX	XXXXX	XXXXX	XXXXX	XXXXX
XXXXX	XXXXX	XXXXX	XXXXX	XXXXX	XXXXX	XXXXX
XXXXX	XXXXX	XXXXX	XXXXX	XXXXX	XXXXX	XXXXX
XXXXX	XXXXX	XXXXX	XXXXX	XXXXX	XXXXX	XXXXX
XXXXX	XXXXX	XXXXX	XXXXX	XXXXX	XXXXX	XXXXX
XXXXX	XXXXX	XXXXX	XXXXX	XXXXX	XXXXX	XXXXX
XXXXX	XXXXX	XXXXX	XXXXX	XXXXX	XXXXX	XXXXX
XXXXX	XXXXX	XXXXX	XXXXX	XXXXX	XXXXX	XXXXX
XXXXX	XXXXX	XXXXX	XXXXX	XXXXX	XXXXX	XXXXX
XXXXX	XXXXX	XXXXX	XXXXX	XXXXX	XXXXX	XXXXX
XXXXX	XXXXX	XXXXX	XXXXX	XXXXX	XXXXX	XXXXX
XXXXX	XXXXX	XXXXX	XXXXX	XXXXX	XXXXX	XXXXX
XXXXX	XXXXX	XXXXX	XXXXX	XXXXX	XXXXX	XXXXX
XXXXX	XXXXX	XXXXX	XXXXX	XXXXX	XXXXX	XXXXX
XXXXX	XXXXX	XXXXX	XXXXX	XXXXX	XXXXX	XXXXX
XXXXX	XXXXX	XXXXX	XXXXX	XXXXX	XXXXX	XXXXX
XXXXX	XXXXX	XXXXX	XXXXX	XXXXX	XXXXX	XXXXX
XXXXX	XXXXX	XXXXX	XXXXX	XXXXX	XXXXX	XXXXX
XXXXX	XXXXX	XXXXX	XXXXX	XXXXX	XXXXX	XXXXX
XXXXX	XXXXX	XXXXX	XXXXX	XXXXX	XXXXX	XXXXX
XXXXX	XXXXX	XXXXX	XXXXX	XXXXX	XXXXX	XXXXX
XXXXX	XXXXX	XXXXX	XXXXX	XXXXX	XXXXX	XXXXX
XXXXX	XXXXX	XXXXX	XXXXX	XXXXX	XXXXX	XXXXX
XXXXX	XXXXX	XXXXX	XXXXX	XXXXX	XXXXX	XXXXX
XXXXX	XXXXX	XXXXX	XXXXX	XXXXX	XXXXX	XXXXX
XXXXX	XXXXX	XXXXX	XXXXX	XXXXX	XXXXX	XXXXX
XXXXX	XXXXX	XXXXX	XXXXX	XXXXX	XXXXX	XXXXX
XXXXX	XXXXX	XXXXX	XXXXX	XXXXX	XXXXX	XXXXX
XXXXX	XXXXX	XXXXX	XXXXX	XXXXX	XXXXX	XXXXX
XXXXX	XXXXX	XXXXX	XXXXX	XXXXX	XXXXX	XXXXX
XXXXX	XXXXX	XXXXX	XXXXX	XXXXX	XXXXX	XXXXX
XXXXX	XXXXX	XXXXX	XXXXX	XXXXX	XXXXX	XXXXX
XXXXX	XXXXX	XXXXX	XXXXX	XXXXX	XXXXX	XXXXX
XXXXX	XXXXX	XXXXX	XXXXX	XXXXX	XXXXX	XXXXX
XXXXX	XXXXX	XXXXX	XXXXX	XXXXX	XXXXX	XXXXX
XXXXX	XXXXX	XXXXX	XXXXX	XXXXX	XXXXX	XXXXX
XXXXX	XXXXX	XXXXX	XXXXX	XXXXX	XXXXX	XXXXX
XXXXX	XXXXX	XXXXX	XXXXX	XXXXX	XXXXX	XXXXX
XXXXX	XXXXX	XXXXX	XXXXX	XXXXX	XXXXX	XXXXX
XXXXX	XXXXX	XXXXX	XXXXX	XXXXX	XXXXX	XXXXX
XXXXX	XXXXX	XXXXX	XXXXX	XXXXX	XXXXX	XXXXX
XXXXX	XXXXX	XXXXX	XXXXX	XXXXX	XXXXX	XXXXX
XXXXX	XXXXX	XXXXX	XXXXX	XXXXX	XXXXX	XXXXX
XXXXX	XXXXX	XXXXX	XXXXX	XXXXX	XXXXX	XXXXX
XXXXX	XXXXX	XXXXX	XXXXX	XXXXX	XXXXX	XXXXX
XXXXX	XXXXX	XXXXX	XXXXX	XXXXX	XXXXX	XXXXX
XXXXX	XXXXX	XXXXX	XXXXX	XXXXX	XXXXX	XXXXX
XXXXX	XXXXX	XXXXX	XXXXX	XXXXX	XXXXX	XXXXX

XXXXX	XXXXX	XXXXX	XXXXX	XXXXX	XXXXX	XXXXX
XXXXX	XXXXX	XXXXX	XXXXX	XXXXX	XXXXX	XXXXX
XXXXX	XXXXX	XXXXX	XXXXX	XXXXX	XXXXX	XXXXX
XXXXX	XXXXX	XXXXX	XXXXX	XXXXX	XXXXX	XXXXX
XXXXX	XXXXX	XXXXX	XXXXX	XXXXX	XXXXX	XXXXX
XXXXX	XXXXX	XXXXX	XXXXX	XXXXX	XXXXX	XXXXX
XXXXX	XXXXX	XXXXX	XXXXX	XXXXX	XXXXX	XXXXX
XXXXX	XXXXX	XXXXX	XXXXX	XXXXX	XXXXX	XXXXX
XXXXX	XXXXX	XXXXX	XXXXX	XXXXX	XXXXX	XXXXX
XXXXX	XXXXX	XXXXX	XXXXX	XXXXX	XXXXX	XXXXX
XXXXX	XXXXX	XXXXX	XXXXX	XXXXX	XXXXX	XXXXX
XXXXX	XXXXX	XXXXX	XXXXX	XXXXX	XXXXX	XXXXX
XXXXX	XXXXX	XXXXX	XXXXX	XXXXX	XXXXX	XXXXX
XXXXX	XXXXX	XXXXX	XXXXX	XXXXX	XXXXX	XXXXX
XXXXX	XXXXX	XXXXX	XXXXX	XXXXX	XXXXX	XXXXX
XXXXX	XXXXX	XXXXX	XXXXX	XXXXX	XXXXX	XXXXX
XXXXX	XXXXX	XXXXX	XXXXX	XXXXX	XXXXX	XXXXX
XXXXX	XXXXX	XXXXX	XXXXX	XXXXX	XXXXX	XXXXX
XXXXX	XXXXX	XXXXX	XXXXX	XXXXX	XXXXX	XXXXX
XXXXX	XXXXX	XXXXX	XXXXX	XXXXX	XXXXX	XXXXX
XXXXX	XXXXX	XXXXX	XXXXX	XXXXX	XXXXX	XXXXX
XXXXX	XXXXX	XXXXX	XXXXX	XXXXX	XXXXX	XXXXX
XXXXX	XXXXX	XXXXX	XXXXX	XXXXX	XXXXX	XXXXX
XXXXX	XXXXX	XXXXX	XXXXX	XXXXX	XXXXX	XXXXX
XXXXX	XXXXX	XXXXX	XXXXX	XXXXX	XXXXX	XXXXX
XXXXX	XXXXX	XXXXX	XXXXX	XXXXX	XXXXX	XXXXX
XXXXX	XXXXX	XXXXX	XXXXX	XXXXX	XXXXX	XXXXX
XXXXX	XXXXX	XXXXX	XXXXX	XXXXX	XXXXX	XXXXX
XXXXX	XXXXX	XXXXX	XXXXX	XXXXX	XXXXX	XXXXX
XXXXX	XXXXX	XXXXX	XXXXX	XXXXX	XXXXX	XXXXX
XXXXX	XXXXX	XXXXX	XXXXX	XXXXX	XXXXX	XXXXX
XXXXX	XXXXX	XXXXX	XXXXX	XXXXX	XXXXX	XXXXX
XXXXX	XXXXX	XXXXX	XXXXX	XXXXX	XXXXX	XXXXX
XXXXX	XXXXX	XXXXX	XXXXX	XXXXX	XXXXX	XXXXX
XXXXX	XXXXX	XXXXX	XXXXX	XXXXX	XXXXX	XXXXX
XXXXX	XXXXX	XXXXX	XXXXX	XXXXX	XXXXX	XXXXX
XXXXX	XXXXX	XXXXX	XXXXX	XXXXX	XXXXX	XXXXX
XXXXX	XXXXX	XXXXX	XXXXX	XXXXX	XXXXX	XXXXX
XXXXX	XXXXX	XXXXX	XXXXX	XXXXX	XXXXX	XXXXX
XXXXX	XXXXX	XXXXX	XXXXX	XXXXX	XXXXX	XXXXX
XXXXX	XXXXX	XXXXX	XXXXX	XXXXX	XXXXX	XXXXX
XXXXX	XXXXX	XXXXX	XXXXX	XXXXX	XXXXX	XXXXX
XXXXX	XXXXX	XXXXX	XXXXX	XXXXX	XXXXX	XXXXX
XXXXX	XXXXX	XXXXX	XXXXX	XXXXX	XXXXX	XXXXX
XXXXX	XXXXX	XXXXX	XXXXX	XXXXX	XXXXX	XXXXX
XXXXX	XXXXX	XXXXX	XXXXX	XXXXX	XXXXX	XXXXX
XXXXX	XXXXX	XXXXX	XXXXX	XXXXX	XXXXX	XXXXX
XXXXX	XXXXX	XXXXX	XXXXX	XXXXX	XXXXX	XXXXX
XXXXX	XXXXX	XXXXX	XXXXX	XXXXX	XXXXX	XXXXX
XXXXX	XXXXX	XXXXX	XXXXX	XXXXX	XXXXX	XXXXX

XXXXX	XXXXX	XXXXX	XXXXX	XXXXX	XXXXX	XXXXX
XXXXX	XXXXX	XXXXX	XXXXX	XXXXX	XXXXX	XXXXX
XXXXX	XXXXX	XXXXX	XXXXX	XXXXX	XXXXX	XXXXX
XXXXX	XXXXX	XXXXX	XXXXX	XXXXX	XXXXX	XXXXX
XXXXX	XXXXX	XXXXX	XXXXX	XXXXX	XXXXX	XXXXX
XXXXX	XXXXX	XXXXX	XXXXX	XXXXX	XXXXX	XXXXX
XXXXX	XXXXX	XXXXX	XXXXX	XXXXX	XXXXX	XXXXX
XXXXX	XXXXX	XXXXX	XXXXX	XXXXX	XXXXX	XXXXX
XXXXX	XXXXX	XXXXX	XXXXX	XXXXX	XXXXX	XXXXX
XXXXX	XXXXX	XXXXX	XXXXX	XXXXX	XXXXX	XXXXX
XXXXX	XXXXX	XXXXX	XXXXX	XXXXX	XXXXX	XXXXX
XXXXX	XXXXX	XXXXX	XXXXX	XXXXX	XXXXX	XXXXX
XXXXX	XXXXX	XXXXX	XXXXX	XXXXX	XXXXX	XXXXX
XXXXX	XXXXX	XXXXX	XXXXX	XXXXX	XXXXX	XXXXX
XXXXX	XXXXX	XXXXX	XXXXX	XXXXX	XXXXX	XXXXX
XXXXX	XXXXX	XXXXX	XXXXX	XXXXX	XXXXX	XXXXX
XXXXX	XXXXX	XXXXX	XXXXX	XXXXX	XXXXX	XXXXX
XXXXX	XXXXX	XXXXX	XXXXX	XXXXX	XXXXX	XXXXX
XXXXX	XXXXX	XXXXX	XXXXX	XXXXX	XXXXX	XXXXX
XXXXX	XXXXX	XXXXX	XXXXX	XXXXX	XXXXX	XXXXX
XXXXX	XXXXX	XXXXX	XXXXX	XXXXX	XXXXX	XXXXX
XXXXX	XXXXX	XXXXX	XXXXX	XXXXX	XXXXX	XXXXX
XXXXX	XXXXX	XXXXX	XXXXX	XXXXX	XXXXX	XXXXX
XXXXX	XXXXX	XXXXX	XXXXX	XXXXX	XXXXX	XXXXX
XXXXX	XXXXX	XXXXX	XXXXX	XXXXX	XXXXX	XXXXX
XXXXX	XXXXX	XXXXX	XXXXX	XXXXX	XXXXX	XXXXX
XXXXX	XXXXX	XXXXX	XXXXX	XXXXX	XXXXX	XXXXX
XXXXX	XXXXX	XXXXX	XXXXX	XXXXX	XXXXX	XXXXX
XXXXX	XXXXX	XXXXX	XXXXX	XXXXX	XXXXX	XXXXX
XXXXX	XXXXX	XXXXX	XXXXX	XXXXX	XXXXX	XXXXX
XXXXX	XXXXX	XXXXX	XXXXX	XXXXX	XXXXX	XXXXX
XXXXX	XXXXX	XXXXX	XXXXX	XXXXX	XXXXX	XXXXX
XXXXX	XXXXX	XXXXX	XXXXX	XXXXX	XXXXX	XXXXX
XXXXX	XXXXX	XXXXX	XXXXX	XXXXX	XXXXX	XXXXX
XXXXX	XXXXX	XXXXX	XXXXX	XXXXX	XXXXX	XXXXX
XXXXX	XXXXX	XXXXX	XXXXX	XXXXX	XXXXX	XXXXX
XXXXX	XXXXX	XXXXX	XXXXX	XXXXX	XXXXX	XXXXX
XXXXX	XXXXX	XXXXX	XXXXX	XXXXX	XXXXX	XXXXX
XXXXX	XXXXX	XXXXX	XXXXX	XXXXX	XXXXX	XXXXX
XXXXX	XXXXX	XXXXX	XXXXX	XXXXX	XXXXX	XXXXX
XXXXX	XXXXX	XXXXX	XXXXX	XXXXX	XXXXX	XXXXX
XXXXX	XXXXX	XXXXX	XXXXX	XXXXX	XXXXX	XXXXX
XXXXX	XXXXX	XXXXX	XXXXX	XXXXX	XXXXX	XXXXX
XXXXX	XXXXX	XXXXX	XXXXX	XXXXX	XXXXX	XXXXX
XXXXX	XXXXX	XXXXX	XXXXX	XXXXX	XXXXX	XXXXX
XXXXX	XXXXX	XXXXX	XXXXX	XXXXX	XXXXX	XXXXX
XXXXX	XXXXX	XXXXX	XXXXX	XXXXX	XXXXX	XXXXX
XXXXX	XXXXX	XXXXX	XXXXX	XXXXX	XXXXX	XXXXX
XXXXX	XXXXX	XXXXX	XXXXX	XXXXX	XXXXX	XXXXX
XXXXX	XXXXX	XXXXX	XXXXX	XXXXX	XXXXX	XXXXX

XXXXX	XXXXX	XXXXX	XXXXX	XXXXX	XXXXX	XXXXX
XXXXX	XXXXX	XXXXX	XXXXX	XXXXX	XXXXX	XXXXX
XXXXX	XXXXX	XXXXX	XXXXX	XXXXX	XXXXX	XXXXX
XXXXX	XXXXX	XXXXX	XXXXX	XXXXX	XXXXX	XXXXX
XXXXX	XXXXX	XXXXX	XXXXX	XXXXX	XXXXX	XXXXX
XXXXX	XXXXX	XXXXX	XXXXX	XXXXX	XXXXX	XXXXX
XXXXX	XXXXX	XXXXX	XXXXX	XXXXX	XXXXX	XXXXX
XXXXX	XXXXX	XXXXX	XXXXX	XXXXX	XXXXX	XXXXX
XXXXX	XXXXX	XXXXX	XXXXX	XXXXX	XXXXX	XXXXX
XXXXX	XXXXX	XXXXX	XXXXX	XXXXX	XXXXX	XXXXX
XXXXX	XXXXX	XXXXX	XXXXX	XXXXX	XXXXX	XXXXX
XXXXX	XXXXX	XXXXX	XXXXX	XXXXX	XXXXX	XXXXX
XXXXX	XXXXX	XXXXX	XXXXX	XXXXX	XXXXX	XXXXX
XXXXX	XXXXX	XXXXX	XXXXX	XXXXX	XXXXX	XXXXX
XXXXX	XXXXX	XXXXX	XXXXX	XXXXX	XXXXX	XXXXX
XXXXX	XXXXX	XXXXX	XXXXX	XXXXX	XXXXX	XXXXX
XXXXX	XXXXX	XXXXX	XXXXX	XXXXX	XXXXX	XXXXX
XXXXX	XXXXX	XXXXX	XXXXX	XXXXX	XXXXX	XXXXX
XXXXX	XXXXX	XXXXX	XXXXX	XXXXX	XXXXX	XXXXX
XXXXX	XXXXX	XXXXX	XXXXX	XXXXX	XXXXX	XXXXX
XXXXX	XXXXX	XXXXX	XXXXX	XXXXX	XXXXX	XXXXX
XXXXX	XXXXX	XXXXX	XXXXX	XXXXX	XXXXX	XXXXX
XXXXX	XXXXX	XXXXX	XXXXX	XXXXX	XXXXX	XXXXX
XXXXX	XXXXX	XXXXX	XXXXX	XXXXX	XXXXX	XXXXX
XXXXX	XXXXX	XXXXX	XXXXX	XXXXX	XXXXX	XXXXX
XXXXX	XXXXX	XXXXX	XXXXX	XXXXX	XXXXX	XXXXX
XXXXX	XXXXX	XXXXX	XXXXX	XXXXX	XXXXX	XXXXX
XXXXX	XXXXX	XXXXX	XXXXX	XXXXX	XXXXX	XXXXX
XXXXX	XXXXX	XXXXX	XXXXX	XXXXX	XXXXX	XXXXX
XXXXX	XXXXX	XXXXX	XXXXX	XXXXX	XXXXX	XXXXX
XXXXX	XXXXX	XXXXX	XXXXX	XXXXX	XXXXX	XXXXX
XXXXX	XXXXX	XXXXX	XXXXX	XXXXX	XXXXX	XXXXX
XXXXX	XXXXX	XXXXX	XXXXX	XXXXX	XXXXX	XXXXX
XXXXX	XXXXX	XXXXX	XXXXX	XXXXX	XXXXX	XXXXX
XXXXX	XXXXX	XXXXX	XXXXX	XXXXX	XXXXX	XXXXX
XXXXX	XXXXX	XXXXX	XXXXX	XXXXX	XXXXX	XXXXX
XXXXX	XXXXX	XXXXX	XXXXX	XXXXX	XXXXX	XXXXX
XXXXX	XXXXX	XXXXX	XXXXX	XXXXX	XXXXX	XXXXX
XXXXX	XXXXX	XXXXX	XXXXX	XXXXX	XXXXX	XXXXX
XXXXX	XXXXX	XXXXX	XXXXX	XXXXX	XXXXX	XXXXX
XXXXX	XXXXX	XXXXX	XXXXX	XXXXX	XXXXX	XXXXX
XXXXX	XXXXX	XXXXX	XXXXX	XXXXX	XXXXX	XXXXX
XXXXX	XXXXX	XXXXX	XXXXX	XXXXX	XXXXX	XXXXX
XXXXX	XXXXX	XXXXX	XXXXX	XXXXX	XXXXX	XXXXX
XXXXX	XXXXX	XXXXX	XXXXX	XXXXX	XXXXX	XXXXX
XXXXX	XXXXX	XXXXX	XXXXX	XXXXX	XXXXX	XXXXX
XXXXX	XXXXX	XXXXX	XXXXX	XXXXX	XXXXX	XXXXX
XXXXX	XXXXX	XXXXX	XXXXX	XXXXX	XXXXX	XXXXX
XXXXX	XXXXX	XXXXX	XXXXX	XXXXX	XXXXX	XXXXX
XXXXX	XXXXX	XXXXX	XXXXX	XXXXX	XXXXX	XXXXX

XXXXX	XXXXX	XXXXX	XXXXX	XXXXX	XXXXX	XXXXX
XXXXX	XXXXX	XXXXX	XXXXX	XXXXX	XXXXX	XXXXX
XXXXX	XXXXX	XXXXX	XXXXX	XXXXX	XXXXX	XXXXX
XXXXX	XXXXX	XXXXX	XXXXX	XXXXX	XXXXX	XXXXX
XXXXX	XXXXX	XXXXX	XXXXX	XXXXX	XXXXX	XXXXX
XXXXX	XXXXX	XXXXX	XXXXX	XXXXX	XXXXX	XXXXX
XXXXX	XXXXX	XXXXX	XXXXX	XXXXX	XXXXX	XXXXX
XXXXX	XXXXX	XXXXX	XXXXX	XXXXX	XXXXX	XXXXX
XXXXX	XXXXX	XXXXX	XXXXX	XXXXX	XXXXX	XXXXX
XXXXX	XXXXX	XXXXX	XXXXX	XXXXX	XXXXX	XXXXX
XXXXX	XXXXX	XXXXX	XXXXX	XXXXX	XXXXX	XXXXX
XXXXX	XXXXX	XXXXX	XXXXX	XXXXX	XXXXX	XXXXX
XXXXX	XXXXX	XXXXX	XXXXX	XXXXX	XXXXX	XXXXX
XXXXX	XXXXX	XXXXX	XXXXX	XXXXX	XXXXX	XXXXX
XXXXX	XXXXX	XXXXX	XXXXX	XXXXX	XXXXX	XXXXX
XXXXX	XXXXX	XXXXX	XXXXX	XXXXX	XXXXX	XXXXX
XXXXX	XXXXX	XXXXX	XXXXX	XXXXX	XXXXX	XXXXX
XXXXX	XXXXX	XXXXX	XXXXX	XXXXX	XXXXX	XXXXX
XXXXX	XXXXX	XXXXX	XXXXX	XXXXX	XXXXX	XXXXX
XXXXX	XXXXX	XXXXX	XXXXX	XXXXX	XXXXX	XXXXX
XXXXX	XXXXX	XXXXX	XXXXX	XXXXX	XXXXX	XXXXX
XXXXX	XXXXX	XXXXX	XXXXX	XXXXX	XXXXX	XXXXX
XXXXX	XXXXX	XXXXX	XXXXX	XXXXX	XXXXX	XXXXX
XXXXX	XXXXX	XXXXX	XXXXX	XXXXX	XXXXX	XXXXX
XXXXX	XXXXX	XXXXX	XXXXX	XXXXX	XXXXX	XXXXX
XXXXX	XXXXX	XXXXX	XXXXX	XXXXX	XXXXX	XXXXX
XXXXX	XXXXX	XXXXX	XXXXX	XXXXX	XXXXX	XXXXX
XXXXX	XXXXX	XXXXX	XXXXX	XXXXX	XXXXX	XXXXX
XXXXX	XXXXX	XXXXX	XXXXX	XXXXX	XXXXX	XXXXX
XXXXX	XXXXX	XXXXX	XXXXX	XXXXX	XXXXX	XXXXX
XXXXX	XXXXX	XXXXX	XXXXX	XXXXX	XXXXX	XXXXX
XXXXX	XXXXX	XXXXX	XXXXX	XXXXX	XXXXX	XXXXX
XXXXX	XXXXX	XXXXX	XXXXX	XXXXX	XXXXX	XXXXX
XXXXX	XXXXX	XXXXX	XXXXX	XXXXX	XXXXX	XXXXX
XXXXX	XXXXX	XXXXX	XXXXX	XXXXX	XXXXX	XXXXX
XXXXX	XXXXX	XXXXX	XXXXX	XXXXX	XXXXX	XXXXX
XXXXX	XXXXX	XXXXX	XXXXX	XXXXX	XXXXX	XXXXX
XXXXX	XXXXX	XXXXX	XXXXX	XXXXX	XXXXX	XXXXX
XXXXX	XXXXX	XXXXX	XXXXX	XXXXX	XXXXX	XXXXX
XXXXX	XXXXX	XXXXX	XXXXX	XXXXX	XXXXX	XXXXX
XXXXX	XXXXX	XXXXX	XXXXX	XXXXX	XXXXX	XXXXX
XXXXX	XXXXX	XXXXX	XXXXX	XXXXX	XXXXX	XXXXX
XXXXX	XXXXX	XXXXX	XXXXX	XXXXX	XXXXX	XXXXX
XXXXX	XXXXX	XXXXX	XXXXX	XXXXX	XXXXX	XXXXX
XXXXX	XXXXX	XXXXX	XXXXX	XXXXX	XXXXX	XXXXX
XXXXX	XXXXX	XXXXX	XXXXX	XXXXX	XXXXX	XXXXX
XXXXX	XXXXX	XXXXX	XXXXX	XXXXX	XXXXX	XXXXX
XXXXX	XXXXX	XXXXX	XXXXX	XXXXX	XXXXX	XXXXX
XXXXX	XXXXX	XXXXX	XXXXX	XXXXX	XXXXX	XXXXX
XXXXX	XXXXX	XXXXX	XXXXX	XXXXX	XXXXX	XXXXX

xxxxx	xxxxx	xxxxx	xxxxx	xxxxx	xxxxx	xxxxx
xxxxx	xxxxx	xxxxx	xxxxx	xxxxx	xxxxx	xxxxx
xxxxx	xxxxx	xxxxx	xxxxx	xxxxx	xxxxx	xxxxx
xxxxx	xxxxx	xxxxx	xxxxx	xxxxx	xxxxx	xxxxx
xxxxx	xxxxx	xxxxx	xxxxx	xxxxx	xxxxx	xxxxx
xxxxx	xxxxx	xxxxx	xxxxx	xxxxx	xxxxx	xxxxx
xxxxx	xxxxx	xxxxx	xxxxx	xxxxx	xxxxx	xxxxx
xxxxx	xxxxx	xxxxx	xxxxx	xxxxx	xxxxx	xxxxx
xxxxx	xxxxx	xxxxx	xxxxx	xxxxx	xxxxx	xxxxx
xxxxx	xxxxx	xxxxx	xxxxx	xxxxx	xxxxx	xxxxx
xxxxx	xxxxx	xxxxx	xxxxx	xxxxx	xxxxx	xxxxx
xxxxx	xxxxx	xxxxx	xxxxx	xxxxx	xxxxx	xxxxx
xxxxx	xxxxx	xxxxx	xxxxx	xxxxx	xxxxx	xxxxx
xxxxx	xxxxx	xxxxx	xxxxx	xxxxx	xxxxx	xxxxx
xxxxx	xxxxx	xxxxx	xxxxx	xxxxx	xxxxx	xxxxx
xxxxx	xxxxx	xxxxx	xxxxx	xxxxx	xxxxx	xxxxx
xxxxx	xxxxx	xxxxx	xxxxx	xxxxx	xxxxx	xxxxx
xxxxx	xxxxx	xxxxx	xxxxx	xxxxx	xxxxx	xxxxx
xxxxx	xxxxx	xxxxx	xxxxx	xxxxx	xxxxx	xxxxx
xxxxx	xxxxx	xxxxx	xxxxx	xxxxx	xxxxx	xxxxx
xxxxx	xxxxx	xxxxx	xxxxx	xxxxx	xxxxx	xxxxx
xxxxx	xxxxx	xxxxx	xxxxx	xxxxx	xxxxx	xxxxx
xxxxx	xxxxx	xxxxx	xxxxx	xxxxx	xxxxx	xxxxx
xxxxx	xxxxx	xxxxx	xxxxx	xxxxx	xxxxx	xxxxx
xxxxx	xxxxx	xxxxx	xxxxx	xxxxx	xxxxx	xxxxx
xxxxx	xxxxx	xxxxx	xxxxx	xxxxx	xxxxx	xxxxx
xxxxx	xxxxx	xxxxx	xxxxx	xxxxx	xxxxx	xxxxx
xxxxx	xxxxx	xxxxx	xxxxx	xxxxx	xxxxx	xxxxx
xxxxx	xxxxx	xxxxx	xxxxx	xxxxx	xxxxx	xxxxx
xxxxx	xxxxx	xxxxx	xxxxx	xxxxx	xxxxx	xxxxx
xxxxx	xxxxx	xxxxx	xxxxx	xxxxx	xxxxx	xxxxx
xxxxx	xxxxx	xxxxx	xxxxx	xxxxx	xxxxx	xxxxx
xxxxx	xxxxx	xxxxx	xxxxx	xxxxx	xxxxx	xxxxx
xxxxx	xxxxx	xxxxx	xxxxx	xxxxx	xxxxx	xxxxx

rank Pvalue	FDR	FDR*
3	2,56667E-06	2,56667E-06
2	0,00000385	0,00000385
1	0,0000077	0,0000077
4	0,000009625	2,56667E-06
7	0,0000253	0,0000253
6	2,56667E-05	2,56667E-05
5	0,00002618	0,000009625
10	0,00013167	0,00013167
8	0,000137638	0,0000253
9	0,000139456	0,000137638
11	0,0001897	0,00013167
12	0,000226508	0,0001897
13	0,000290823	0,000226508
17	0,000375488	0,000375488
16	0,000379225	0,000379225
18	0,000382006	0,000375488
19	0,000385	0,000382006
14	0,0003872	0,000290823
15	0,00039886	0,0003872
20	0,000415415	0,000385
21	0,0004202	0,000415415
29	0,000465186	0,000465186
28	0,000471075	0,000471075
27	0,000487667	0,000487667
26	0,000491319	0,000491319
24	0,000495688	0,000495688
25	0,0005005	0,000495688
30	0,000505377	0,000465186
22	0,00050715	0,0004202
23	0,000511883	0,00050715
31	0,000571787	0,000505377
32	0,000637656	0,000571787
34	0,000717459	0,000717459
35	0,00072842	0,000717459
33	0,000732433	0,000637656
36	0,000761231	0,00072842
37	0,000778741	0,000761231
38	0,000936563	0,000778741
39	0,001000605	0,000936563
40	0,001067413	0,001000605
42	0,001113017	0,001113017
41	0,001124012	0,001067413
43	0,001129035	0,001113017
48	0,00122029	0,00122029
47	0,001224628	0,001224628
44	0,001237775	0,001129035
46	0,001244722	0,001244722

45	0,001257667	0,001237775
49	0,001307586	0,00122029
51	0,001348104	0,001348104
50	0,001349656	0,001307586
52	0,001366306	0,001348104
53	0,001418108	0,001366306
54	0,001422789	0,001418108
55	0,00148134	0,001422789
57	0,001507039	0,001507039
58	0,001518626	0,001507039
56	0,001524463	0,00148134
59	0,001808064	0,001518626
61	0,001917174	0,001917174
60	0,001922048	0,001808064
63	0,001945289	0,001945289
62	0,001964742	0,001917174
64	0,00202498	0,001945289
65	0,002157895	0,00202498
66	0,002158917	0,002157895
67	0,00217956	0,002158917
68	0,002494347	0,00217956
69	0,0025179	0,002494347
72	0,002667622	0,002667622
71	0,002697494	0,002697494
70	0,00273152	0,0025179
73	0,002766937	0,002667622
74	0,002769295	0,002766937
77	0,0029465	0,0029465
76	0,002953355	0,002953355
75	0,002966759	0,002769295
78	0,003093031	0,0029465
79	0,003109923	0,003093031
80	0,00311311	0,003109923
81	0,003129907	0,00311311
82	0,003167611	0,003129907
84	0,003646042	0,003646042
83	0,003654995	0,003167611
85	0,003880256	0,003646042
86	0,004171609	0,003880256
87	0,004239691	0,004171609
88	0,0044982	0,004239691
89	0,004520506	0,0044982
90	0,00471009	0,004520506
91	0,004806408	0,00471009
92	0,005070283	0,004806408
93	0,005158089	0,005070283
94	0,005162932	0,005158089
95	0,00517748	0,005162932

100	0,005282277	0,005282277
96	0,005288376	0,00517748
98	0,005332014	0,005332014
99	0,005335633	0,005332014
97	0,005341974	0,005288376
102	0,005558268	0,005558268
101	0,005562983	0,005282277
103	0,00557622	0,005558268
104	0,005914044	0,00557622
105	0,0059301	0,005914044
106	0,006255451	0,0059301
107	0,00676902	0,006255451
108	0,006980763	0,00676902
109	0,006996828	0,006980763
110	0,00706293	0,006996828
111	0,007350309	0,00706293
112	0,007404444	0,007350309
114	0,007672982	0,007672982
113	0,007685281	0,007404444
116	0,0080157	0,0080157
115	0,008043822	0,007672982
117	0,008138703	0,0080157
118	0,008489707	0,008138703
119	0,008552629	0,008489707
120	0,008706583	0,008552629
121	0,009439818	0,008706583
122	0,009744729	0,009439818
123	0,009919979	0,009744729
124	0,010159777	0,009919979
125	0,010365001	0,010159777
126	0,010612617	0,010365001
127	0,011209806	0,010612617
129	0,011298228	0,011298228
131	0,01133922	0,01133922
130	0,011365496	0,011298228
128	0,01136899	0,011209806
132	0,011541367	0,01133922
133	0,011559437	0,011541367
135	0,011671546	0,011671546
134	0,011697737	0,011559437
137	0,012156277	0,012156277
136	0,012202801	0,011671546
138	0,012844437	0,012156277
142	0,013070642	0,013070642
141	0,013093713	0,013093713
140	0,013151655	0,013151655
139	0,013179242	0,012844437
143	0,013988046	0,013070642

145	0,014001627	0,014001627
144	0,014092604	0,013988046
146	0,014294786	0,014001627
147	0,014490719	0,014294786
148	0,014785249	0,014490719
149	0,014802501	0,014785249
150	0,015014795	0,014802501
151	0,015175782	0,015014795
152	0,016096242	0,015175782
153	0,016460788	0,016096242
154	0,01652525	0,016460788
155	0,016552615	0,01652525
156	0,016601644	0,016552615
157	0,01694255	0,016601644
158	0,017237473	0,01694255
159	0,017561618	0,017237473
161	0,017657726	0,017657726
160	0,017731127	0,017561618
163	0,017861496	0,017861496
162	0,017866281	0,017657726
164	0,018320976	0,017861496
165	0,018870087	0,018320976
166	0,019536199	0,018870087
167	0,019790706	0,019536199
168	0,020513992	0,019790706
169	0,021228991	0,020513992
170	0,021598908	0,021228991
171	0,022089634	0,021598908
172	0,02295088	0,022089634
173	0,023107745	0,02295088
174	0,02327002	0,023107745
175	0,026984848	0,02327002
176	0,027416156	0,026984848
177	0,028191527	0,027416156
178	0,028546928	0,028191527
180	0,029425079	0,029425079
179	0,029565161	0,028546928
181	0,029822781	0,029425079
183	0,031477895	0,031477895
182	0,031642854	0,029822781
186	0,032244081	0,032244081
184	0,032361384	0,031477895
185	0,032384618	0,032361384
187	0,033560965	0,032244081
188	0,03412939	0,033560965
189	0,034433015	0,03412939
190	0,034555979	0,034433015
191	0,035434795	0,034555979

193	0,036262532	0,036262532
192	0,036282721	0,035434795
194	0,036316494	0,036262532
195	0,037310725	0,036316494
197	0,038713489	0,038713489
196	0,038826032	0,037310725
198	0,03918355	0,038713489
199	0,040072657	0,03918355
200	0,040555862	0,040072657
201	0,041207182	0,040555862
202	0,041434081	0,041207182
203	0,043616062	0,041434081
204	0,043991232	0,043616062
205	0,044174675	0,043991232
206	0,044241696	0,044174675
207	0,044381312	0,044241696
210	0,0446204	0,0446204
208	0,044729226	0,044381312
209	0,044819895	0,044729226
211	0,045375334	0,0446204
213	0,045941164	0,045941164
212	0,04608657	0,045375334
214	0,046367349	0,045941164
215	0,047238676	0,046367349
216	0,047297963	0,047238676
217	0,048242203	0,047297963
218	0,048328626	0,048242203
219	0,048448189	0,048328626
220	0,0491638	0,048448189
221	0,051067898	0,0491638
223	0,051949897	0,051949897
222	0,051972919	0,051067898
224	0,05209105	0,051949897
229	0,052206269	0,052206269
230	0,052214939	0,052206269
228	0,052358007	0,052358007
227	0,05239599	0,05239599
225	0,052429437	0,05209105
226	0,052499179	0,052429437
231	0,052530867	0,052214939
232	0,054250748	0,052530867
233	0,054502748	0,054250748
234	0,055606899	0,054502748
235	0,05618441	0,055606899
237	0,059064556	0,059064556
236	0,059170813	0,05618441
238	0,059583991	0,059064556
239	0,060357465	0,059583991

240	0,060867666	0,060357465
241	0,061024193	0,060867666
242	0,066480209	0,061024193
243	0,06675599	0,066480209
244	0,066878823	0,06675599
246	0,066976478	0,066976478
245	0,066989591	0,066878823
247	0,067252798	0,066976478
248	0,067683404	0,067252798
250	0,069884984	0,069884984
251	0,069886458	0,069884984
252	0,070067769	0,069886458
249	0,070069165	0,067683404
253	0,070636635	0,070067769
254	0,071045263	0,070636635
255	0,072473578	0,071045263
258	0,073876934	0,073876934
257	0,074040863	0,074040863
259	0,074117257	0,073876934
256	0,07430876	0,072473578
264	0,074463521	0,074463521
263	0,074518697	0,074518697
260	0,074681619	0,074117257
262	0,074682917	0,074682917
261	0,074954573	0,074681619
265	0,0755909	0,074463521
266	0,075607111	0,0755909
267	0,076471872	0,075607111
268	0,076784946	0,076471872
269	0,078264861	0,076784946
271	0,084545574	0,084545574
270	0,084691587	0,078264861
273	0,085667549	0,085667549
272	0,085784624	0,084545574
275	0,086830548	0,086830548
274	0,086839448	0,085667549
276	0,089459074	0,086830548
277	0,089803627	0,089459074
278	0,09289593	0,089803627
281	0,093040936	0,093040936
280	0,09329375	0,09329375
279	0,093622009	0,09289593
282	0,095572127	0,093040936
283	0,096699511	0,095572127
284	0,097896932	0,096699511
285	0,098195209	0,097896932
286	0,098333604	0,098195209
287	0,099318276	0,098333604

288	0,09995993	0,099318276
289	0,100040745	0,09995993
290	0,101082467	0,100040745
293	0,101998116	0,101998116
291	0,102098243	0,101082467
292	0,102131323	0,102098243
294	0,103538888	0,101998116
295	0,104143205	0,103538888
296	0,104226628	0,104143205
297	0,104597967	0,104226628
298	0,106216177	0,104597967
299	0,108647953	0,106216177
300	0,115333885	0,108647953
301	0,116313616	0,115333885
304	0,116438463	0,116438463
302	0,116569153	0,116313616
303	0,116600465	0,116569153
305	0,116961536	0,116438463
306	0,117127267	0,116961536
307	0,118064602	0,117127267
308	0,12170495	0,118064602
309	0,123451135	0,12170495
770	XXXXX	0
769	XXXXX	0
768	XXXXX	0
767	XXXXX	0
766	XXXXX	0
765	XXXXX	0
764	XXXXX	0
763	XXXXX	0
762	XXXXX	0
761	XXXXX	0
760	XXXXX	0
759	XXXXX	0
758	XXXXX	0
757	XXXXX	0
756	XXXXX	0
755	XXXXX	0
754	XXXXX	0
753	XXXXX	0
752	XXXXX	0
751	XXXXX	0
750	XXXXX	0
749	XXXXX	0
748	XXXXX	0
747	XXXXX	0
746	XXXXX	0
745	XXXXX	0
744	XXXXX	0

743	XXXXX	0
742	XXXXX	0
741	XXXXX	0
740	XXXXX	0
739	XXXXX	0
738	XXXXX	0
737	XXXXX	0
736	XXXXX	0
735	XXXXX	0
734	XXXXX	0
733	XXXXX	0
732	XXXXX	0
731	XXXXX	0
730	XXXXX	0
729	XXXXX	0
728	XXXXX	0
727	XXXXX	0
726	XXXXX	0
725	XXXXX	0
724	XXXXX	0
723	XXXXX	0
722	XXXXX	0
721	XXXXX	0
720	XXXXX	0
719	XXXXX	0
718	XXXXX	0
717	XXXXX	0
716	XXXXX	0
715	XXXXX	0
714	XXXXX	0
713	XXXXX	0
712	XXXXX	0
711	XXXXX	0
710	XXXXX	0
709	XXXXX	0
708	XXXXX	0
707	XXXXX	0
706	XXXXX	0
705	XXXXX	0
704	XXXXX	0
703	XXXXX	0
702	XXXXX	0
701	XXXXX	0
700	XXXXX	0
699	XXXXX	0
698	XXXXX	0
697	XXXXX	0
696	XXXXX	0
695	XXXXX	0
694	XXXXX	0

693	XXXXX	0
692	XXXXX	0
691	XXXXX	0
690	XXXXX	0
689	XXXXX	0
688	XXXXX	0
687	XXXXX	0
686	XXXXX	0
685	XXXXX	0
684	XXXXX	0
683	XXXXX	0
682	XXXXX	0
681	XXXXX	0
680	XXXXX	0
679	XXXXX	0
678	XXXXX	0
677	XXXXX	0
676	XXXXX	0
675	XXXXX	0
674	XXXXX	0
673	XXXXX	0
672	XXXXX	0
671	XXXXX	0
670	XXXXX	0
669	XXXXX	0
668	XXXXX	0
667	XXXXX	0
666	XXXXX	0
665	XXXXX	0
664	XXXXX	0
663	XXXXX	0
662	XXXXX	0
661	XXXXX	0
660	XXXXX	0
659	XXXXX	0
658	XXXXX	0
657	XXXXX	0
656	XXXXX	0
655	XXXXX	0
654	XXXXX	0
653	XXXXX	0
652	XXXXX	0
651	XXXXX	0
650	XXXXX	0
649	XXXXX	0
648	XXXXX	0
647	XXXXX	0
646	XXXXX	0
645	XXXXX	0
644	XXXXX	0

643	XXXXX	0
642	XXXXX	0
641	XXXXX	0
640	XXXXX	0
639	XXXXX	0
638	XXXXX	0
637	XXXXX	0
636	XXXXX	0
635	XXXXX	0
634	XXXXX	0
633	XXXXX	0
632	XXXXX	0
631	XXXXX	0
630	XXXXX	0
629	XXXXX	0
628	XXXXX	0
627	XXXXX	0
626	XXXXX	0
625	XXXXX	0
624	XXXXX	0
623	XXXXX	0
622	XXXXX	0
621	XXXXX	0
620	XXXXX	0
619	XXXXX	0
618	XXXXX	0
617	XXXXX	0
616	XXXXX	0
615	XXXXX	0
614	XXXXX	0
613	XXXXX	0
612	XXXXX	0
611	XXXXX	0
610	XXXXX	0
609	XXXXX	0
608	XXXXX	0
607	XXXXX	0
606	XXXXX	0
605	XXXXX	0
604	XXXXX	0
603	XXXXX	0
602	XXXXX	0
601	XXXXX	0
600	XXXXX	0
599	XXXXX	0
598	XXXXX	0
597	XXXXX	0
596	XXXXX	0
595	XXXXX	0
594	XXXXX	0

593	XXXXX	0
592	XXXXX	0
591	XXXXX	0
590	XXXXX	0
589	XXXXX	0
588	XXXXX	0
587	XXXXX	0
586	XXXXX	0
585	XXXXX	0
584	XXXXX	0
583	XXXXX	0
582	XXXXX	0
581	XXXXX	0
580	XXXXX	0
579	XXXXX	0
578	XXXXX	0
577	XXXXX	0
576	XXXXX	0
575	XXXXX	0
574	XXXXX	0
573	XXXXX	0
572	XXXXX	0
571	XXXXX	0
570	XXXXX	0
569	XXXXX	0
568	XXXXX	0
567	XXXXX	0
566	XXXXX	0
565	XXXXX	0
564	XXXXX	0
563	XXXXX	0
562	XXXXX	0
561	XXXXX	0
560	XXXXX	0
559	XXXXX	0
558	XXXXX	0
557	XXXXX	0
556	XXXXX	0
555	XXXXX	0
554	XXXXX	0
553	XXXXX	0
552	XXXXX	0
551	XXXXX	0
550	XXXXX	0
549	XXXXX	0
548	XXXXX	0
547	XXXXX	0
546	XXXXX	0
545	XXXXX	0
544	XXXXX	0

543	XXXXX	0
542	XXXXX	0
541	XXXXX	0
540	XXXXX	0
539	XXXXX	0
538	XXXXX	0
537	XXXXX	0
536	XXXXX	0
535	XXXXX	0
534	XXXXX	0
533	XXXXX	0
532	XXXXX	0
531	XXXXX	0
530	XXXXX	0
529	XXXXX	0
528	XXXXX	0
527	XXXXX	0
526	XXXXX	0
525	XXXXX	0
524	XXXXX	0
523	XXXXX	0
522	XXXXX	0
521	XXXXX	0
520	XXXXX	0
519	XXXXX	0
518	XXXXX	0
517	XXXXX	0
516	XXXXX	0
515	XXXXX	0
514	XXXXX	0
513	XXXXX	0
512	XXXXX	0
511	XXXXX	0
510	XXXXX	0
509	XXXXX	0
508	XXXXX	0
507	XXXXX	0
506	XXXXX	0
505	XXXXX	0
504	XXXXX	0
503	XXXXX	0
502	XXXXX	0
501	XXXXX	0
500	XXXXX	0
499	XXXXX	0
498	XXXXX	0
497	XXXXX	0
496	XXXXX	0
495	XXXXX	0
494	XXXXX	0

493	XXXXX	0
492	XXXXX	0
491	XXXXX	0
490	XXXXX	0
489	XXXXX	0
488	XXXXX	0
487	XXXXX	0
486	XXXXX	0
485	XXXXX	0
484	XXXXX	0
483	XXXXX	0
482	XXXXX	0
481	XXXXX	0
480	XXXXX	0
479	XXXXX	0
478	XXXXX	0
477	XXXXX	0
476	XXXXX	0
475	XXXXX	0
474	XXXXX	0
473	XXXXX	0
472	XXXXX	0
471	XXXXX	0
470	XXXXX	0
469	XXXXX	0
468	XXXXX	0
467	XXXXX	0
466	XXXXX	0
465	XXXXX	0
464	XXXXX	0
463	XXXXX	0
462	XXXXX	0
461	XXXXX	0
460	XXXXX	0
459	XXXXX	0
458	XXXXX	0
457	XXXXX	0
456	XXXXX	0
455	XXXXX	0
454	XXXXX	0
453	XXXXX	0
452	XXXXX	0
451	XXXXX	0
450	XXXXX	0
449	XXXXX	0
448	XXXXX	0
447	XXXXX	0
446	XXXXX	0
445	XXXXX	0
444	XXXXX	0

443	XXXXX	0
442	XXXXX	0
441	XXXXX	0
440	XXXXX	0
439	XXXXX	0
438	XXXXX	0
437	XXXXX	0
436	XXXXX	0
435	XXXXX	0
434	XXXXX	0
433	XXXXX	0
432	XXXXX	0
431	XXXXX	0
430	XXXXX	0
429	XXXXX	0
428	XXXXX	0
427	XXXXX	0
426	XXXXX	0
425	XXXXX	0
424	XXXXX	0
423	XXXXX	0
422	XXXXX	0
421	XXXXX	0
420	XXXXX	0
419	XXXXX	0
418	XXXXX	0
417	XXXXX	0
416	XXXXX	0
415	XXXXX	0
414	XXXXX	0
413	XXXXX	0
412	XXXXX	0
411	XXXXX	0
410	XXXXX	0
409	XXXXX	0
408	XXXXX	0
407	XXXXX	0
406	XXXXX	0
405	XXXXX	0
404	XXXXX	0
403	XXXXX	0
402	XXXXX	0
401	XXXXX	0
400	XXXXX	0
399	XXXXX	0
398	XXXXX	0
397	XXXXX	0
396	XXXXX	0
395	XXXXX	0
394	XXXXX	0

393	XXXXX	0
392	XXXXX	0
391	XXXXX	0
390	XXXXX	0
389	XXXXX	0
388	XXXXX	0
387	XXXXX	0
386	XXXXX	0
385	XXXXX	0
384	XXXXX	0
383	XXXXX	0
382	XXXXX	0
381	XXXXX	0
380	XXXXX	0
379	XXXXX	0
378	XXXXX	0
377	XXXXX	0
376	XXXXX	0
375	XXXXX	0
374	XXXXX	0
373	XXXXX	0
372	XXXXX	0
371	XXXXX	0
370	XXXXX	0
369	XXXXX	0
368	XXXXX	0
367	XXXXX	0
366	XXXXX	0
365	XXXXX	0
364	XXXXX	0
363	XXXXX	0
362	XXXXX	0
361	XXXXX	0
360	XXXXX	0
359	XXXXX	0
358	XXXXX	0
357	XXXXX	0
356	XXXXX	0
355	XXXXX	0
354	XXXXX	0
353	XXXXX	0
352	XXXXX	0
351	XXXXX	0
350	XXXXX	0
349	XXXXX	0
348	XXXXX	0
347	XXXXX	0
346	XXXXX	0
345	XXXXX	0
344	XXXXX	0

343	XXXXX	0
342	XXXXX	0
341	XXXXX	0
340	XXXXX	0
339	XXXXX	0
338	XXXXX	0
337	XXXXX	0
336	XXXXX	0
335	XXXXX	0
334	XXXXX	0
333	XXXXX	0
332	XXXXX	0
331	XXXXX	0
330	XXXXX	0
329	XXXXX	0
328	XXXXX	0
327	XXXXX	0
326	XXXXX	0
325	XXXXX	0
324	XXXXX	0
323	XXXXX	0
322	XXXXX	0
321	XXXXX	0
320	XXXXX	0
319	XXXXX	0
318	XXXXX	0
317	XXXXX	0
316	XXXXX	0
315	XXXXX	0
314	XXXXX	0
313	XXXXX	0
312	XXXXX	0
311	XXXXX	0
310	XXXXX	0,123451135
