# Supplementary material for: Comprehensive transcriptomic analysis of prostate cancer lung metastases
Source: PLoS One. 2024 Aug 15;19(8):e0306525. doi: 10.1371/journal.pone.0306525 (PMC11326543; doi:10.1371/journal.pone.0306525)
Supplement: S2 Table — (PDF) [file pone.0306525.s002.pdf]

Probe Nam	Annotation	Accession #	NS Probe II	Class Name	Analyte Typ	Positive Fla	LM	StDev of LN
FOXC2		NM_005251.2		Endogenou	mRNA	FALSCH	5,28	2,06
HLA-DPB1		NM_002121.4		Endogenou	mRNA	FALSCH	1028,91	705,68
PTPRC		NM_080923.2		Endogenou	mRNA	FALSCH	98,99	69,85
ITGB7		NM_000889.1		Endogenou	mRNA	FALSCH	34,02	23,14
CCL5		NM_002985.2		Endogenou	mRNA	FALSCH	180,06	163,53
CCL21		NM_002989.2		Endogenou	mRNA	FALSCH	91,8	179,42
C3		NM_000064.2		Endogenou	mRNA	FALSCH	339,19	746,07
SERPINA1		NM_000295.4		Endogenou	mRNA	FALSCH	214,28	291,78
MFAP4		NM_002404.1		Endogenou	mRNA	FALSCH	551,84	1515,71
ITGAM		NM_000632.3		Endogenou	mRNA	FALSCH	50,44	53,71
ARAP2		NM_015230.2		Endogenou	mRNA	FALSCH	95,75	75,26
PRF1		NM_005041.3		Endogenou	mRNA	FALSCH	102,81	140,07
PDCL3		NM_024065.4		Endogenou	mRNA	FALSCH	146,85	44,27
PRKCB		NM_212535.1		Endogenou	mRNA	FALSCH	47,52	28,94
STAT1		NM_139266.1		Endogenou	mRNA	FALSCH	1059,22	574
CXCR3		NM_001504.1		Endogenou	mRNA	FALSCH	29,28	18,67
PLA2G10		NM_003561.1		Endogenou	mRNA	FALSCH	18,11	33,09
CFP		NM_002621.2		Endogenou	mRNA	FALSCH	15,57	9,39
TWIST1		NM_000474.3		Endogenou	mRNA	FALSCH	22,64	48,22
SPHK2		NM_020126.3		Endogenou	mRNA	FALSCH	132,99	46,28
ADAMTS8		NM_007037.4		Endogenou	mRNA	FALSCH	30,27	81,07
CASP8		NM_001228.4		Endogenou	mRNA	FALSCH	192,75	49,41
HSP90B1		NM_003299.1		Endogenou	mRNA	FALSCH	750,14	220,5
PIK3R2		NM_005027.2		Endogenou	mRNA	FALSCH	200,23	60,59
CHAD		NM_001267.2		Endogenou	mRNA	FALSCH	5,73	4,25
CDK14		NM_012395.2		Endogenou	mRNA	FALSCH	89,95	38,38
S100A14		NM_020672.1		Endogenou	mRNA	FALSCH	24,78	133,07
IBSP		NM_004967.3		Endogenou	mRNA	FALSCH	8,31	35,87
CST7		NM_003650.3		Endogenou	mRNA	FALSCH	37,37	48,89
STAB2		NM_017564.9		Endogenou	mRNA	FALSCH	6,53	4,71
ITGB6		NM_000888.3		Endogenou	mRNA	FALSCH	40,34	84,48
CCL11		NM_002986.2		Endogenou	mRNA	FALSCH	12,74	8,01
TBX4		NM_018488.2		Endogenou	mRNA	FALSCH	31,11	92,79
EPN3		NM_017957.2		Endogenou	mRNA	FALSCH	78,1	60,29
FGL2		NM_006682.2		Endogenou	mRNA	FALSCH	91,24	51,26
EGLN3		NM_022073.3		Endogenou	mRNA	FALSCH	87,12	60,72
TCF3		NM_003200.3		Endogenou	mRNA	FALSCH	59,63	25,25
CXCL10		NM_001565.1		Endogenou	mRNA	FALSCH	114,9	174,35
RPS6KB1		NM_003161.2		Endogenou	mRNA	FALSCH	271,59	56,72
CHRDL1		NM_001143981.1		Endogenou	mRNA	FALSCH	130,99	181,21
CSF2RB		NM_000395.2		Endogenou	mRNA	FALSCH	22,73	15
ALOX5		NM_000698.2		Endogenou	mRNA	FALSCH	361,05	266,25
IL10RA		NM_001558.2		Endogenou	mRNA	FALSCH	138,19	77,03
ZFPM2		NM_012082.3		Endogenou	mRNA	FALSCH	11,63	11,16
FGFR4		NM_002011.3		Endogenou	mRNA	FALSCH	68,03	107,97
GIMAP4		NM_018326.2		Endogenou	mRNA	FALSCH	256,23	241,72
ADD1		NM_001119.4		Endogenou	mRNA	FALSCH	443,73	95,95
ADAM15		NM_207195.1		Endogenou	mRNA	FALSCH	690,91	306,58
CCR2		NM_001123041.2		Endogenou	mRNA	FALSCH	32,47	23,91

TNF	NM_000594.2	Endogenou mRNA	FALSCH	25,8	19,6
KRT7	NM_005556.3	Endogenou mRNA	FALSCH	100,54	833,84
MAPK3	NM_001040056.1	Endogenou mRNA	FALSCH	343,12	109,53
FASLG	NM_000639.1	Endogenou mRNA	FALSCH	10,91	8,51
AAMP	NM_001087.3	Endogenou mRNA	FALSCH	425,14	101,62
CXCL17	NM_198477.1	Endogenou mRNA	FALSCH	76,56	234,87
CHI3L1	NM_001276.2	Endogenou mRNA	FALSCH	67,52	313,86
MTA1	NM_004689.2	Endogenou mRNA	FALSCH	181,62	65,37
FIGF	NM_004469.2	Endogenou mRNA	FALSCH	56,16	219,33
VIT	NM_053276.3	Endogenou mRNA	FALSCH	12,82	4,66
PIK3CD	NM_005026.3	Endogenou mRNA	FALSCH	56,8	36,84
WARS	NM_004184.3	Endogenou mRNA	FALSCH	352,44	232,39
MAPK1	NM_138957.2	Endogenou mRNA	FALSCH	278,08	81,86
TPSB2	NM_024164.5	Endogenou mRNA	FALSCH	447,03	1233,24
SAMSN1	NM_022136.3	Endogenou mRNA	FALSCH	59,27	49,34
CYB561	NM_001915.3	Endogenou mRNA	FALSCH	551,08	208,89
SMAD2	NM_005901.5	Endogenou mRNA	FALSCH	305,68	124,34
TMEM100	NM_018286.2	Endogenou mRNA	FALSCH	66,52	319,34
ADAMTS12	NM_030955.2	Endogenou mRNA	FALSCH	49,63	41,21
MUC1	NM_001018017.1	Endogenou mRNA	FALSCH	325,68	840,58
MAP2K2	NM_030662.3	Endogenou mRNA	FALSCH	233,47	87,23
HAPLN1	NM_001884.3	Endogenou mRNA	FALSCH	5,43	3,42
RPS6KB2	NM_003952.2	Endogenou mRNA	FALSCH	86,67	18,3
GIMAP6	NR_024115.1	Endogenou mRNA	FALSCH	71,6	68,63
FGF2	NM_002006.4	Endogenou mRNA	FALSCH	47,57	76,28
SLC44A4	NM_032794.1	Endogenou mRNA	FALSCH	164,09	211,85
GTF2I	NM_033001.2	Endogenou mRNA	FALSCH	985,74	262,2
AKT1	NM_005163.2	Endogenou mRNA	FALSCH	1363,64	472,47
HKDC1	NM_025130.3	Endogenou mRNA	FALSCH	9,53	35,66
SPARCL1	NM_004684.4	Endogenou mRNA	FALSCH	731,68	850,1
IL18	NM_001562.2	Endogenou mRNA	FALSCH	68,05	116,8
RBM47	NM_019027.3	Endogenou mRNA	FALSCH	339,43	125,97
TBX1	NM_080646.1	Endogenou mRNA	FALSCH	46,01	36,98
CEACAM6	NM_002483.4	Endogenou mRNA	FALSCH	151,75	573,63
IL1B	NM_000576.2	Endogenou mRNA	FALSCH	26,35	32,04
COMP	NM_000095.2	Endogenou mRNA	FALSCH	15,96	54,67
CDC42	NM_001039802.1	Endogenou mRNA	FALSCH	875,11	208,42
VAMP8	NM_003761.3	Endogenou mRNA	FALSCH	167,51	57,64
RAC2	NM_002872.3	Endogenou mRNA	FALSCH	130,74	96,78
PEBP4	NM_144962.2	Endogenou mRNA	FALSCH	44,57	108,69
COL1A1	NM_000088.3	Endogenou mRNA	FALSCH	3309,96	9525,44
MRPS5	NM_031902.3	Housekeep mRNA	FALSCH	255,24	47,36
AGRN	NM_198576.2	Endogenou mRNA	FALSCH	378,8	146,92
KRAS	NM_004985.3	Endogenou mRNA	FALSCH	236,92	52,01
ITM2A	NM_004867.4	Endogenou mRNA	FALSCH	126,19	164,06
MET	NM_001127500.1	Endogenou mRNA	FALSCH	51,43	1093,17
INHBE	NM_031479.3	Endogenou mRNA	FALSCH	5,57	4,4
ANG	NM_001145.4	Endogenou mRNA	FALSCH	90,29	55,62
PPL	NM_002705.4	Endogenou mRNA	FALSCH	148,91	97,2
GZMK	NM_002104.2	Endogenou mRNA	FALSCH	46,88	39,83

ITGA3	NM_002204.2	Endogenou	mRNA	FALSCH	268,37	731,57
PLXNC1	NM_005761.2	Endogenou	mRNA	FALSCH	98,56	70,4
TEK	NM_000459.3	Endogenou	mRNA	FALSCH	49,15	95,76
ITGB2	NM_001127491.1	Endogenou	mRNA	FALSCH	243,03	183,98
SF3A3	NM_006802.2	Housekeep	mRNA	FALSCH	257,9	41,54
VEZF1	NM_007146.2	Endogenou	mRNA	FALSCH	194,65	53,21
CCL7	NM_006273.2	Endogenou	mRNA	FALSCH	5,02	2,68
FBLN5	NM_006329.3	Endogenou	mRNA	FALSCH	132,17	192,26
PLA2G2D	NM_001271814.1	Endogenou	mRNA	FALSCH	11,32	14,69
TNFSF10	NM_003810.2	Endogenou	mRNA	FALSCH	817,35	474,48
CYBB	NM_000397.3	Endogenou	mRNA	FALSCH	278,23	375,34
CAV1	NM_001753.3	Endogenou	mRNA	FALSCH	320,48	885,77
GDF5	NM_000557.2	Endogenou	mRNA	FALSCH	5,69	5,48
SSTR2	NM_001050.2	Endogenou	mRNA	FALSCH	7,64	8,44
COL4A1	NM_001845.4	Endogenou	mRNA	FALSCH	842,94	522,2
SLC12A6	NM_001042494.1	Endogenou	mRNA	FALSCH	73,05	39,81
MRC1	NM_002438.2	Endogenou	mRNA	FALSCH	238,78	263,94
SET	NM_001122821.1	Endogenou	mRNA	FALSCH	385,84	80,71
MYC	NM_002467.3	Endogenou	mRNA	FALSCH	457,59	567,11
OLFML2B	NM_015441.1	Endogenou	mRNA	FALSCH	80,98	69,79
MYH11	NM_001040113.1	Endogenou	mRNA	FALSCH	145,45	317,14
PIK3CG	NM_002649.2	Endogenou	mRNA	FALSCH	66,71	44,8
PPP2CB	NM_001009552.1	Endogenou	mRNA	FALSCH	621,73	284,92
AHNAK	NM_001620.2	Endogenou	mRNA	FALSCH	1819,9	1098,18
SRGN	NR_036430.1	Endogenou	mRNA	FALSCH	764,63	1189,85
CCL8	NM_005623.2	Endogenou	mRNA	FALSCH	18,82	28,04
CCBE1	NM_133459.3	Endogenou	mRNA	FALSCH	33,57	74,34
LAMB3	NM_000228.2	Endogenou	mRNA	FALSCH	43,38	199,63
KRT19	NM_002276.4	Endogenou	mRNA	FALSCH	497,17	735,17
TCEB2	NM_007108.2	Endogenou	mRNA	FALSCH	28,08	13,97
GLYR1	NM_032569.3	Endogenou	mRNA	FALSCH	295,43	50,15
PTGDS	NM_000954.5	Endogenou	mRNA	FALSCH	233,41	528,69
IL1RL1	NM_016232.4	Endogenou	mRNA	FALSCH	28,95	82,84
SLPI	NM_003064.2	Endogenou	mRNA	FALSCH	356,2	542,92
IL1A	NM_000575.3	Endogenou	mRNA	FALSCH	7,63	5,61
CSPG4	NM_001897.4	Endogenou	mRNA	FALSCH	77,65	76,91
ACTG2	NM_001615.3	Endogenou	mRNA	FALSCH	72,91	96,07
TNS1	NM_022648.4	Endogenou	mRNA	FALSCH	482,05	696,09
EDC3	NM_001142443.1	Housekeep	mRNA	FALSCH	195,35	38,76
ITGA8	NM_003638.1	Endogenou	mRNA	FALSCH	46,34	109,29
ANGPT1	NM_001146.3	Endogenou	mRNA	FALSCH	95,8	221,98
CD82	NM_002231.3	Endogenou	mRNA	FALSCH	132,23	77,87
C3AR1	NM_004054.2	Endogenou	mRNA	FALSCH	99,3	101,21
CTSH	NM_148979.2	Endogenou	mRNA	FALSCH	504,52	526,77
COL1A2	NM_000089.3	Endogenou	mRNA	FALSCH	1373,26	2433,92
EPHB3	NM_004443.3	Endogenou	mRNA	FALSCH	73,46	70,5
MMP9	NM_004994.2	Endogenou	mRNA	FALSCH	88,51	432,96
PTK2B	NM_004103.3	Endogenou	mRNA	FALSCH	143,36	103,88
TFPI2	NM_006528.3	Endogenou	mRNA	FALSCH	26,24	176,26
EIF2AK3	NM_004836.5	Endogenou	mRNA	FALSCH	155,3	50,93

EDN1	NM_001955.2	Endogenou	mRNA	FALSCH	84,32	115,98
RB1	NM_000321.1	Endogenou	mRNA	FALSCH	163,23	86,61
ACVRL1	NM_000020.1	Endogenou	mRNA	FALSCH	187,05	348,84
IFNG	NM_000619.2	Endogenou	mRNA	FALSCH	10,19	10,25
CUL1	NM_003592.2	Endogenou	mRNA	FALSCH	206	32,74
PIK3R5	NM_001142633.1	Endogenou	mRNA	FALSCH	46,17	28,18
C1S	NM_001734.2	Endogenou	mRNA	FALSCH	331,32	313,51
EPAS1	NM_001430.3	Endogenou	mRNA	FALSCH	508,42	1033,3
ICAM1	NM_000201.2	Endogenou	mRNA	FALSCH	238,66	294,49
SERPING1	NM_000062.2	Endogenou	mRNA	FALSCH	716,11	668,99
ADAM28	NM_014265.4	Endogenou	mRNA	FALSCH	48,4	72,21
CYP1B1	NM_000104.3	Endogenou	mRNA	FALSCH	67,52	142,77
TYMP	NM_001953.3	Endogenou	mRNA	FALSCH	256,54	194,89
RRAS	NM_006270.3	Endogenou	mRNA	FALSCH	101,6	93,88
SPARC	NM_003118.2	Endogenou	mRNA	FALSCH	2333,64	2433,37
PCOLCE	NM_002593.3	Endogenou	mRNA	FALSCH	199,73	221,96
CXCL11	NM_005409.3	Endogenou	mRNA	FALSCH	34,09	123,54
NOL7	NM_016167.3	Housekeep	mRNA	FALSCH	269,57	95,64
SEMA3E	NM_012431.1	Endogenou	mRNA	FALSCH	51,73	87,11
PTPRB	NM_002837.3	Endogenou	mRNA	FALSCH	115,27	163,9
BNC2	NM_017637.5	Endogenou	mRNA	FALSCH	17,06	12,16
KIAA1462	NM_020848.2	Endogenou	mRNA	FALSCH	110,94	142,69
EVI2A	NM_014210.3	Endogenou	mRNA	FALSCH	61,04	31,86
ERCC3	NM_000122.1	Housekeep	mRNA	FALSCH	82,13	17,52
ILK	NM_004517.2	Endogenou	mRNA	FALSCH	214,36	90,42
NCAM1	NM_000615.5	Endogenou	mRNA	FALSCH	16,59	9,87
BAD	NM_004322.3	Endogenou	mRNA	FALSCH	90,74	28,7
BMPR1B	NM_001203.1	Endogenou	mRNA	FALSCH	202,38	413,83
SERPINH1	NM_001235.2	Endogenou	mRNA	FALSCH	707,31	520,15
AKAP12	NM_005100.3	Endogenou	mRNA	FALSCH	256,82	205,83
FSTL1	NM_007085.4	Endogenou	mRNA	FALSCH	1231,16	1004,11
ROCK2	NM_004850.3	Endogenou	mRNA	FALSCH	395,87	120,22
GJA5	NM_005266.5	Endogenou	mRNA	FALSCH	42,61	67,19
USP39	NM_001256725.1	Housekeep	mRNA	FALSCH	102,47	23,64
NME4	NM_005009.2	Endogenou	mRNA	FALSCH	335,01	198,23
CNOT10	NM_001256741.1	Housekeep	mRNA	FALSCH	190,62	38,08
ANXA2P2	NR_003573.1	Endogenou	mRNA	FALSCH	1804,65	1662,9
NAA15	NM_057175.3	Endogenou	mRNA	FALSCH	97	18,42
KRT14	NM_000526.4	Endogenou	mRNA	FALSCH	9,44	67,35
PDPN	NM_006474.4	Endogenou	mRNA	FALSCH	79,44	107,15
PTPRM	NM_002845.3	Endogenou	mRNA	FALSCH	260,83	192,75
SCNN1A	NM_001038.4	Endogenou	mRNA	FALSCH	185,65	197,21
STAB1	NM_015136.2	Endogenou	mRNA	FALSCH	91,85	113,25
RORB	NM_006914.3	Endogenou	mRNA	FALSCH	16,68	81,49
BRMS1	NM_015399.3	Endogenou	mRNA	FALSCH	112,84	21,74
VCAM1	NM_001078.3	Endogenou	mRNA	FALSCH	91,11	431,6
WIPF1	NM_001077269.1	Endogenou	mRNA	FALSCH	129,29	66,39
HEG1	NM_020733.1	Endogenou	mRNA	FALSCH	208	221,86
CTSL	NM_001912.4	Endogenou	mRNA	FALSCH	272,9	957,58
CXCL13	NM_006419.2	Endogenou	mRNA	FALSCH	18,6	24,83

PECAM1	NM_000442.3	Endogenou	mRNA	FALSCH	618,47	831,08
EMCN	NM_016242.3	Endogenou	mRNA	FALSCH	76,82	130,33
NFKB1	NM_003998.2	Endogenou	mRNA	FALSCH	43,74	20,57
SP1	NM_003109.1	Endogenou	mRNA	FALSCH	180,19	35,65
CLEC2B	NM_005127.2	Endogenou	mRNA	FALSCH	262,74	212,78
PLCG2	NM_002661.2	Endogenou	mRNA	FALSCH	56,96	40,98
LAMC1	NM_002293.3	Endogenou	mRNA	FALSCH	402,68	252,36
COL4A2	NM_001846.2	Endogenou	mRNA	FALSCH	754,52	442,67
SV2B	NM_001167580.1	Endogenou	mRNA	FALSCH	19,8	91,37
JUN	NM_002228.3	Endogenou	mRNA	FALSCH	641,56	596,98
KDM1A	NM_015013.3	Endogenou	mRNA	FALSCH	169,43	50,99
PPP1R16B	NM_015568.2	Endogenou	mRNA	FALSCH	33,72	28,47
RBPJ	NM_015874.3	Endogenou	mRNA	FALSCH	338,84	83,27
MMP13	NM_002427.2	Endogenou	mRNA	FALSCH	6,18	74,23
DHX16	NM_001164239.1	Housekeep	mRNA	FALSCH	118,44	17,46
IL6	NM_000600.1	Endogenou	mRNA	FALSCH	13,67	12,89
ITGA1	NM_181501.1	Endogenou	mRNA	FALSCH	258,85	186,88
PDGFC	NM_016205.2	Endogenou	mRNA	FALSCH	44,61	100,47
ZFYVE16	NM_001105251.2	Endogenou	mRNA	FALSCH	198,62	42,94
TJP3	NM_014428.1	Endogenou	mRNA	FALSCH	84,43	138,12
SNAI3	NM_178310.1	Endogenou	mRNA	FALSCH	12,68	6,98
HK3	NM_002115.1	Endogenou	mRNA	FALSCH	20,69	21,42
IL15	NM_172174.1	Endogenou	mRNA	FALSCH	28,52	21,51
COG7	NM_153603.3	Housekeep	mRNA	FALSCH	244,64	35,16
LAMC2	NM_005562.2	Endogenou	mRNA	FALSCH	49,55	160,87
VAV2	NM_003371.3	Endogenou	mRNA	FALSCH	87,64	23,01
TGFBR2	NM_001024847.1	Endogenou	mRNA	FALSCH	425,39	444,23
HOXA5	NM_019102.2	Endogenou	mRNA	FALSCH	53,82	44,7
CLDN3	NM_001306.3	Endogenou	mRNA	FALSCH	396,95	383,52
FLI1	NM_001167681.2	Endogenou	mRNA	FALSCH	89,38	75,98
NUBP1	NM_001278506.1	Housekeep	mRNA	FALSCH	47,12	9,97
TNFSF12	NM_003809.2	Endogenou	mRNA	FALSCH	189,67	96,52
CXADR	NM_001338.3	Endogenou	mRNA	FALSCH	248,99	186,94
COL3A1	NM_000090.3	Endogenou	mRNA	FALSCH	3822,99	8459,5
BGN	NM_001711.3	Endogenou	mRNA	FALSCH	460,43	368,79
LY96	NM_015364.2	Endogenou	mRNA	FALSCH	67,81	58,85
ADM2	NM_001253845.1	Endogenou	mRNA	FALSCH	30,78	43,64
HSPG2	NM_005529.5	Endogenou	mRNA	FALSCH	872,97	491,94
COL5A2	NM_000393.3	Endogenou	mRNA	FALSCH	250,01	446,5
DPYSL3	NM_001387.2	Endogenou	mRNA	FALSCH	338,16	168,51
TMPRSS6	NM_153609.2	Endogenou	mRNA	FALSCH	4,67	0,78
FREM2	NM_207361.4	Endogenou	mRNA	FALSCH	15,62	29,16
MTMR14	NM_022485.3	Housekeep	mRNA	FALSCH	216,13	42,59
PTRF	NM_012232.5	Endogenou	mRNA	FALSCH	42,74	39,52
ITGB4	NM_001005731.1	Endogenou	mRNA	FALSCH	115,48	318,63
MYO1D	NM_015194.1	Endogenou	mRNA	FALSCH	263,7	165,75
ABI3BP	NM_015429.3	Endogenou	mRNA	FALSCH	234,4	351,83
RBL1	NM_183404.1	Endogenou	mRNA	FALSCH	31,37	11,72
NR3C1	NM_001018077.1	Endogenou	mRNA	FALSCH	378,23	204,59
PPP2R1A	NM_014225.3	Endogenou	mRNA	FALSCH	805,76	202,83

TNXB	NM_032470.3	Endogenou	mRNA	FALSCH	52,99	111,44
AP1M2	NM_005498.4	Endogenou	mRNA	FALSCH	159,95	77,46
NRXN1	NM_138735.2	Endogenou	mRNA	FALSCH	8,01	4,16
PYCARD	NM_013258.3	Endogenou	mRNA	FALSCH	33,61	26,66
CALCRL	NM_005795.3	Endogenou	mRNA	FALSCH	267,04	573,57
ITGA6	NM_000210.1	Endogenou	mRNA	FALSCH	253,51	89,97
PTX3	NM_002852.3	Endogenou	mRNA	FALSCH	13,61	14,21
SYK	NM_003177.3	Endogenou	mRNA	FALSCH	134,55	68,86
SPDEF	NM_012391.1	Endogenou	mRNA	FALSCH	658,7	1077,28
PROK2	NM_021935.3	Endogenou	mRNA	FALSCH	9,5	9,31
SYNE1	NM_015293.1	Endogenou	mRNA	FALSCH	83,68	115,32
EGFR	NM_201282.1	Endogenou	mRNA	FALSCH	414,08	372,57
ID2	NM_002166.4	Endogenou	mRNA	FALSCH	135,58	103,62
HOXB13	NM_006361.5	Endogenou	mRNA	FALSCH	336,52	691,37
CD36	NM_000072.3	Endogenou	mRNA	FALSCH	247,88	362,15
OAS1	NM_001032409.1	Endogenou	mRNA	FALSCH	178,98	812,59
PLXDC1	NM_020405.4	Endogenou	mRNA	FALSCH	51,39	42,06
GPATCH3	NM_022078.2	Housekeep	mRNA	FALSCH	36,2	12,81
IL13RA2	NM_000640.2	Endogenou	mRNA	FALSCH	10,04	30,69
NRXN3	NM_001105250.1	Endogenou	mRNA	FALSCH	39,71	96,09
MISP	NM_173481.2	Endogenou	mRNA	FALSCH	30,03	223,57
TLK2	NM_006852.2	Housekeep	mRNA	FALSCH	207,48	36,09
WNT5B	NM_032642.2	Endogenou	mRNA	FALSCH	37,52	27,68
ITGA2	NM_002203.2	Endogenou	mRNA	FALSCH	75,15	54,47
TNFRSF1A	NM_001065.2	Endogenou	mRNA	FALSCH	436,1	230,17
RBX1	NM_014248.2	Endogenou	mRNA	FALSCH	579,75	133,23
LTBP4	NM_003573.2	Endogenou	mRNA	FALSCH	417,98	632,62
RGCC	NM_014059.2	Endogenou	mRNA	FALSCH	1008,2	2223,71
COL18A1	NM_030582.3	Endogenou	mRNA	FALSCH	379,1	283,18
HSPB1	NM_001540.3	Endogenou	mRNA	FALSCH	654,7	468,32
PTK6	NM_005975.2	Endogenou	mRNA	FALSCH	41,56	71,03
ATPIF1	NM_178190.2	Endogenou	mRNA	FALSCH	635,32	232,27
HDAC3	NM_003883.2	Housekeep	mRNA	FALSCH	154,82	34,5
ITGB3	NM_000212.2	Endogenou	mRNA	FALSCH	19,66	215,61
CDH2	NM_001792.3	Endogenou	mRNA	FALSCH	18,25	78,22
ITGB1	NM_033666.2	Endogenou	mRNA	FALSCH	1421,62	1221,35
VAV3	NM_001079874.1	Endogenou	mRNA	FALSCH	30,82	52
ZC3H14	NM_001160103.1	Housekeep	mRNA	FALSCH	282,15	76,45
CTSK	NM_000396.2	Endogenou	mRNA	FALSCH	335,71	314,1
PROM1	NM_006017.1	Endogenou	mRNA	FALSCH	8,88	20,01
RTN4	NM_007008.2	Endogenou	mRNA	FALSCH	1163,11	534,89
GDF15	NM_004864.2	Endogenou	mRNA	FALSCH	247,82	325,61
FBP1	NM_000507.3	Endogenou	mRNA	FALSCH	567,94	352,65
SNAI1	NM_005985.2	Endogenou	mRNA	FALSCH	26,07	21,1
VWA2	NM_001272046.1	Endogenou	mRNA	FALSCH	33,59	27,33
ESRP1	NM_001034915.2	Endogenou	mRNA	FALSCH	467,3	282,23
PMP22	NM_000304.2	Endogenou	mRNA	FALSCH	179,37	190,38
MYLK	NM_053032.2	Endogenou	mRNA	FALSCH	584,77	526,84
ITGA11	NM_012211.3	Endogenou	mRNA	FALSCH	60,31	73,39
FGFR1	NM_015850.2	Endogenou	mRNA	FALSCH	188,85	124,73

CD46	NM_172350.1	Endogenou	mRNA	FALSCH	1374,41	349,08
RUNX1	NM_001754.4	Endogenou	mRNA	FALSCH	107,56	64,16
THY1	NM_006288.2	Endogenou	mRNA	FALSCH	137,1	197,67
RAMP2	NM_005854.2	Endogenou	mRNA	FALSCH	129,22	200,6
VWA1	NM_199121.2	Endogenou	mRNA	FALSCH	21,81	27,54
SLIT2	NM_004787.1	Endogenou	mRNA	FALSCH	115,44	162,62
SRF	NM_003131.2	Endogenou	mRNA	FALSCH	252,27	57,85
TJP2	NM_004817.2	Endogenou	mRNA	FALSCH	109,33	48,72
EPHA1	NM_005232.3	Endogenou	mRNA	FALSCH	48,14	30,58
CREBBP	NM_001079846.1	Endogenou	mRNA	FALSCH	225,91	45,63

Reference	StDev of Re	LM vs. Reference	P value of: LM vs.	***FDR***	***FDR****
35,57	56,8	-6,74	0,00000001	**2,56667E-06**	**2,56667E-06**
196,08	133,65	5,25	0,00000001	**0,00000385**	**0,00000385**
16,06	22,83	6,16	0,00000001	**0,0000077**	**0,0000077**
6,02	7,82	5,65	0,00000005	**0,000009625**	**2,56667E-06**
29,71	40,81	6,06	0,00000023	**0,0000253**	**0,0000253**
6,37	28,76	14,42	0,0000002	**2,56667E-05**	**2,56667E-05**
21,48	31,69	15,79	0,00000017	**0,00002618**	**0,000009625**
27,78	67,71	7,71	0,00000171	**0,00013167**	**0,00013167**
27,42	80,55	20,12	0,00000143	**0,000137638**	**0,0000253**
12,02	14,81	4,2	0,00000163	**0,000139456**	**0,000137638**
20,85	20,94	4,59	0,00000271	**0,0001897**	**0,00013167**
18,77	32,32	5,48	0,00000353	**0,000226508**	**0,0001897**
266,23	122,99	-1,81	0,00000491	**0,000290823**	**0,000226508**
12,25	16,03	3,88	0,00000829	**0,000375488**	**0,000375488**
380,94	488,94	2,78	0,00000788	**0,000379225**	**0,000379225**
7,16	11,23	4,09	0,00000893	**0,000382006**	**0,000375488**
3,91	3,82	4,63	0,0000095	**0,000385**	**0,000382006**
4,37	7,48	3,56	0,00000704	**0,0003872**	**0,000290823**
141,99	262,67	-6,27	0,00000777	**0,00039886**	**0,0003872**
236,43	89,4	-1,78	0,00001079	**0,000415415**	**0,000385**
3,35	4,25	9,03	0,00001146	**0,0004202**	**0,000415415**
101,84	48,83	1,89	0,00001752	**0,000465186**	**0,000465186**
1414,34	1054,95	-1,89	0,00001713	**0,000471075**	**0,000471075**
333,72	136,57	-1,67	0,0000171	**0,000487667**	**0,000487667**
112,18	1861,86	-19,57	0,00001659	**0,000491319**	**0,000491319**
180,08	83,71	-2	0,00001545	**0,000495688**	**0,000495688**
3,84	4,37	6,46	0,00001625	**0,0005005**	**0,000495688**
177,43	2624,01	-21,35	0,00001969	**0,000505377**	**0,000465186**
7,4	12,69	5,05	0,00001449	**0,00050715**	**0,0004202**
2,56	2,2	2,55	0,00001529	**0,000511883**	**0,00050715**
4,81	6,94	8,39	0,00002302	**0,000571787**	**0,000505377**
4,39	5	2,9	0,0000265	**0,000637656**	**0,000571787**
3,32	3,04	9,38	0,00003168	**0,000717459**	**0,000717459**
258,94	208,53	-3,32	0,00003311	**0,00072842**	**0,000717459**
28,89	46,44	3,16	0,00003139	**0,000732433**	**0,000637656**
28,56	38,99	3,05	0,00003559	**0,000761231**	**0,00072842**
110,84	47,46	-1,86	0,00003742	**0,000778741**	**0,000761231**
18,91	32,18	6,08	0,00004622	**0,000936563**	**0,000778741**
391,79	129,54	-1,44	0,00005068	**0,001000605**	**0,000936563**
21,06	44,57	6,22	0,00005545	**0,001067413**	**0,001000605**
6,81	10,7	3,34	0,00006071	**0,001113017**	**0,001113017**
115,75	134,69	3,12	0,00005985	**0,001124012**	**0,001067413**
53,23	44,25	2,6	0,00006305	**0,001129035**	**0,001113017**
3,85	2,8	3,02	0,00007607	**0,00122029**	**0,00122029**
15,08	19,72	4,51	0,00007475	**0,001224628**	**0,001224628**
90,77	50,89	2,82	0,00007073	**0,001237775**	**0,001129035**
313,76	87,75	1,41	0,00007436	**0,001244722**	**0,001244722**
1287,45	619,98	-1,86	0,0000735	**0,001257667**	**0,001237775**
10,6	11,66	3,06	0,00008321	**0,001307586**	**0,00122029**

7,92	11,05	3,26	0,00008929	**0,001348104**	**0,001348104**
9,17	28,51	10,96	0,00008764	**0,001349656**	**0,001307586**
228,04	58,18	1,5	0,00009227	**0,001366306**	**0,001348104**
3,72	4,65	2,93	0,00009761	**0,001418108**	**0,001366306**
638,58	315,25	-1,5	0,00009978	**0,001422789**	**0,001418108**
8,29	102,12	9,24	0,00010581	**0,00148134**	**0,001422789**
11,75	33	5,75	0,00011156	**0,001507039**	**0,001507039**
304,39	184,92	-1,68	0,00011439	**0,001518626**	**0,001507039**
5,57	10,85	10,09	0,00011087	**0,001524463**	**0,00148134**
31,87	43,49	-2,49	0,00013854	**0,001808064**	**0,001518626**
19,73	39,33	2,88	0,00015188	**0,001917174**	**0,001917174**
151,89	63,14	2,32	0,00014977	**0,001922048**	**0,001808064**
150,57	63,85	1,85	0,00015916	**0,001945289**	**0,001945289**
71,55	113,22	6,25	0,0001582	**0,001964742**	**0,001917174**
25,67	19	2,31	0,00016831	**0,00202498**	**0,001945289**
1004,37	845,18	-1,82	0,00018216	**0,002157895**	**0,00202498**
190,15	71,05	1,61	0,00018505	**0,002158917**	**0,002157895**
6,86	7,34	9,7	0,00018965	**0,00217956**	**0,002158917**
16,98	18,93	2,92	0,00022028	**0,002494347**	**0,00217956**
41,97	534,07	7,76	0,00022563	**0,0025179**	**0,002494347**
370,32	145,04	-1,59	0,00024944	**0,002667622**	**0,002667622**
17,92	42,62	-3,3	0,00024873	**0,002697494**	**0,002697494**
127,11	62,31	-1,47	0,00024832	**0,00273152**	**0,0025179**
23,35	27,37	3,07	0,00026232	**0,002766937**	**0,002667622**
12	14,19	3,96	0,00026614	**0,002769295**	**0,002766937**
738,43	1079,6	-4,5	0,00029465	**0,0029465**	**0,0029465**
1397,17	428,27	-1,42	0,0002915	**0,002953355**	**0,002953355**
2192,79	869,39	-1,61	0,00028897	**0,002966759**	**0,002769295**
2,7	2,26	3,53	0,00031332	**0,003093031**	**0,0029465**
234,71	299,69	3,12	0,00031907	**0,003109923**	**0,003093031**
22,72	20,39	3	0,00032344	**0,00311311**	**0,003109923**
207,95	87,16	1,63	0,00032925	**0,003129907**	**0,00311311**
106,34	49,95	-2,31	0,00033733	**0,003167611**	**0,003129907**
16,98	38,12	8,94	0,00039775	**0,003646042**	**0,003646042**
7,53	17,28	3,5	0,00039398	**0,003654995**	**0,003167611**
77,42	216,77	-4,85	0,00042834	**0,003880256**	**0,003646042**
633,19	192,43	1,38	0,00046592	**0,004171609**	**0,003880256**
80,73	77,87	2,07	0,00047903	**0,004239691**	**0,004171609**
49,78	59,26	2,63	0,00051408	**0,0044982**	**0,004239691**
7,55	17,42	5,91	0,0005225	**0,004520506**	**0,0044982**
18420,2	77878,02	-5,57	0,00055053	**0,00471009**	**0,004520506**
188,24	59,96	1,36	0,00056803	**0,004806408**	**0,00471009**
685,53	507,3	-1,81	0,0006058	**0,005070283**	**0,004806408**
341,28	145,68	-1,44	0,00062299	**0,005158089**	**0,005070283**
37,87	38,62	3,33	0,00063028	**0,005162932**	**0,005158089**
8,36	18,27	6,15	0,00063878	**0,00517748**	**0,005162932**
12,52	16,57	-2,25	0,00068601	**0,005282277**	**0,005282277**
33,4	48,3	2,7	0,00065933	**0,005288376**	**0,00517748**
52,5	78,44	2,84	0,00067862	**0,005332014**	**0,005332014**
15,53	21,88	3,02	0,00068601	**0,005335633**	**0,005332014**

79,26	46,58	3,39	0,00067295	**0,005341974**	**0,005288376**
38,28	45,55	2,57	0,00073629	**0,005558268**	**0,005558268**
12,84	19,41	3,83	0,00072969	**0,005562983**	**0,005282277**
101,54	115,08	2,39	0,00074591	**0,00557622**	**0,005558268**
368,48	167,2	-1,43	0,00079878	**0,005914044**	**0,00557622**
136,38	46,14	1,43	0,00080865	**0,0059301**	**0,005914044**
2,76	2,52	1,82	0,00086114	**0,006255451**	**0,0059301**
41,08	37,34	3,22	0,00094063	**0,00676902**	**0,006255451**
4,12	7,82	2,75	0,00097912	**0,006980763**	**0,00676902**
326,58	709,04	2,5	0,00099046	**0,006996828**	**0,006980763**
115,85	111	2,4	0,00100899	**0,00706293**	**0,006996828**
76,06	42,35	4,21	0,00105959	**0,007350309**	**0,00706293**
2,93	2,1	1,94	0,00107701	**0,007404444**	**0,007350309**
18,97	24,95	-2,48	0,001136	**0,007672982**	**0,007672982**
1799,99	1739,37	-2,14	0,00112784	**0,007685281**	**0,007404444**
41,6	21,71	1,76	0,00120756	**0,0080157**	**0,0080157**
78,85	72,29	3,03	0,00120135	**0,008043822**	**0,007672982**
522,49	194,66	-1,35	0,00123666	**0,008138703**	**0,0080157**
1089,13	915,49	-2,38	0,00130102	**0,008489707**	**0,008138703**
200,57	252,93	-2,48	0,00132177	**0,008552629**	**0,008489707**
30,93	500,84	4,7	0,00135687	**0,008706583**	**0,008552629**
30,7	33,02	2,17	0,0014834	**0,009439818**	**0,008706583**
384,8	153,42	1,62	0,00154397	**0,009744729**	**0,009439818**
1042,53	522,54	1,75	0,00158462	**0,009919979**	**0,009744729**
302,26	397,21	2,53	0,00163612	**0,010159777**	**0,009919979**
6,62	8,52	2,84	0,00168263	**0,010365001**	**0,010159777**
7,75	34,31	4,33	0,00173661	**0,010612617**	**0,010365001**
8,92	14,07	4,86	0,00184889	**0,011209806**	**0,010612617**
127,27	388,52	3,91	0,00189282	**0,011298228**	**0,011298228**
47,15	26,58	-1,68	0,00192914	**0,01133922**	**0,01133922**
202,27	78,96	1,46	0,00191885	**0,011365496**	**0,011298228**
49,08	109,53	4,76	0,00188991	**0,01136899**	**0,011209806**
8,27	6,85	3,5	0,00197852	**0,011541367**	**0,01133922**
67,67	814,84	5,26	0,00199663	**0,011559437**	**0,011541367**
3,59	10,76	2,12	0,00204631	**0,011671546**	**0,011671546**
197,05	236,48	-2,54	0,00203571	**0,011697737**	**0,011559437**
22,11	242,08	3,3	0,00216287	**0,012156277**	**0,012156277**
187,79	150,14	2,57	0,0021553	**0,012202801**	**0,011671546**
253,84	72,8	-1,3	0,00230199	**0,012844437**	**0,012156277**
13,62	19,06	3,4	0,00241043	**0,013070642**	**0,013070642**
28,89	26,64	3,32	0,00239768	**0,013093713**	**0,013093713**
74,34	39,51	1,78	0,00239121	**0,013151655**	**0,013151655**
47,3	46,34	2,1	0,00237911	**0,013179242**	**0,012844437**
223,06	109,39	2,26	0,00259778	**0,013988046**	**0,013070642**
4965,81	18502,88	-3,62	0,00263667	**0,014001627**	**0,014001627**
134,76	66,55	-1,83	0,0026355	**0,014092604**	**0,013988046**
462,07	1905,24	-5,22	0,00271044	**0,014294786**	**0,014001627**
78,93	57,78	1,82	0,00276641	**0,014490719**	**0,014294786**
6,55	17,82	4	0,00284184	**0,014785249**	**0,014490719**
218,02	80	-1,4	0,00286438	**0,014802501**	**0,014785249**

27,82	60,83	3,03	0,00292496	**0,015014795**	**0,014802501**
94,21	33,79	1,73	0,00297603	**0,015175782**	**0,015014795**
62,27	47,64	3	0,00317744	**0,016096242**	**0,015175782**
4,65	4,84	2,19	0,00327078	**0,016460788**	**0,016096242**
169,35	37,94	1,22	0,00330505	**0,01652525**	**0,016460788**
24,62	22,18	1,88	0,00333202	**0,016552615**	**0,01652525**
123,78	120,72	2,68	0,00336345	**0,016601644**	**0,016552615**
170	95,39	2,99	0,00345452	**0,01694255**	**0,016601644**
81,53	153,16	2,93	0,00353704	**0,017237473**	**0,01694255**
271,41	224,19	2,64	0,00362636	**0,017561618**	**0,017237473**
23,69	13,13	2,04	0,00369207	**0,017657726**	**0,017657726**
158,04	259,57	-2,34	0,00368439	**0,017731127**	**0,017561618**
110,94	185,48	2,31	0,00378107	**0,017861496**	**0,017861496**
51,9	34,08	1,96	0,00375888	**0,017866281**	**0,017657726**
5954,15	14151,77	-2,55	0,00390213	**0,018320976**	**0,017861496**
509,37	1042,93	-2,55	0,00404359	**0,018870087**	**0,018320976**
8,64	28,21	3,95	0,0042117	**0,019536199**	**0,018870087**
179,45	84,34	1,5	0,00429227	**0,019790706**	**0,019536199**
15,33	44,48	3,38	0,00447578	**0,020513992**	**0,019790706**
45,19	27,21	2,55	0,00465935	**0,021228991**	**0,020513992**
7,29	12,38	2,34	0,00476859	**0,021598908**	**0,021228991**
50,04	24,55	2,22	0,00490562	**0,022089634**	**0,021598908**
31,31	40,49	1,95	0,00512669	**0,02295088**	**0,022089634**
109,62	48,21	-1,33	0,00519174	**0,023107745**	**0,02295088**
138,15	79,52	1,55	0,00525842	**0,02327002**	**0,023107745**
45,73	154,08	-2,76	0,00613292	**0,026984848**	**0,02327002**
133,37	94,7	-1,47	0,00626655	**0,027416156**	**0,026984848**
627,87	843,19	-3,1	0,00648039	**0,028191527**	**0,027416156**
1285,52	1600,93	-1,82	0,00659916	**0,028546928**	**0,028191527**
123,63	180,54	2,08	0,00687859	**0,029425079**	**0,029425079**
2221,89	1815,21	-1,8	0,00687294	**0,029565161**	**0,028546928**
293,95	115,98	1,35	0,00701029	**0,029822781**	**0,029425079**
16,14	12,67	2,64	0,00748111	**0,031477895**	**0,031477895**
57,43	34,94	1,78	0,00747922	**0,031642854**	**0,029822781**
523,5	264,75	-1,56	0,00778883	**0,032244081**	**0,032244081**
144,09	51,42	1,32	0,00773311	**0,032361384**	**0,031477895**
896,75	989,46	2,01	0,00778072	**0,032384618**	**0,032361384**
127,68	74,96	-1,32	0,00815052	**0,033560965**	**0,032244081**
3,45	7,65	2,74	0,00833289	**0,03412939**	**0,033560965**
30,33	39,63	2,62	0,00845174	**0,034433015**	**0,03412939**
149,39	74,42	1,75	0,0085268	**0,034555979**	**0,034433015**
56,5	162,74	3,29	0,00878967	**0,035434795**	**0,034555979**
177,03	127,69	-1,93	0,00908918	**0,036262532**	**0,036262532**
65,07	373,43	-3,9	0,00904712	**0,036282721**	**0,035434795**
142,06	55,47	-1,26	0,00914987	**0,036316494**	**0,036262532**
34,94	53	2,61	0,00944882	**0,037310725**	**0,036316494**
73,41	61,35	1,76	0,00990462	**0,038713489**	**0,038713489**
102,1	72,37	2,04	0,00988299	**0,038826032**	**0,037310725**
135,96	57,97	2,01	0,01007577	**0,03918355**	**0,038713489**
7,6	13,78	2,45	0,01035644	**0,040072657**	**0,03918355**

291,85	192,63	2,12	0,01053399	**0,040555862**	**0,040072657**
31,73	28,5	2,42	0,01075668	**0,041207182**	**0,040555862**
29,66	12,49	1,47	0,01086972	**0,041434081**	**0,041207182**
217,61	51,24	-1,21	0,01149878	**0,043616062**	**0,041434081**
141,31	113,99	1,86	0,01165482	**0,043991232**	**0,043616062**
30,85	25,21	1,85	0,01176079	**0,044174675**	**0,043991232**
616,24	277,47	-1,53	0,01183609	**0,044241696**	**0,044174675**
1264,45	917,97	-1,68	0,01193108	**0,044381312**	**0,044241696**
7,58	16,87	2,61	0,0121692	**0,0446204**	**0,0446204**
317,72	317,06	2,02	0,0120827	**0,044729226**	**0,044381312**
213,79	56,33	-1,26	0,0121654	**0,044819895**	**0,044729226**
15,91	30,19	2,12	0,01243402	**0,045375334**	**0,0446204**
265,5	88,55	1,28	0,0127084	**0,045941164**	**0,045941164**
23,03	230,87	-3,73	0,01268877	**0,04608657**	**0,045375334**
143,98	49,3	-1,22	0,01288651	**0,046367349**	**0,045941164**
5,91	20,51	2,31	0,01319002	**0,047238676**	**0,046367349**
147,84	97,79	1,75	0,013268	**0,047297963**	**0,047238676**
20,74	21,01	2,15	0,01359553	**0,048242203**	**0,047297963**
249,68	86,45	-1,26	0,01368265	**0,048328626**	**0,048242203**
36,94	57,7	2,29	0,01377942	**0,048448189**	**0,048328626**
7,04	9,48	1,8	0,0140468	**0,0491638**	**0,048448189**
10,1	17,87	2,05	0,01465715	**0,051067898**	**0,0491638**
13,96	14,89	2,04	0,01504523	**0,051949897**	**0,051949897**
199,62	68,09	1,23	0,0149844	**0,051972919**	**0,051067898**
14,89	47,61	3,33	0,01515376	**0,05209105**	**0,051949897**
112,26	35,46	-1,28	0,01552628	**0,052206269**	**0,052206269**
222,76	129,48	1,91	0,01559667	**0,052214939**	**0,052206269**
27,44	32,04	1,96	0,01550341	**0,052358007**	**0,052358007**
862,9	599,92	-2,17	0,01544661	**0,05239599**	**0,05239599**
48,86	52,72	1,83	0,01532029	**0,052429437**	**0,05209105**
31,79	17,62	1,48	0,01540885	**0,052499179**	**0,052429437**
124,84	79,68	1,52	0,01575926	**0,052530867**	**0,052214939**
428,24	877,91	-1,72	0,01634568	**0,054250748**	**0,052530867**
9217,43	11047,52	-2,41	0,01649239	**0,054502748**	**0,054250748**
877,08	796,58	-1,9	0,01689872	**0,055606899**	**0,054502748**
39,81	26,42	1,7	0,01714719	**0,05618441**	**0,055606899**
64,73	62,7	-2,1	0,01817961	**0,059064556**	**0,059064556**
1346,95	962,02	-1,54	0,01813547	**0,059170813**	**0,05618441**
560,95	1137,26	-2,24	0,01841687	**0,059583991**	**0,059064556**
217,45	117,2	1,56	0,01873433	**0,060357465**	**0,059583991**
3,08	5,3	1,51	0,01897174	**0,060867666**	**0,060357465**
6,3	12,86	2,48	0,01909978	**0,061024193**	**0,060867666**
157,67	70,97	1,37	0,02089378	**0,066480209**	**0,061024193**
22,75	20,18	1,88	0,02106715	**0,06675599**	**0,066480209**
52,64	87,89	2,19	0,02119277	**0,066878823**	**0,06675599**
191,81	71,28	1,37	0,02139768	**0,066976478**	**0,066976478**
100	113,24	2,34	0,02131487	**0,066989591**	**0,066878823**
17,32	21,94	1,81	0,0215733	**0,067252798**	**0,066976478**
234,17	164,26	1,62	0,02179933	**0,067683404**	**0,067252798**
1007,27	493,74	-1,25	0,02268993	**0,069884984**	**0,069884984**

21,95	28,65	2,41	0,02278117	**0,069886458**	**0,069884984**
243,8	84,37	-1,52	0,02293127	**0,070067769**	**0,069886458**
18,42	142,04	-2,3	0,02265873	**0,070069165**	**0,067683404**
19,21	17,11	1,75	0,02320918	**0,070636635**	**0,070067769**
120,15	108,89	2,22	0,02343571	**0,071045263**	**0,070636635**
181,89	110,07	1,39	0,02400099	**0,072473578**	**0,071045263**
37,7	211,64	-2,77	0,02475357	**0,073876934**	**0,073876934**
93,95	46,57	1,43	0,02471234	**0,074040863**	**0,074040863**
1567,25	826,18	-2,38	0,02493035	**0,074117257**	**0,073876934**
5,4	7,62	1,76	0,02470525	**0,07430876**	**0,072473578**
43,48	38,64	1,92	0,02553035	**0,074463521**	**0,074463521**
283,59	115,33	1,46	0,02545249	**0,074518697**	**0,074518697**
79,08	86,9	1,71	0,02521717	**0,074681619**	**0,074117257**
1043,04	407,12	-3,1	0,02541159	**0,074682917**	**0,074682917**
101,18	311,36	2,45	0,02540668	**0,074954573**	**0,074681619**
81,79	367,25	2,19	0,02601505	**0,0755909**	**0,074463521**
89,61	103,63	-1,74	0,02611882	**0,075607111**	**0,0755909**
47,31	19,23	-1,31	0,02651687	**0,076471872**	**0,075607111**
4,71	9,37	2,13	0,02672515	**0,076784946**	**0,076471872**
16,43	71,99	2,42	0,02734188	**0,078264861**	**0,076784946**
10,72	29,42	2,8	0,02975565	**0,084545574**	**0,084545574**
242,31	59,83	-1,17	0,02969705	**0,084691587**	**0,078264861**
69,99	75,11	-1,87	0,03037304	**0,085667549**	**0,085667549**
40,99	35,23	1,83	0,03030314	**0,085784624**	**0,084545574**
297,57	179,22	1,47	0,03101091	**0,086830548**	**0,086830548**
712,23	311,78	-1,23	0,03090131	**0,086839448**	**0,085667549**
214,98	147,06	1,94	0,03206585	**0,089459074**	**0,086830548**
421,27	888,83	2,39	0,03230598	**0,089803627**	**0,089459074**
603,4	517,92	-1,59	0,03353905	**0,09289593**	**0,089803627**
1210,99	2644,18	-1,85	0,0339539	**0,093040936**	**0,093040936**
86,5	184,9	-2,08	0,033925	**0,09329375**	**0,09329375**
483,87	226,02	1,31	0,03392278	**0,093622009**	**0,09289593**
179,9	37,21	-1,16	0,03500174	**0,095572127**	**0,093040936**
44,76	74,03	-2,28	0,03554021	**0,096699511**	**0,095572127**
46,72	283,37	-2,56	0,03610744	**0,097896932**	**0,096699511**
1011,52	367,41	1,41	0,03634498	**0,098195209**	**0,097896932**
17,64	18,78	1,75	0,03652391	**0,098333604**	**0,098195209**
374,24	309,14	-1,33	0,03701863	**0,099318276**	**0,098333604**
643,17	1772,85	-1,92	0,03738761	**0,09995993**	**0,099318276**
4,92	6,37	1,81	0,03754776	**0,100040745**	**0,09995993**
833,41	691,28	1,4	0,03807002	**0,101082467**	**0,100040745**
525,29	1250,75	-2,12	0,03881227	**0,101998116**	**0,101998116**
281,96	426,28	2,01	0,03858518	**0,102098243**	**0,101082467**
44,08	167,68	-1,69	0,03873032	**0,102131323**	**0,102098243**
69,5	115,05	-2,07	0,03953303	**0,103538888**	**0,101998116**
774,26	395,44	-1,66	0,03989902	**0,104143205**	**0,103538888**
99,93	76,48	1,79	0,04006634	**0,104226628**	**0,104143205**
329,12	508,33	1,78	0,04034493	**0,104597967**	**0,104226628**
108,81	108,6	-1,8	0,04110704	**0,106216177**	**0,104597967**
316,83	446,99	-1,68	0,04218927	**0,108647953**	**0,106216177**

1106,3	464,11	1,24	0,04493528	**0,115333885**	**0,108647953**
62,75	74,51	1,71	0,04546805	**0,116313616**	**0,115333885**
253,75	276,61	-1,85	0,04597051	**0,116438463**	**0,116438463**
74,06	66,25	1,74	0,04571933	**0,116569153**	**0,116313616**
35,91	46,83	-1,65	0,04588304	**0,116600465**	**0,116569153**
62,58	56,09	1,84	0,04632892	**0,116961536**	**0,116438463**
327	261	-1,3	0,04654668	**0,117127267**	**0,116961536**
78,51	32,6	1,39	0,04707251	**0,118064602**	**0,117127267**
30,2	21,8	1,59	0,04868198	**0,12170495**	**0,118064602**
280,19	108,86	-1,24	0,04954078	**0,123451135**	**0,12170495**
