# Supplementary material for: Comprehensive transcriptomic analysis of prostate cancer lung metastases
Source: PLoS One. 2024 Aug 15;19(8):e0306525. doi: 10.1371/journal.pone.0306525 (PMC11326543; doi:10.1371/journal.pone.0306525)
Supplement: S2 File — (PDF) [file pone.0306525.s004.pdf]

UNIVERSITÄT ZU LÜBECK

Ethik-Kommission
Vorsitzender:
Herr Prof. Dr. med. Alexander Katalinic
Universität zu Lübeck
Stellv. Vorsitzender:
Herr Prof. Dr. med. Frank Gieseler
Ratzeburger Allee 160
23538 Lübeck

Sachbearbeitung: Frau Janine Erdmann
Tel.: +49 451 3101 1008
Fax: +49 451 3101 1024

ethikkommission@uni-luebeck.de

Universität zu Lübeck · Ratzeburger Allee 160 · 23538 Lübeck

Frau
Dr. med. Verena-Wilbeth Sailer
Institut für Pathologie und
des Forschungszentrums Borstel
Parkallee1-40

23845 Borstel

Aktenzeichen: 18-053
Datum: 02. März 2018

Sitzung der Ethik-Kommission am 01. März
Entwicklung und Validierung eines Genexpressionsassays als prognostischer und prädiktiver Biomarker für Knochenmetastasen des Prostatakarzinoms

Sehr geehrte Frau Dr. Sailer,

der Antrag wurde unter berufsethischen, medizinisch-wissenschaftlichen und berufsrechtlichen Gesichtspunkten geprüft.

Die Kommission hat keine Bedenken.

Bei Änderung des Studiendesigns sollte der Antrag erneut vorgelegt werden.
Über alle schwerwiegenden oder unerwarteten und unerwünschten Ereignisse, die während der Studie auftreten, ist die Kommission umgehend zu benachrichtigen.
Die Deklaration von Helsinki in der aktuellen Fassung fordert in § 35 dazu auf, jedes medizinische Forschungsvorhaben mit Menschen zu registrieren. Daher empfiehlt die Kommission grundsätzlich die Studienregistrierung in einem öffentlichen Register (z.B. unter www.drks.de).
Die ärztliche und juristische Verantwortung des Studienleiters und der an der Studie teilnehmenden Ärzte bleibt entsprechend der Beratungsfunktion der Ethikkommission durch unsere Stellungnahme unberührt.

Mit freundlichen Grüßen

Prof. Dr. med. Alexander Katalinic
Vorsitzender
