# Supplementary material for: Comprehensive transcriptomic analysis of prostate cancer lung metastases
Source: PLoS One. 2024 Aug 15;19(8):e0306525. doi: 10.1371/journal.pone.0306525 (PMC11326543; doi:10.1371/journal.pone.0306525)
Supplement: S1 File — (PDF) [file pone.0306525.s003.pdf]

English Translation of the Ethic Vote:

Date: March 2, 2018

Meeting of the Ethics Committee on March 1st
Development and validation of a gene expression assay as a prognostic and predictive biomarker for bone metastases from prostate cancer

Dear Dr. Sailer,

The application was examined under professional ethics, medical-scientific and professional law aspects.

The Commission has no concerns.

If the study design is changed, the application should be submitted immediately. The commission must be notified immediately of any serious or unexpected undisclosed events that occur during the study. The Declaration of Helsinki in its current version calls for this in Section 35 of every medical research project register with people. The commission therefore generally recommends registration of the study in an available register (e.g. at pras.drka.dig. The medical and legal responsibility of the study director and the doctors participating in the study remain unaffected by our statement in accordance with the advisory function of the ethics committee

Best regards

Prof. Dr. med. Alexander Katalinic
Chairman

UNIVERSITÄT ZU LÜBECK

Universität zu Lübeck · Ratzeburger Allee 160 · 23538 Lübeck

Frau
Dr. med. Verena-Wilbeth Sailer
Institut für Pathologie und
des Forschungszentrums Borstel
Parkallee1-40

23845 Borstel

Ethik-Kommission
Vorsitzender:
Herr Prof. Dr. med. Alexander Katalinic
Universität zu Lübeck
Stellv. Vorsitzender:
Herr Prof. Dr. med. Frank Gieseler
Ratzeburger Allee 160
23538 Lübeck

Sachbearbeitung: Frau Janine Erdmann
Tel.: +49 451 3101 1008
Fax: +49 451 3101 1024

ethikkommission@uni-luebeck.de

Aktenzeichen: 18-053
Datum: 02. März 2018

Sitzung der Ethik-Kommission am 01. März
Entwicklung und Validierung eines Genexpressionsassays als prognostischer und prädiktiver Biomarker für Knochenmetastasen des Prostatakarzinoms

Sehr geehrte Frau Dr. Sailer,

der Antrag wurde unter berufsethischen, medizinisch-wissenschaftlichen und berufsrechtlichen Gesichtspunkten geprüft.

Die Kommission hat keine Bedenken.

Bei Änderung des Studiendesigns sollte der Antrag erneut vorgelegt werden.
Über alle schwerwiegenden oder unerwarteten und unerwünschten Ereignisse, die während der Studie auftreten, ist die Kommission umgehend zu benachrichtigen.
Die Deklaration von Helsinki in der aktuellen Fassung fordert in § 35 dazu auf, jedes medizinische Forschungsvorhaben mit Menschen zu registrieren. Daher empfiehlt die Kommission grundsätzlich die Studienregistrierung in einem öffentlichen Register (z.B. unter www.drks.de).
Die ärztliche und juristische Verantwortung des Studienleiters und der an der Studie teilnehmenden Ärzte bleibt entsprechend der Beratungsfunktion der Ethikkommission durch unsere Stellungnahme unberührt.

Mit freundlichen Grüßen

Prof. Dr. med. Alexander Katalinic
Vorsitzender
