# Supplementary material for: Comprehensive transcriptomic analysis of prostate cancer lung metastases
Source: PLoS One. 2024 Aug 15;19(8):e0306525. doi: 10.1371/journal.pone.0306525 (PMC11326543; doi:10.1371/journal.pone.0306525)
Supplement: Attachment — Submitted filename: review 29224.docx [file pone.0306525.s005.docx]

**Title: Comprehensive transcriptomic analysis of prostate cancer lung metastases**

In this manuscript, the authors have comprehensively explored transcriptomic alterations in PCa lung metastases in comparison to primary PCa and PCa bone metastases. They have reported that upregulation of immunogenic response genes and downregulation of epithelial-mesenchymal transition genes were associated with PCa lung metastases, thereby, suggesting that lung metastases from PCa have a higher immunogenic character, which may increase patients' susceptibility to immunotherapeutic treatments.

The structuring of the manuscript is well-conceived, easy to follow, and the literature survey in the related field is comprehensive. However, the reviewer found some ways to do the manuscript better for the publication purpose.

The comments from the reviewer are listed below:

**Comment 1:**

The article should include a patient demography table.

**Comment 2:**

It is not clear from the manuscript which log2 Fold cut-off value has been considered for determining mRNA up- and down-regulation. Additionally, the author should include a table or a supplementary Excel file listing all upregulated and downregulated genes.

**Comment 3:**

The dysregulated candidate gene expression (Top 10 up and down) should have been validated by a secondary technique (say qRT-PCR) in the same sample-set.

**Comment 4:**

The authors can be clearer in explaining the choice of chemotactic genes in the result section under the subheading "Altered pathways and biological processes in the metastatic microenvironment," since Figure 2a shows additional altered pathways and biological processes in the metastatic microenvironment.
