# Supplementary material for: Comprehensive transcriptomic analysis of prostate cancer lung metastases
Source: PLoS One. 2024 Aug 15;19(8):e0306525. doi: 10.1371/journal.pone.0306525 (PMC11326543; doi:10.1371/journal.pone.0306525)
Supplement: Attachment — Submitted filename: Response to Reviewer #2.docx [file pone.0306525.s006.docx]

*Responses to the respected reviewer #2 from the authors*

*(Manuscript ID: PONE-D-23-41725, “Comprehensive transcriptomic analysis of prostate cancer lung metastases”*

*Dear Reviewer,*

*Thank you very much for taking the time to review this manuscript. We sincerely appreciate your warm thoughts and comments. Here are the comprehensive point-by-point answers to your comments, accompanied with the highlighted tracked changes in the revised manuscript and re-submitted files where appropriate.*

*Your sincerely,*

*The Authors*

**Point by Point Response to the Reviewer #2:**

**In this manuscript, the authors have comprehensively explored transcriptomic alterations in PCa lung metastases in comparison to primary PCa and PCa bone metastases. They have reported that upregulation of immunogenic response genes and downregulation of epithelial-mesenchymal transition genes were associated with PCa lung metastases, thereby, suggesting that lung metastases from PCa have a higher immunogenic character, which may increase patients' susceptibility to immunotherapeutic treatments.**

**The structuring of the manuscript is well-conceived, easy to follow, and the literature survey in the related field is comprehensive. However, the reviewer found some ways to do the manuscript better for the publication purpose.**

**The comments from the reviewer are listed below:**

**Comment 1.** **The article should include a patient demography table.**

*Response 1. We appreciate this precious reviewer's suggestion. We have now provided a patient demography table with all available details and inserted as “Table 1” in the section “method materials” part “cohort” line 98-100 accordingly in the revised manuscript.*

**Comment 2.** **It is not clear from the manuscript which log2 Fold cut-off value has been considered for determining mRNA up- and down-regulation. Additionally, the author should include a table or a supplementary Excel file listing all upregulated and downregulated genes.**

*Response 2. We thank to the reviewer for this attentive comment. As we can see in the figure 1:*

*Log2FC>1 or Log2FC<-1 are considered to be the cut-offs for mRNA upregulation and downregulation accordingly.*

*For raw ratio data >1 and <-1 are used for determine the up- and down-regulation.*

*Additionally, a comprehensive excel file as supplementary table 1 (S1) is already uploaded represented a list of all up and down regulated genes and detailed annotation for each gene (kindly please refer to the supplementary S1). We also created and uploaded a supplementary excel file (S2) represented our data based on the adjusted p value or FDR (kindly please refer to the supplementary S2).*

**Comment 3. The dysregulated candidate gene expression (Top 10 up and down) should have been validated by a secondary technique (say qRT-PCR) in the same sample-set.**

*Response 3. We again appreciate this reviewer's comment. Nanostring ^TM^ is an alternative to traditional microarray technology, RNA-seq as well as qPCR. Based on the several publications currently Nanostring ^TM^ nCounter technology has been proposed as “a robust, simple, fast and highly sensitive alternative for qPCR” where the number of target genes are very high (eg, PMID: 36555223). According to the official manual manual nCounter Pro Analysis System User Manual (nanostring.com), the principle of NanoString technology is based on the direct measurement of the barcode of the fluorescent molecule on the probe after the nucleic acid molecule hybridizes to the probe. we only need to measure the purity of mRNA and even we do not need to test the RNA integrity number to obtain samples that meet the standard criteria for the quality requirements.*

*Additionally, in our previous publication (Saraji et al., 202, PMID: 34529723) we have reported a comparative and comprehensive analyses for RNA purity and integrity between classical bioanalyzers (Qubit* *^TM^, nanodrop^®^) and nanosting and there we showed that the expressed genes which is produced by nanostiong output already passed the RNA quality criteria including RIN, RNA binding density and RNA fragmentation threshold.*

*However, to welcome the reviewer’s comment we would inform that, behind the scope of this current study which is mostly focused on in-silico analyses, in our future in-vitro and in-vivo studies, the regulation of theses’ genes and even their protein (10 Top and down) will be validated by using Multiplexed-PCR in both cell and tissue level.*

**Comment 4. The authors can be clearer in explaining the choice of chemotactic genes in the result section under the subheading "Altered pathways and biological processes in the metastatic microenvironment," since Figure 2a shows additional altered pathways and biological processes in the metastatic microenvironment.**

*Response 4. We again appreciate this reviewer's attentive comment. We sincerely regret that the incorrect font size was accidentally indicated while identifying the figure addressing and heading. Actually, the heading* ***"Altered pathways and biological processes in the metastatic microenvironment"*** *and* ***“Regulation of chemotaxis”*** *are totally two separated headings. The first heading only included fig 2a and 2b which is representing (a) “Bubble plot of the key sub-cluster functional enrichment created by Metascape represents” and (B). “Protein-protein interactome network of DEGs representing sub-clusters with the highest MCODE scores”.*

*We now corrected the font size of both separated headings and their figure addressing in the results part which is highlighted in the revised manuscript with tracked changes (lines 173-179).*

*Your sincerely,*

*The Authors*
