# Supplementary material for: Comprehensive transcriptomic analysis of prostate cancer lung metastases
Source: PLoS One. 2024 Aug 15;19(8):e0306525. doi: 10.1371/journal.pone.0306525 (PMC11326543; doi:10.1371/journal.pone.0306525)
Supplement: Attachment — Submitted filename: Response to Reviewer One.docx [file pone.0306525.s007.docx]

*Responses to the respected reviewer #1 from the authors*

*(Manuscript ID: PONE-D-23-41725, “Comprehensive transcriptomic analysis of prostate cancer lung metastases”*

*Dear Reviewer One,*

*Thank you very much for taking the time to review this manuscript. We sincerely appreciate your warm thoughts and comments. Here are the comprehensive point-by-point answers to your comments, accompanied with the highlighted tracked changes in the revised manuscript and re-submitted files where appropriate.*

*Your sincerely,*

*The Authors*

**Point by Point Response to the Reviewer #1:**

**The study by Saraji et al. demonstrated the comprehensive transcriptomic analysis of prostate cancer lung metastases. The authors should modify the manuscript by considering following points.**

**Comment 1. The authors have used FFPE samples of prostate metastases samples and compared transcriptome profile of bone metastases and lung metastases samples. But authors have not used any control/adjacent control specimens to derive the specific differentially expressed genes between the subsets. Justify.**

*Response 1. We appreciate this precious reviewer's comment. We sincerely regret the unwelcome disparity! As a reference and control, primary PCa was accidentally reported as "PCa bone metastasis" in the result section, despite the fact that we had already noted this in the abstract and conclusion sections! Actually, for this study we have taken primary PCa as reference* “*setting primary prostate cancer” as a reference versus PCa lung metastases samples but meanwhile we also compared our results from PCa lung metastases to PCa bone metastasis and there we found that 209 genes were significantly upregulated, and 100 genes were significantly downregulated in prostate cancer lung metastases as we described in the result part.*

*We apologize for the mistake in mentioning mentioned bone metastases instead of primary PCa! In order to correct the above-mentioned discrepancy and mistakes, we have now corrected and inserted the number of our primary PCa FFPE as control in the part “Method-material/Cohort” line 89.90 and also correction in the part “Result-1” in the lines 152 accordingly in the revised manuscript.*

**Comment 2. Regarding the statistical analysis of gene expression data, adjusted p value should be calculated to identify the differentially expressed genes in each category of samples.**

*Response 2. We thank the reviewer's comments. Given that the FDR (False discovery rate) technique improved and adjusted the features of the p-value distribution. Furthermore, FDR helped us filter out a subset of DEGs while preserving the genes of interest (supplementary table 1). However, one point we want to emphasise specifically is that if the FDR value > 0.05 and P value < 0.05, we will still use the p-value. Additionally, according to the Nanostring ^TM^ facilities while using nsolver to calculate all samples one can chose the FDR option output as the adjusted p-value.*

*In order to answer this comment in another way, we also used the Benjamini-Hochberg’s method. Furthermore, we then used following formula to calculate the initial FDR, which is expressed as* ***FDR and also correlated FDR*** *in the supporting information table 2 (supplementary table 2).*

*FDR(x)=P(x)*m/x*

*According to the value of X (rank value of p-value) from large to small,*

*FDR(x)=min {FDR(x), FDR(x+1)}*

*We now created and uploaded a supplementary excel file named 2 represented our data based on the adjusted p value or FDR. Please refer to the supplementary 2 for the details.*

**Comment 3. mRNA count should be expressed as RPKM/FPKM which is the standard protocol for RNA sequencing data.**

*Response 3. We again appreciate this reviewer's comment.* *Nanostring ^TM^ is an alternative to traditional microarray technology and RNA-seq technology. The principle of Nanostring ^TM^ technology is based on the direct measurement of the barcode of the fluorescent molecule on the probe after the nucleic acid molecule hybridizes to the probe.*

*According to the official manual* of nCounter; [*nCounter Pro Analysis System User Manual (nanostring.com)*](https://university.nanostring.com/ncounter-pro-analysis-system-user-manual)*, we can use the companion software in order to obtain differentially expressed genes without converting the raw count expression matrix to RPKM/FPKM (e.g., E.Shenderov et al., 2023, PMID: 37012549). In addition, statistically speaking, regardless of whether FPKM is a necessary presentation method, we only used gene expression data in our experiments for differential analysis, and we did not use these data for survival analysis. However according to Nanostring again, variance analysis does not require FPKM normalization of the original data. In this study we only use gene expression matrix to analyse the expression differences of each gene* (*PMID: 37012549).*

**Comment 4. Here, RNA integrity with at least 90% of the fragments longer than 100 nucleotides were considered as suitable for gene expression analysis. Instead of the parameter, RIN should be calculated for the samples which is the standard protocol for gene expression analysis.**

*Response 4. We again appreciate this reviewer's comment. As we have also mentioned in the Response 4, according to the standard protocol provided by the manual nCounter Pro Analysis System User Manual (nanostring.com), we only need to measure the purity of mRNA and do not need to test the RNA integrity number to obtain samples that meet the standard criteria for the quality requirements.*

*However, in our previous publication (Saraji et al., 202, PMID: 34529723) we have already reported a comparative and comprehensive analyses for RNA purity and integrity between classical bioanalyzers (Qubit* *^TM^, nanodrop^®^) and Nanostring ^TM^ and there we showed that in Nanostring ^TM^ the expressed genes which is produced by nCounter output already passed the RNA quality criteria including RIN, RNA binding density and RNA fragmentation threshold (Saraji et al., 202, PMID: 34529723).*

**Comment 5. The resolution of the figures are very poor. That should be modified.** *Response 5. We thank the reviewer for this attentive comment. In order to ensure the quality and clarity of the figures, we have now enhanced the resolution of our figures in a maximum possible quality (600dpi) and re-corrected the figures quality with using “PACE” software and re-uploaded them again on the online system accordingly.*

*Your sincerely,*

*The Authors*
